# Synthesis of propargyl silanes from terminal alkynes *via* a migratory Sonogashira reaction†

**DOI:** 10.1039/d3cc01847d

**Published:** 2023-05-31

**Authors:** Mikus Puriņš, Lucas Eichenberger, Jérôme Waser

**Affiliations:** a Laboratory of Catalysis and Organic Synthesis and National Centre of Competence in Research (NCCR) Catalysis, Institute of Chemical Sciences and Engineering, Ecole Polytechnique Federale de Lausanne, EPFL Lausanne 1015 Switzerland jerome.waser@epfl.ch

## Abstract

Herein we report a mild synthesis of propargyl silanes from terminal alkynes. We exploit a bromonaphthyl-substituted silane as a silylmethyl electrophile surrogate, which participates in a Sonogashira reaction after an aryl-to-alkyl Pd-migration. Twenty-seven propargyl silanes were obtained in up to 88% yield. The obtained products were versatile building blocks that can be used in addition to electrophiles, triple bond hydrogenation or silyl group cleavage with acid or fluoride sources.

## Abstract 2

A Pd-catalyzed migratory Sonogashira reaction has been developed for accessing propargyl silanes starting from terminal alkynes under mild conditions.

## Graphical abstract

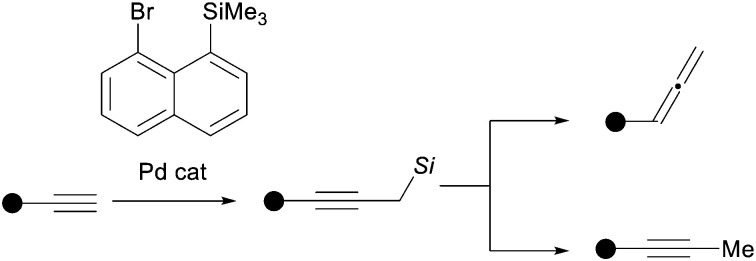

Propargyl silanes represent an important class of organosilicon compounds.^1^ They react with electrophiles at the γ-carbon with the loss of the silicon group to yield allenyl products (Scheme 1A, eqn (1)).^2,3^ Alternatively, a 1,2 silyl shift can occur to give annulated products still bearing the silyl group (Scheme 1A, eqn (2)).^4,5^ Yet, propargyl silanes are relatively difficult to access. One approach is to introduce a silyl group at the propargylic position of an alkyne *via* an electrophilic silylation (Scheme 1B, eqn (3)). However, this process requires highly basic propargylmetal reagents, which often isomerize *via* a propargyl-allenyl equilibrium, and thereby lacks chemoselectivity and functional group tolerance.^6–10^ Other strategies involve the nucleophilic silylation of enynes (Scheme 1B, eqn (4))^11^ or the construction of the alkyne adjacent to an existing silyl group, *e.g.*, starting from α-silyl aldehydes (Scheme 1B, eqn (5)).^12,13^ While these and other methods^14^ can deliver complex propargyl silanes, they rely on pre-functionalized substrates or multi-step sequences.

**Scheme 1:**
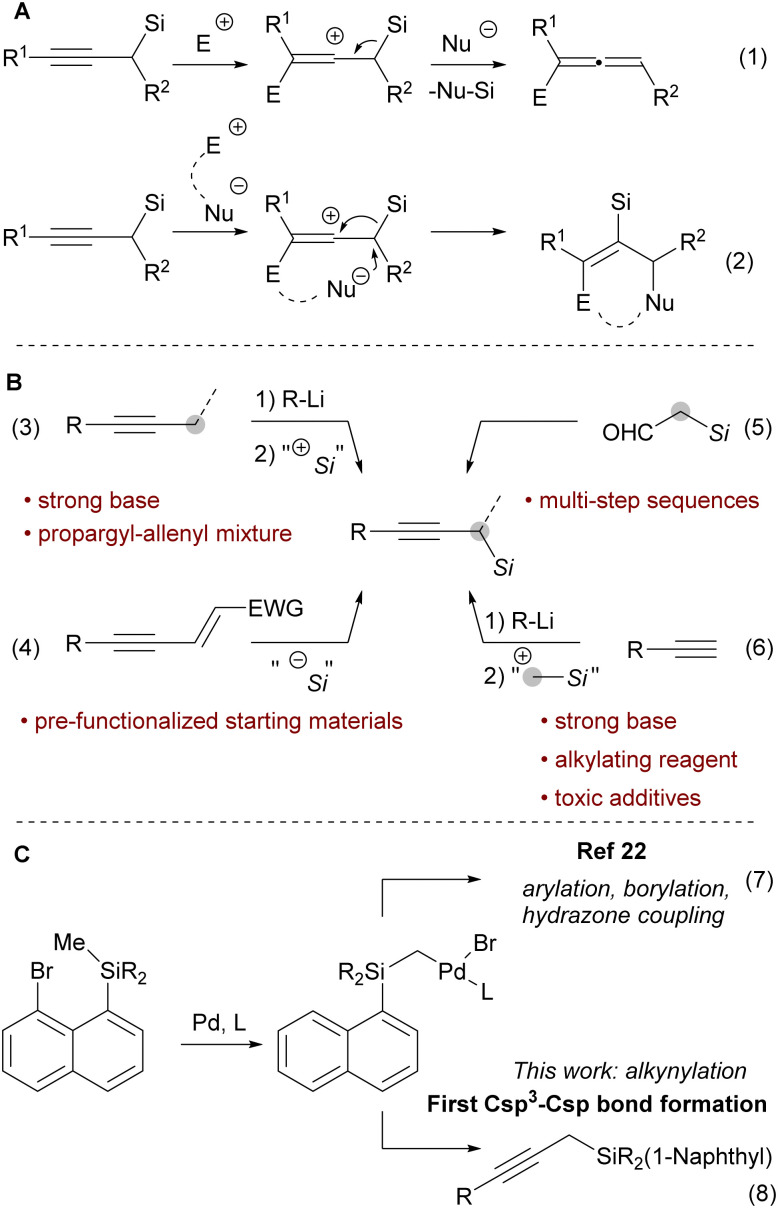
(A) Reactivity of propargyl silanes. (B) Common approaches for the synthesis of propargyl silanes. (C) 1,5-Aryl to alkyl Pd-migration/cross coupling cascades.

In contrast, the direct conversion of widely available terminal alkynes to propargyl silanes is a conceptually simple and attractive approach (Scheme 1B, eqn (6)).^15–18^ This strategy is usually performed by deprotonation of terminal alkynes and then a reaction with a silylmethyl electrophile, such as trimethylsilylmethyl iodide.

However, the use of strong bases and electrophilic alkylating reagents contribute to low functional group tolerance.^19^ In addition, this approach often requires the toxic hexamethylphosphoric triamide^20^ or tetramethylethylene diamide^21^ as additives. The constraints of the existing methods impede access to functionalized propargyl silanes and thus discourage their application in organic synthesis. Therefore, a milder strategy to achieve the conversion of terminal alkynes to propargyl silanes is highly desirable.

Recently, the Zhao group developed a novel approach to introduce a silylmethyl group (Scheme 1C, eqn (7)).^22^ Upon oxidative addition into a bromonaphthalenesilane, a 1,5-aryl to alkyl Pd migration occurred. The Pd-alkyl intermediate could then be intercepted with a carbene generated from *N*-tosylhydrazone, an aryl boronic acid or bis(pinacolato)diboron to yield vinyl, benzyl or borylmethyl silanes. However, Csp^3^–Csp bonds were never made using this approach. We speculated that the combination of the proposed silylmethyl-Pd species with terminal alkynes as the nucleophiles^23^ would result in a simple approach towards propargylic silanes (Scheme 1C, eqn (8)). In this work we report the successful implementation of this concept.

We chose phenylacetylene (1a) as the model substrate and 8-bromonaphthyltrimethyl silane (2) as the model silylmethyl donor. With the previously reported SPhos as the ligand, in the presence of copper (I) iodide and triethylamine as the base, product 3a was formed in 77% yield (Table 1, entry 1). Upon screening of various ligands, we identified DavePhos as a superior ligand, giving 3a in 92% yield (entry 2, for full details see Table S1 in the ESI†). Surprisingly, we found that the copper salt is not required in this transformation (entry 3, see Table S2 in the ESI† for other control experiments). The reaction could be smoothly scaled up to 0.4 mmol scale, and the product was isolated in 82% yield (entry 4). To further demonstrate the practicality of our approach, we chose to investigate 4-butyn-1-ol (4a) as a substrate. Although products similar to 5a have been previously reported,^24^ expensive propargyltrimethyl silane^25^ and gaseous oxirane were used as starting materials. Using the conditions optimized for phenylacetylene (1a), the desired product 5a was obtained in 34% yield (entry 5). We found that in this case K_3_PO_4_ as base is beneficial (42% yield, entry 6). Using SPhos as the ligand gave propargyl silane 5a in 63% yield (entry 7). The copper additive was re-evaluated, but a slightly lower 54% yield of 5a was obtained (entry 8). Finally, on 0.4 mmol scale 5a was obtained in 65% NMR yield and 58% isolated yield (entry 9).

**Table 1:** Optimization of the reaction conditions

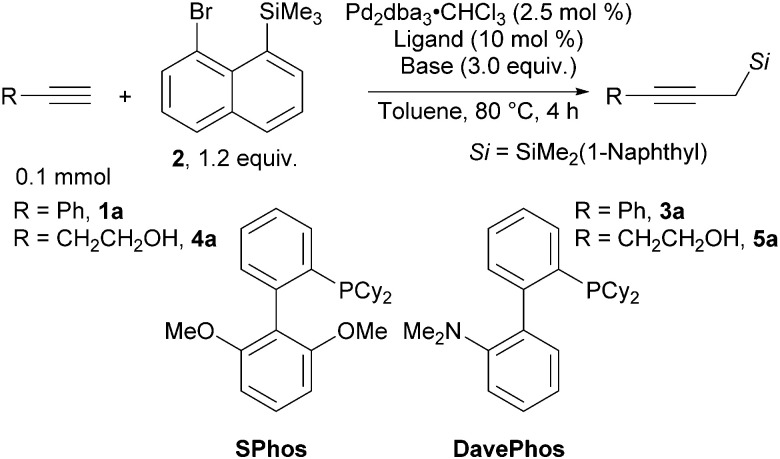				
Entry	R	Ligand	Base	Yield^a^ (%)
1^b^	Ph	SPhos	Et_3_N	3a, 77
2^b^	Ph	DavePhos	Et_3_N	3a, 92
3	Ph	DavePhos	Et_3_N	3a, 88
4^c^	Ph	DavePhos	Et_3_N	3a, 91 (82)^d^
5	CH_2_CH_2_OH	DavePhos	Et_3_N	5a, 34
6	CH_2_CH_2_OH	DavePhos	K_3_PO_4_	5a, 42
7	CH_2_CH_2_OH	SPhos	K_3_PO_4_	5a, 63
8^b^	CH_2_CH_2_OH	SPhos	K_3_PO_4_	5a, 54
9^c^	CH_2_CH_2_OH	SPhos	K_3_PO_4_	5a, 65 (58)^d^

a NMR yields determined using trichloroethylene (1.0 equiv.) as internal standard.

b With CuI (5 mol %).

c On 0.4 mmol scale.

d Isolated yield.

Various terminal alkynes were well tolerated in the migratory Sonogashira reaction. For example, electron donating substituents placed in the *para* position to the alkyne provided the propargylic silanes 3b–3e in 65–87% yields (Scheme 2). Interestingly, in this process a free aniline functionality, which would react under highly basic and alkylating conditions, is tolerated. In contrast, only a trace of product was observed when *para*-hydroxyphenyl acetylene was used (for unsuccessful substrates see section D.5. in the ESI†). Additionally, alkynes with electron poor functional groups can be used in this reaction to give products 3f to 3k in 43–89% yields. The structural identity of the products was further confirmed by the single crystal X-Ray structure of nitrile 3h (ccdc number: 2242318, see section E in the ESI†). This process tolerates electrophilic functional groups within the starting material, such as a nitrile, an aldehyde or an ester functionality. These moieties could be susceptible to side reactions, if full deprotonation of the alkyne would be required for the generation of the propargylic silane. In particular, acetylide addition to aldehydes is a well-established propargyl alcohol synthesis method.^26^ Furthermore, halogen substituents, such as *para*-chloro and a fluorine in *para*, *meta* or *ortho* positions were tolerated to give products 3n to 3q in 54 to 79% yields. *Para*-bromine was not tolerated on the arene, likely due to a competitive reaction with Pd(0) species. Finally, various heterocycles were tolerated under our reaction conditions. 4-Pyridyl, 3-pyridyl and 2-pyridyl acetylenes provided the products 3r–3t in 27–60% yield. Alkynes with a 2-thiophenyl and 3-thiophenyl substitution gave the products 3u and 3v in 54% and 78% yields.

**Scheme 2:**
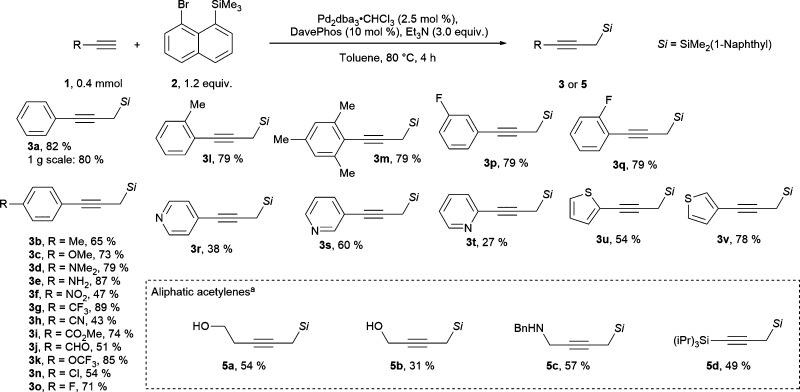
Scope of the migratory Sonogashira reaction. ^a^ With SPhos (10 mol %) and K_3_PO_4_ (3.0 equiv.).

Non-aromatic terminal alkynes were more challenging. Generally, only substrates with a potentially coordinating functionality on the alkyne provided products in synthetically useful yields. Thus, propargyl silanes 5a–5c with alcohol or *N*-benzyl substitution were obtained in 31 to 57% yield (Scheme 2). It is worth to note that these substrates may not be suitable for “classical” conditions of propargyl silane synthesis from terminal alkynes. For example, extra caution would be required for deprotonation of alkynes with acidic heteroatom substituents. In addition, the nucleophilic lone pairs could react with highly electrophilic reagents. Notably, TIPS-substituted acetylene provided bis-silane 5d in 49% isolated yield. Other substituents, such as alkyl, ester, NHBoc, carboxylic acid and ester, did not provide the desired products in reasonable yields. In most of the cases no terminal alkyne could be observed after the reaction, implying that in this case non-specific degradation pathways are faster than the desired transformation. The less activated aliphatic acetylenes are historically poorer substrates for Sonogashira coupling when compared with aromatic acetylenes.^27^

To demonstrate the utility of the naphthyl substituted propargyl silanes, several product modifications were performed. Propargyl silane 3a was added to glucal 6 in the presence of the strong Lewis acid BF_3_·OEt_2_ giving allene 7 in 48% yield (Scheme 3A, eqn (9)).^28^ The triple bond of the alkyne 3a can be semi-hydrogenated to give allyl silane 8 in 74% yield, or fully reduced to aliphatic silane 9 in quantitative yield (Scheme 3A, eqn (10)).^14^ The silyl group can also be easily cleaved – treatment with TfOH yielded the corresponding allenes 10 (Scheme 3B).^29^ This process is efficient with electron withdrawing or neutral substituents on the aromatic ring. However, starting with the electron donating *para*-methoxy substituted alkyne 3c, allene 10c was not observed. Instead an allyl silane byproduct was formed arising from protonation of the triple bond in the opposite direction (see sections D.12. and D.13. in the ESI† for details). In contrast, the use of TBAF cleaved the silyl group whereas keeping the alkyne functionality intact to give acetylenes 11 in 60–88% yield (Scheme 3C).^30^ Electron withdrawing groups are essential also in this case. With electron neutral or electron donating substituents, an inseparable mixture of the alkynes 11 and the allenes 10 was observed. Thus, our method can be considered a divergent 2-step 1-carbon homologation process of terminal alkynes to give either allenes or alkynes.

**Scheme 3:**
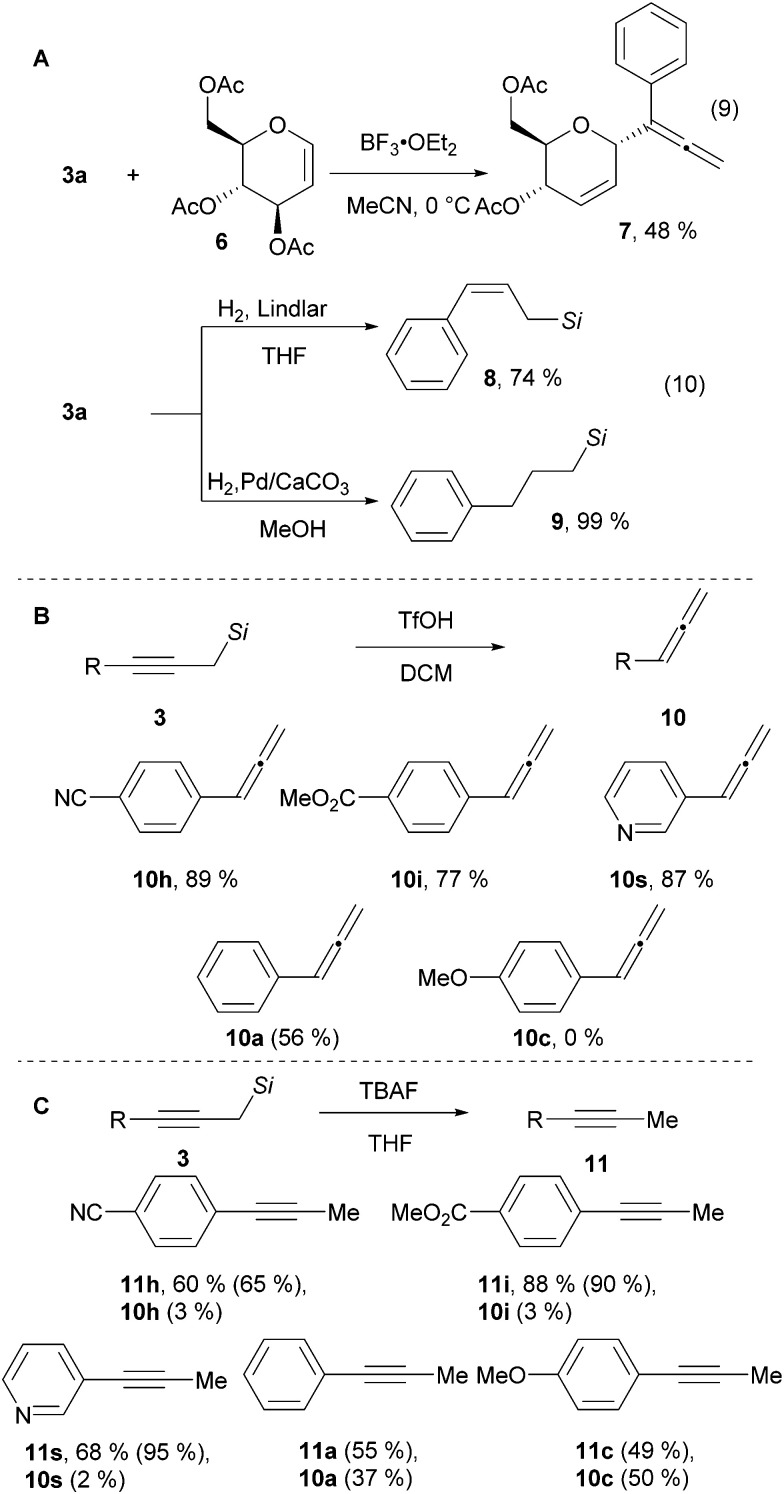
Utility of 1-naphthyl propargylic silanes. NMR yields are indicated in parenthesis. *Si* = SiMe_2_(1-Naphthyl).

In conclusion, we have developed a migratory Sonogashira reaction for the conversion of terminal alkynes to propargylic silanes under mild conditions. Our approach exploits an aryl to alkyl Pd-migration process to give a silylmethyl-Pd species, which then reacts with terminal alkynes. Therefore, no highly electrophilic reagent is required and the tolerance of nucleophilic functionalities was improved. In addition, the catalytic activation of the alkyne *via* a copper-free Sonogashira process avoided the use of strong bases. The obtained products can be used in addition reactions to electrophiles and the silicon group can be cleaved under mild conditions. In particular, the migratory Sonogashira/silyl cleavage sequence can be used as a mild and divergent 2-step 1-carbon homologation process to obtain either an allene or a methyl alkyne from a terminal alkyne.^31^

M. P. planned and supervised the research, prepared the manuscript and corrected the supporting information. L. E. in close supervision by M. P. performed the experiments and prepared the supporting information. J. W. supervised the project, corrected and edited the manuscript and proofread the supporting information.

This work is supported by the European Research Council (ERC Consolidator Grant SeleCHEM, No. 771170). We thank Dr Rosario Scopelliti and Dr Farzaneh Fadaei Tirani from ISIC at EPFL for X-ray analysis. This publication was created as a part of NCCR Catalysis, a National Center of Competence in Research funded by the Swiss National Science Foundation (Grant No. 180544).

## Conflicts of interest

There are no conflicts to declare.

## Supplementary Material

CC-059-D3CC01847D-s001
