# Supplementary material for: Synthesis of propargyl silanes from terminal alkynes via a migratory Sonogashira reaction
Source: Chem Commun (Camb). 2023 May 31;59(51):7931–4. doi: 10.1039/d3cc01847d (PMC10286695; doi:10.1039/d3cc01847d)
Supplement: CC-059-D3CC01847D-s001 [file CC-059-D3CC01847D-s001.pdf]

Electronic Supplementary Material (ESI) for ChemComm.
This journal is © The Royal Society of Chemistry 2023

Supporting Information for

"Synthesis of Propargyl Silanes from Terminal Alkynes via a Migratory Sonogashira Reaction"

Mikus Puriņš, Lucas Eichenberger and Jerome Waser*

Laboratory of Catalysis and Organic Synthesis, Ecole Polytechnique Fédérale de Lausanne, EPFL, SB ISIC LCSO, BCH 4306, 1015 Lausanne (Switzerland)

*Correspondence to: jerome.waser@epfl.ch

Table of Contents

A. General Information S3

B. Synthesis of the Starting Materials and Ligands S4

B.1. Synthesis of the Propargylic Silanes Precursor S4

C. Optimization Studies S6

Table S1. Influence of the ligand S6

Table S2. Control reactions S6

Table S3. Influence of the solvent and the base S7

Table S4. Fine tuning of the reaction conditions S7

Table S5. Influence of the solvent S7

Table S6. Influence of the base S8

Table S7. Influence of the Pd source S8

Table S8. Influence of the ligand S8

Table S9. Fine tuning of the reaction conditions S9

Table S10. Influence of the ligand for But-3-yn-1-ol S9

Table S11. Fine tuning of the reaction conditions for But-3-yn-1-ol S9

D. Procedures and product characterization data of propargyl silanes S10

D.1. General Procedure for the migratory Sonogashira reaction with aryl substrates. S10

D.2. Characterization of the aryl propargyl silanes. S10

D.3. General Procedure for the migratory Sonogashira reaction with aliphatic alkynes. S17

D.4. Characterization of the aliphatic propargyl silanes. S17

D.5. Unsuccessful substrates. S18

D.6. Addition of propargylic silane to glucal S18

D.7. Hydrogenations of the triple bond S19

D.8. General Procedure for conversion of propargyl silanes to allenes. S20

D.9. Characterization data of the allenes S20

D.10. General Procedure for conversion of propargyl silanes to methyl alkynes. S21

D.11. Characterization of methyl alkynes S21

D.12. Byproduct of electron rich propargyl silane reaction with electrophile S22

D.13. Degradation study of the electron rich propargyl silane reaction with electrophile product S23

E. X-Ray Crystallographic Data S24

F. References S25

G. NMR Spectra S26

G.1. Propargyl silanes. S26

G.2. Product modification. S56

A. General Information

The NMR spectra were recorded on a Brucker DPX-400 spectrometer at 400 MHz for ^{1}H, 101 MHz for ^{13}C, 376 MHz for ^{19}F. The chemical shift (δ) for ^{1}H and ^{13}C are given in ppm relative to residual signals of the solvents (chloroform-d - 7.26 ppm ^{1}H NMR and 77.16 ppm ^{13}C NMR; methanol-d4 3.31 ppm ^{1}H NMR and 49.0 ppm ^{13}C NMR; dmso-d6 2.50 ppm ^{1}H NMR and 39.52 ppm ^{13}C NMR). Carbon spectra have been measured using broadband {^{1}H} decoupling. Coupling constants are given in Hertz. The following abbreviations are used to indicate the multiplicity: s, singlet; d, doublet; q, quartet; m, multiplet; bs, broad signal; app, apparent. Infrared spectra were recorded on a JASCO FT-IR B4100 spectrophotometer with an ATR PRO410-S and a ZnSe prisma and are reported as cm^{-1} (w = weak, m = medium, s = strong, br = broad). High resolution mass spectrometric measurements were performed by the mass spectrometry service of ISIC at the EPFL on a MICROMASS (ESI) Q-TOF Ultima API. The raw data obtained from the Q-TOF Waters instrument does not take into account the mass of the electron for the ion, the obtained raw data has been therefore corrected by removing the mass of the electron (5 mDa).

The diffraction data for crystal structures were collected by mass spectrometry service of ISIC at the EPFL at low temperature using Cu (323) or Mo (520) K_a radiation on a Rigaku SuperNova dual system in combination with Atlas type CCD detector. The data reduction and correction were carried out by *CrysAlis^{Pro}* (Rigaku Oxford Diffraction, release 1.171.40.68a, **2019**). The solutions and refinements were performed by *SHELXT*[1] and *SHELXL*[2], respectively. The crystal structures were refined using full-matrix least-squares based on F^2 with all non-H atoms defined in anisotropic manner. Hydrogen atoms were placed in calculated positions by means of the "riding" model. Yields of isolated products refer to materials of >95% purity as determined by ^{1}H NMR.

The authors are indebted to the team of the research support service of ISIC at EPFL, particularly to the NMR, X-Ray, and the High Resolution Mass Spectrometry Units.

General Procedures. All reactions were set up under a nitrogen atmosphere in oven-dried glassware using standard Schlenk techniques, unless otherwise stated. Synthesis grade solvents were used as purchased; anhydrous solvents (THF, Et_2O, Toluene and DCM) were taken from a commercial SPS solvent dispenser (H_2O content < 10 ppm, *Karl-Fischer* titration). Chromatographic purification of products was accomplished using flash chromatography (FC) on SiliaFlash P60 silica gel (230 - 400 mesh) or using Biotage Isolera Spektra One with pre-packaged silica cartridges purchased from Buchi, models: Sepacore or GraceResolve (4 g, 12 g, 25 g, 40g, 80g, 120g) or BÜCHI Pure C-810 Flash system with Reverse Phase (RP) C18 columns. For thin layer chromatography (TLC) analysis throughout this work, Pre-coated TLC sheets ALUGRAM® Xtra SIL G/UV_{254} were employed, using UV light as the visualizing agent and basic aqueous potassium permanganate ($KMnO_4$) stain solutions, and heat as developing agents. Organic solutions were concentrated under reduced pressure on a Büchi rotatory evaporator.

Materials. Terminal alkynes **1a**, **1b**, **1d**, **1e**, **1g**, **1i**, **1j**, **1l**, **1r**, **1t**, **1v**, **4a**, **4b** and **4d** were purchased from Sigma-Aldrich, **1o**, **1p** and **1s** from Fluorochem, **1h** and **1m** from ABCR, **1k** and **1u** from Apollo and **1c**, **1f**, **1q** and **1n** from TCI. Tris(dibenzylideneacetone)dipalladium was purchased from Fluorochem and recrystalised in 200 mg portions following a reported procedure.[3] The synthesis of starting material **4c** has already been described by our group. The procedures are taken from the indicated publications[4] for clarity and to facilitate the reproduction of the results.

B. Synthesis of the Starting Materials and Ligands

B.1. Synthesis of the Propargylic Silanes Precursor

(8-Bromonaphthalen-1-yl)trimethylsilane (2)

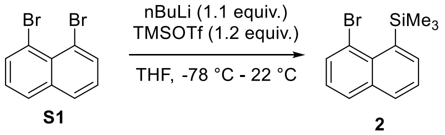

Scheme S1. Synthesis of bromo trimethyl silane naphthalene S1.

According to a reported procedure,[5] a flame-dried 250 mL round-bottom flask was charged with 1,8-dibromonaphthalene (10.0 g, 35.0 mmol, 1.0 equiv) and evacuated/backfilled with N_2 3 times. Then, THF (70 mL) was added, the mixture was cooled to -78 °C and nBuLi (2.5 M in THF; 15.4 mL, 38.5 mmol, 1.1 equiv.) added drop-wise. The reaction mixture was stirred at this temperature for 0.5 h and then trimethylsilyl triflate (7.6 mL, 42 mmol, 1.2 equiv.) was added drop-wise. The solution was then allowed to reach room temperature and was stirred for 1 h. The reaction mixture was cooled to 0 °C and the reaction was quenched with $NaOH_{(aq)}$ (2 M, 70 mL). The product was extracted with Et_2O (3×50 mL). The combined organic layers were dried on $MgSO_4$ and concentrated *in vacuo*. The crude product was purified by column chromatography using pentane as eluent to afford the (8-bromonaphthalen-1-yl)trimethylsilane as a white solid (8.1 g, 29 mmol, 83 % yield).

^{1}H NMR (400 MHz, Chloroform-*d*) δ 8.00 (dd, *J* = 7.0, 1.4 Hz, 1H, Ar*H*), 7.88 (dd, *J* = 7.3, 1.4 Hz, 1H, Ar*H*), 7.82 (ddd, *J* = 7.9, 5.3, 1.4 Hz, 2H, Ar*H*), 7.44 (dd, *J* = 8.1, 7.0 Hz, 1H, Ar*H*), 7.29 (dd, *J* = 8.1, 7.4 Hz, 1H, Ar*H*), 0.58 (s, 9H, Si(C*H*$_3$)$_3$).
^{13}C{^{1}H} NMR (101 MHz, Chloroform-*d*) δ 138.8, 137.8, 137.2, 136.2, 132.5, 131.0, 129.6, 125.8, 125.3, 122.5, 4.7.

Spectral data were consistent with the values reported in literature. [5]

4-Ethynylphenol (S2)

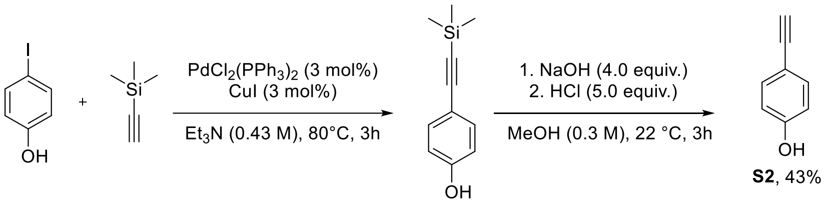

Scheme S2. Synthesis of 4-ethynylphenol **S2**.

According to a reported procedure,[6] ethynyl(trimethyl)silane (206 mg, 291 µL, 2.10 mmol, 1.4 equiv) was added to a solution of 4-iodophenol (330 mg, 1.50 mmol, 1.0 equiv), Bis(triphenylphosphine)palladium(II) dichloride (10.5 mg, 15.0 µmol, 0.010 equiv) and copper (I) iodide (2.86 mg, 15.0 µmol, 0.0100 equiv) in Et_3N (3.5 mL), and the mixture was refluxed at 80 °C for 3 h under nitrogen. The reaction mixture was filtered through a plug of silica and concentrated *in vacuo*. The crude material was purified by flash chromatography (0-50% (v/v) EtOAc/pentane), to afford 4-((trimethylsilyl)ethynyl)phenol as a brown oil (242 mg, 1.27 mmol, 84%).

Aqueous NaOH (5 N, 2 mL) was added to a solution of 4-((trimethylsilyl)ethynyl)phenol (242 mg, 1.27 mmol, 1.0 equiv.) in MeOH (4 mL) and the mixture was stirred under nitrogen **at 22 °C for 3 h**, then neutralized with conc. HCl and extracted with DCM (3 x 10 mL). The combined organic layers were washed with brine (1 x 10 mL), dried over anhydrous $MgSO_4$ and concentrated *in vacuo*. The crude material was by flash chromatography (0-5% (v/v) MeOH/DCM), to afford the ethynylphenol **S2** as a dark red solid (76 mg, 0.76 mmol, 51 %).

^{1}H NMR (400 MHz, Chloroform-*d*) δ 7.44 – 7.36 (m, 2H, Ar*H*), 6.80 – 6.73 (m, 2H, Ar*H*), 4.84 (s, 1H, Ar-O*H*), 2.99 (s, 1H, C≡C*H*).
^{13}C{^{1}H} NMR (101 MHz, Chloroform-*d*) δ 156.1, 134.0, 115.6, 114.6, 83.6, 75.9.
Spectral data were consistent with the values reported in literature.[6]

N-Benzylprop-2-yn-1-amine (4c)

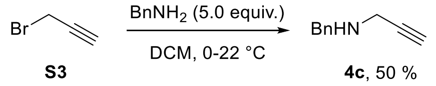

Scheme S3. Synthesis of Benzyl Propargyl amine **S3**.

According to a reported procedure[7], to a flame-dried 250 mL two-necked round-bottom flask, benzylamine (55 mL, 0.50 mol, 5.0 equiv.) and DCM (60 mL) were added. The mixture was cooled to 0 °C. Then, *via* an addition funnel, propargyl bromide (80 wt% solution in toluene, 10.8 mL, 100 mmol, 1.0 equiv.) in DCM (40 mL) was added drop-wise over 1 hour. The reaction mixture was allowed to reach room temperature and stirred for 5 h. The reaction mixture was filtered through a plug of silica and concentrated *in vacuo* to approx. 100 mbar. The mixture was distilled under reduced pressure to give the *N*-benzylprop-2-yn-1-amine **4c** as a colorless oil (7.3 g, 50 mmol, ~90% purity according to ^{1}H NMR (T = 50 – 55 °C, 0.35 mbar). The amine can be also re-purified via column chromatography (10 – 40 % (v/v) EtOAc in pentane).

^{1}H NMR (400 MHz, Chloroform-*d*) δ 7.41 – 7.31 (m, 4H, Ar*H*), 7.31 – 7.24 (m, 1H, ArH), 3.90 (s, 2H, PhCH_2), 3.44 (d, J = 2.4 Hz, 2H, CH_2C≡CH), 2.28 (t, J = 2.4 Hz, 1H, C≡C*H*), 1.49 (s, 1H, N*H*).

^{13}C{^{1}H} NMR (101 MHz, Chloroform-*d*) δ 139.5, 128.52, 128.49, 127.2, 82.2, 71.6, 52.4, 37.4.

Spectral data were consistent with the values reported in literature.[7]

N-Benzylbut-3-yn-1-amine (S5)

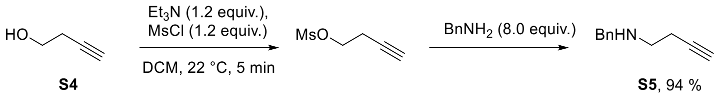

Scheme S4. Synthesis of Benzyl Propargyl amine **S4**.

According to a reported procedure,[8] a flame-dried 50 mL round-bottom flask was charged with but-3-yn-1-ol (0.70 g, 0.76 mL, 10.0 mmol, 1.0 equiv.), mesityl chloride (1.4 g, 0.93 mL, 12.0 mmol, 1.2 equiv.), triethylamine (1.2 g, 1.7 mL, 12 mmol, 1.2 equiv.) and DCM (10 mL). A white precipitate immediately formed. After the mixture was stirred for 5 min, the solvent was evaporated till dryness and benzylamine (8.6 g, 8.7 mL, 80 mmol, 8.0 equiv) was added to the residue. The resulting suspension was heated at 55 °C for 16 h. The reaction mixture was quenched with sat. aq. $NaHCO_3$ (10 mL) and extracted with Et_2O (3×20 mL). The combined organic layers were dried over anhydrous Na_2SO_4, filtered and concentrated *in vacuo*. The crude product was purified by column chromatography (10 – 40 % (v/v) EtOAc in pentane) to afford *N*-benzylbut-3-yn-1-amine (**S5**) as a pale-yellow oil (1.5 g, 9.4 mmol, 94 % yield).

^{1}H NMR (400 MHz, Chloroform-*d*) δ 7.36 – 7.30 (m, 4H, Ar*H*), 7.29 – 7.23 (m, 1H, Ar*H*), 3.83 (s, 2H, PhCH_2N), 2.81 (t, J = 6.6 Hz, 2H, -NCH_2CH$_2$-), 2.42 (td, J = 6.6, 2.6 Hz, 2H, -NCH$_2$CH_2-), 2.00 (t, J = 2.6 Hz, 1H, -CH$_2$C≡C*H*), 1.62 (br. s, 1H, N*H*).

^{13}C{^{1}H} NMR (101 MHz, Chloroform-*d*) δ 140.3, 128.6, 128.2, 127.1, 82.6, 69.7, 53.5, 47.5, 19.7.

Spectral data were consistent with the values reported in literature.[8]

C. Optimization Studies

Table S1. Influence of the ligand

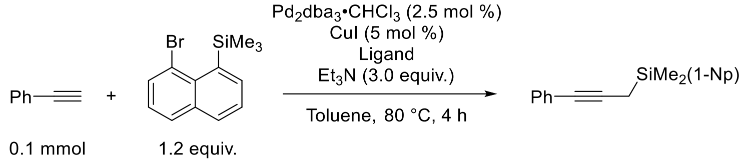

Entry	Ligand (mol %)	Yield (%)[a]
1	SPhos (10)	77
2	BrettPhos (10)	7
3	CyJohnPhos (10)	44
4	DavePhos (10)	92
5	RuPhos (10)	79
6	tBuxPhos (10)	2
7	xPhos (10)	20
8	triphenylphosphine (10)	0
9	tri(o-totyl)phosphine (10)	0
10	tricyclohexylphosphine (10)	2
11	tri(2-furyl)phosphine (10)	0
12	tri-tert-butylphosphine (10)	3
13	XantPhos (5)	0
14	DPE-Phos (5)	0
15	Dppf (5)	0
16	bis(dicyclohexylphosphino)ether (5)	0
17	PTBPF (5)	0
18	Dppb (5)	0
19	Dppe (5)	0
20	Dppp (5)	0

[a]NMR yields determined using trichloroethylene (1.0 equiv.) as internal standard.

Table S2. Control reactions

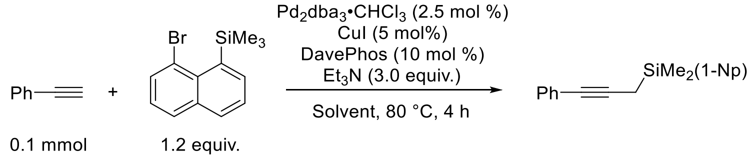

Entry	Control	Yield (%)[a]
1	No Cu	88
2	No Pd	0
3	No ligand	0
4	No base	0
5	Under air	26

[a]NMR yields determined using trichloroethylene (1.0 equiv.) as internal standard.

Table S3. Influence of the solvent and the base

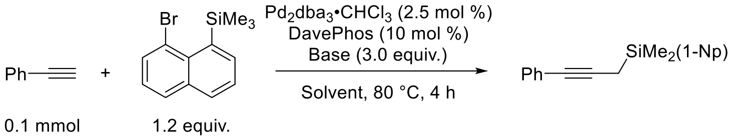

Entry	Solvent	Base (equiv.)	Yield (%)[a]
1	iPrOH	Et_3N (3.0)	41
2	MeTHF	Et_3N (3.0)	21
3	EtOAc	Et_3N (3.0)	82
4	Toluene	Et_3N (3.0)	88
5	Toluene	KOH (3.0)	60
6	Toluene	K_2CO_3 (3.0)	48
7	Toluene	K_3PO_4 (3.0)	62

[a]NMR yields determined using trichloroethylene (1.0 equiv.) as internal standard.

Table S4. Fine tuning of the reaction conditions

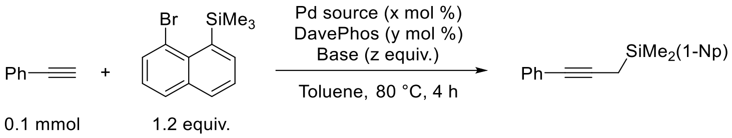

Entry	Pd source (mol %)	Ligand	Base (equiv.)	Scale	Yield (%)[a]
1	Pd_2dba_3•$CHCl_3$(2.5)	DavePhos (10)	Et_3N (3.0)	0.1 mmol	88
2	Pd_2dba_3•$CHCl_3$(2.5)	DavePhos (10)	Et_3N (1.2)	0.1 mmol	69
3	$Pd(OAc)_2$ (5)	DavePhos (10)	Et_3N (1.2)	0.1 mmol	54
4	Pd_2dba_3•$CHCl_3$ (1.25)	DavePhos (5)	Et_3N (3.0)	0.4 mmol	73
5	Pd_2dba_3•$CHCl_3$ (0.625)	DavePhos (2.5)	Et_3N (3.0)	0.4 mmol	72
6	Pd_2dba_3•$CHCl_3$(2.5)	DavePhos (10)	Et_3N (3.0)	0.4 mmol	90

[a]NMR yields determined using trichloroethylene (1.0 equiv.) as internal standard.

Table S5. Influence of the solvent

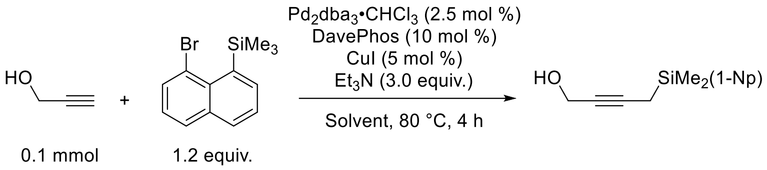

Entry	Solvent	Yield (%)[a]
1	Toluene	15
2	DCE	4
3	MeTHF	11
4	MeCN	2
5	EtOH	3
6	DMF	7
7	EtOAc	3
8[b]	Toluene	12

[a]NMR yields determined using trichloroethylene (1.0 equiv.) as internal standard. [b]Without CuI

Table S6. Influence of the base

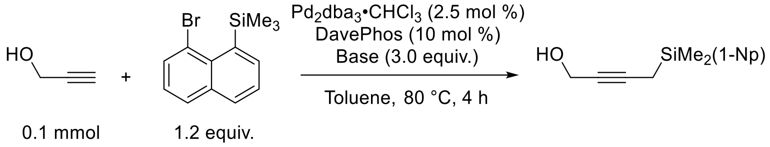

Entry	Base	Yield (%)[a]
1	Et_3N	12
2	Pyridine	0
3	pyrrolidine	0
4	DIPEA	3
5	DBU	0
6	K_2CO_3	10
7	K_3PO_4	25
8	Cs_2CO_3	4

[a]NMR yields determined using trichloroethylene (1.0 equiv.) as internal standard.

Table S7. Influence of the Pd source

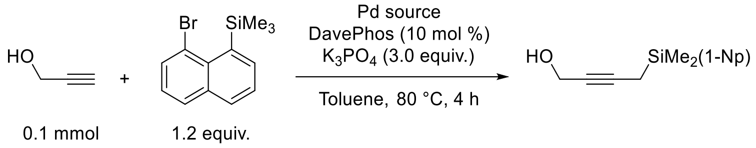

Entry	Pd source (mol %)	Yield (%)[a]
1	Pd_2dba_3•$CHCl_3$ (2.5)	25
2	$PdCl_2$ (5)	0
3	$Pd(OAc)_2$ (5)	21
4	$Pd(PhCN)_2Cl_2$ (5)	13
5	$Pd(COD)Cl_2$ (5)	18
6	$(C_3H_4PdCl)_2$ (2.5)	20
7	Pd_2dba_3•$CHCl_3$ (5)	5
8	Pd_2dba_3•$CHCl_3$[b] (5)	20

[a]NMR yields determined using trichloroethylene (1.0 equiv.) as internal standard. [b]10 mol % of [Pd], 20 mol % of Ligand

Table S8. Influence of the ligand

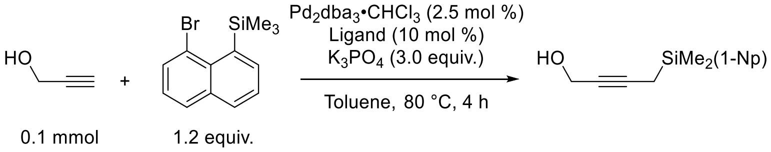

Entry	Ligand (10 mol %)	Yield (%)[a]
1	DavePhos	25
2	SPhos	29
3	CyJohnPhos	18
4	RuPhos	20
5	XPhos	13
6	PhDavePhos	0
7	CPhos	17
8	MePhos	24
9	2-(Dicyclohexylphosphino)-2'-methoxy-1,1'-biphenyl	10

[a]NMR yields determined using trichloroethylene (1.0 equiv.) as internal standard.

Table S9. Fine tuning of the reaction conditions

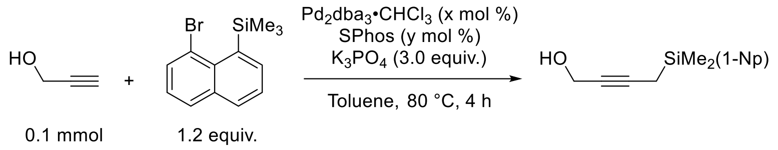

Entry	Pd_2dba_3•$CHCl_3$ loading (mol %)	SPhos Loading (mol %)	Scale (mmol)	Yield (%)[a]
1	2.5	10	0.1	30
2	1.25	5	0.1	22
3	0.625	2.5	0.1	20

[a]NMR yield

Table S10. Influence of the ligand for But-3-yn-1-ol

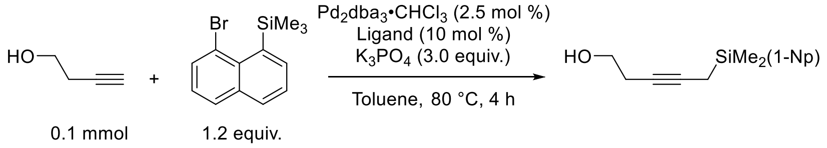

Entry	Ligand (10 mol %)	Yield (%)[a]
1	DavePhos	42
2	SPhos	63
3	CyJohnPhos	59
4	RuPhos	51
5	XPhos	52
6	PhDavePhos	2
7	CPhos	38
8	MePhos	56
9	2-(Dicyclohexylphosphino)-2'-methoxy-1,1'-biphenyl	55

[a]NMR yield

Table S11. Fine tuning of the reaction conditions for But-3-yn-1-ol

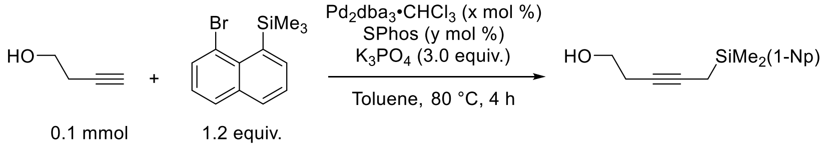

Entry	Pd_2dba_3•$CHCL_3$ loading (mol %)	SPhos loading (mol%)	Scale (mmol)	Yield (%)[a]
1	2.5	10	0.1	63
2	1.25	10	0.1	66
3	1.25	7	0.1	39
4	1.25	5	0.1	66
5	0.625	2.5	0.1	48
6	1.25	5	0.4	54
7	2.5	10	0.4	62

[a]NMR yield

D. Procedures and product characterization data of propargyl silanes

D.1. General Procedure for the migratory Sonogashira reaction with aryl substrates.

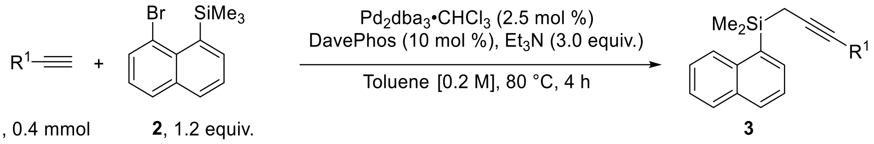

Scheme 5. Migratory Sonogashira reaction for aryl substituted alkynes.

An oven-dried 8 mL microwave tube equipped with a Teflon coated stirring bar was charged with DavePhos (15.7 mg, 40.0 µmol, 10 mol %) and Pd_2dba_3•$CHCl_3$ (10.4 mg, 10.0 µmol, 2.5 mol %) in the glove box. Toluene (1.2 mL) and Et_3N (121 mg, 167 µL, 1.20 mmol, 3.0 equiv) were added and the mixture was stirred at **50 °C for 10 minutes**. Afterwards, a solution of the electrophile (134 mg, 0.480 mmol, 1.2 equiv) and the corresponding alkyne (0.400 mmol) in toluene (0.80 mL) was added. The resulting solution was then stirred at **80 °C for 4 h**. Next, the reaction mixture was allowed to cool down to room temperature and filtered through a plug of silica gel eluting with EtOAc in pentane (10 mL of 50 % (v/v)) and concentrated in vacuo. The crude material was purified by flash column chromatography on silica gel using a Biotage flash chromatography machine to afford the corresponding product.

D.2. Characterization of the aryl propargyl silanes.

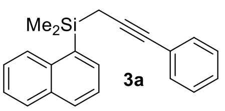

Dimethyl(naphthalen-1-yl)(3-phenylprop-2-yn-1-yl)silane (3a)

Prepared according to the general procedure D1 using (8-bromonaphthalen-1-yl)-trimethylsilane (134 mg, 480 µmol, 1.20 equiv) and ethynylbenzene (40.9 mg, 400 µmol, 43.9 µL, 1.00 equiv). The crude material was purified by column chromatography (0 – 10 % (v/v) DCM in pentane) to give **3a** (98 mg, 0.33 mmol, 82 % yield) as a pale-yellow oil.

R_f (10% DCM/Pentane) = 0.36.

^{1}H NMR (400 MHz, $CDCl_3$) δ 8.17 – 8.09 (m, 1H, Ar*H*), 7.92 – 7.85 (m, 2H, Ar*H*), 7.77 (dd, *J* = 6.8, 1.3 Hz, 1H), 7.54 – 7.45 (m, 3H, Ar*H*), 7.35 – 7.30 (m, 2H, Ar*H*), 7.29 – 7.23 (m, 3H, Ar*H*), 2.17 (s, 2H, Si-CH_2-C≡C), 0.65 (s, 6H, Si(CH_3)$_2$).
^{13}C{^{1}H} NMR (101 MHz, $CDCl_3$) δ 137.0, 135.6, 133.9, 133.6, 131.6, 130.4, 129.4, 128.3, 128.1, 127.3, 126.1, 125.6, 125.2, 124.8, 88.2, 80.7, 8.0, -1.5.
IR (cm^{-1}) 3052 (m), 2959 (m), 2925 (w), 2211 (w), 1724 (w), 1596 (w), 1491 (m), 1403 (w), 1255 (m), 1151 (m).
HRMS (nanochip-ESI/LTQ-Orbitrap) m/z: [M + H]$^+$ Calcd for $C_{21}H_{21}Si^+$ 301.1407; Found 301.1416.

5 mmol scale: A flame-dried 50 mL Schlenk tube equipped with a Teflon coated stirring bar was charged with DavePhos (197 mg, 0.500 mmol, 10 mol %) and Pd_2dba_3•$CHCl_3$ (129 mg, 0.125 mmol, 2.5 mol %) in the glove box. Toluene (15 mL) and Et_3N (1.5 g, 2.1 mL, 15 mmol, 3.0 equiv) were added and the mixture was stirred at **50 °C for 10 minutes**. Afterwards, a solution of the electrophile (1.68 g, 6.00 mmol, 1.2 equiv) and phenylacetylene (0.51 g, 0.55 mL, 5.00 mmol) in toluene (10 mL) was added. The resulting solution was then stirred at **80 °C for 4 h**. Next, the reaction mixture was allowed to cool down to room temperature and filtered through a plug of silica gel eluting with EtOAc in pentane (100 mL of 50 % (v/v)) and concentrated in vacuo. The crude material was purified by flash column chromatography on silica gel using a Biotage flash chromatography machine to afford to afford **3a** (1.21 g, 4.02 mmol, 80% yield) as a pale-yellow oil.

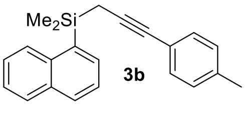

Dimethyl(naphthalen-1-yl)(3-(p-tolyl)prop-2-yn-1-yl)silane (3b)

Prepared according to the general procedure D1 using (8-bromonaphthalen-1-yl)-trimethylsilane (134 mg, 480 µmol, 1.20 equiv) and 1-ethynyl-4-methylbenzene (46.5 mg, 400 µmol, 50.7 µL, 1.00 equiv). The crude material was purified by column chromatography (0 – 10 % (v/v) DCM in pentane) to give **3b** (82 mg, 0.26 mmol, 65 % yield) as a pale-yellow oil.

R_f (10% DCM/Pentane) = 0.41.

^{1}H NMR (400 MHz, $CDCl_3$) 8.18 – 8.13 (m, 1H, Ar*H*), 7.93 – 7.87 (m, 2H, Ar*H*), 7.79 (dd, *J* = 6.8, 1.3 Hz, 1H, Ar*H*), 7.56 – 7.46 (m, 3H, Ar*H*), 7.26 – 7.23 (m, 2H, Ar*H*), 7.12 – 7.06 (m, 2H, Ar*H*), 2.34 (s, 3H, C≡C-Ph-C*H*$_3$), 2.18 (s, 2H, Si-C*H*$_2$-C≡C), 0.66 (s, 6H, Si(C*H*$_3$)$_2$).
$^{13}C\{^1H\}$ NMR (101 MHz, $CDCl_3$) δ 137.2, 137.0, 135.7, 133.9, 133.6, 131.4, 130.4, 129.3, 129.0, 128.1, 126.0, 125.6, 125.2, 121.7, 87.3, 80.7, 21.5, 8.0, -1.5.
IR (cm^{-1}) 3047 (w), 2958 (w), 2208 (w), 1508 (m), 1401 (w), 1253 (m), 1148 (m).
HRMS (Sicrit plasma/LTQ-Orbitrap) m/z: [M + H]$^+$ Calcd for $C_{22}H_{23}Si^+$ 315.1564; Found 315.1559.

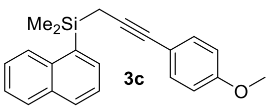

(3-(4-Methoxyphenyl)prop-2-yn-1-yl)dimethyl(naphthalen-1-yl)silane (3c)

Prepared according to the general procedure D1 using (8-bromonaphthalen-1-yl)-trimethylsilane (134 mg, 480 µmol, 1.20 equiv) and 1-ethynyl-4-methoxybenzene (52.9 mg, 400 µmol, 51.9 µL, 1.00 equiv). The crude material was purified by column chromatography (0 – 10 % (v/v) DCM in pentane) to give **3c** (96 mg, 0.29 mmol, 73 % yield) as a yellow oil.

R_f (10% DCM/Pentane) = 0.16.

^{1}H NMR (400 MHz, $CDCl_3$) δ 8.17 – 8.11 (m, 1H, Ar*H*), 7.93 – 7.87 (m, 2H, Ar*H*), 7.78 (dd, *J* = 6.8, 1.3 Hz, 1H, Ar*H*), 7.55 – 7.45 (m, 3H, Ar*H*), 7.29 – 7.25 (m, 2H, Ar*H*), 6.83 – 6.78 (m, 2H, Ar*H*), 3.80 (s, 3H, OC*H*$_3$), 2.15 (s, 2H, Si-C*H*$_2$-C≡C), 0.65 (s, 6H, Si(C*H*$_3$)$_2$).
$^{13}C\{^1H\}$ NMR (101 MHz, $CDCl_3$) δ 158.9, 137.0, 135.7, 133.9, 133.6, 132.9, 130.4, 129.3, 128.1, 126.0, 125.6, 125.2, 117.0, 113.9, 86.3, 80.3, 55.4, 8.0, -1.5.
IR (cm^{-1}) 3050 (w), 3004 (w), 2957 (m), 2835 (w), 2211 (w), 1721 (w), 1606 (m), 1508 (s), 1462 (w), 1290 (m), 1246 (s), 1175 (m).
HRMS (Sicrit plasma/LTQ-Orbitrap) m/z: [M + H]$^+$ Calcd for $C_{22}H_{23}OSi^+$ 331.1513; Found 331.1509.

4-(3-(Dimethyl(naphthalen-1-yl)silyl)prop-1-yn-1-yl)-N,N-dimethylaniline (3d)

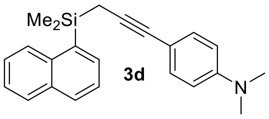

Prepared according to the general procedure D1 using (8-bromonaphthalen-1-yl)-trimethylsilane (134 mg, 480 µmol, 1.20 equiv) and 4-ethynyl-N,N-dimethylaniline (58.1 mg, 400 µmol, 1.00 equiv). The crude material was purified by column chromatography (0 – 5 % (v/v) EtOAc in pentane) to give **3d** (108 mg, 0.310 mmol, 79 % yield) as a red oil.

R_f (50% DCM/Pentane) = 0.60.

^{1}H NMR (400 MHz, $CDCl_3$) δ 8.15 (ddd, *J* = 7.2, 2.1, 0.8 Hz, 1H, Ar*H*), 7.92 – 7.86 (m, 2H, Ar*H*), 7.78 (dd, *J* = 6.8, 1.3 Hz, 1H, Ar*H*), 7.55 – 7.45 (m, 3H, Ar*H*), 7.25 – 7.21 (m, 2H, Ar*H*), 6.64 – 6.59 (m, 2H, Ar*H*), 2.95 (s, 6H, Ar-N-(C*H*$_3$)$_2$), 2.15 (s, 2H, Si-C*H*$_2$-C≡C), 0.64 (s, 6H, Si(C*H*$_3$)$_2$).
$^{13}C\{^1H\}$ NMR (101 MHz, $CDCl_3$) δ 149.7, 137.0, 136.0, 133.9, 133.5, 132.5, 130.3, 129.3, 128.2, 126.0, 125.6, 125.2, 112.2, 112.1, 85.1, 81.1, 40.5, 8.0, -1.6.
IR (cm^{-1}) 3041 (w), 2894 (w), 2801 (w), 1609 (s), 1521 (s), 1445 (m), 1356 (m), 1253 (m), 1190 (m).
HRMS (Sicrit plasma/LTQ-Orbitrap) m/z: [M + H]^0 Calcd for $C_{23}H_{26}NSi^+$ 344.1829; Found 344.1821.

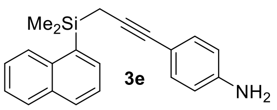

4-(3-(Dimethyl(naphthalen-1-yl)silyl)prop-1-yn-1-yl)aniline (3e)

Prepared according to the general procedure D1 using (8-bromonaphthalen-1-yl)-trimethylsilane (134 mg, 480 µmol, 1.20 equiv) and 4-ethynylaniline (46.9 mg, 400 µmol, 1.00 equiv). The crude material was purified by column chromatography (0 – 50 % (v/v) EtOAc in pentane) to give **3e** (110 mg, 0.350 mmol, 87 % yield) as a black oil.

R_f (50% DCM/Pentane) = 0.16.

^{1}H NMR (400 MHz, $CDCl_3$) δ 8.16 – 8.09 (m, 1H, Ar*H*), 7.91 – 7.84 (m, 2H, Ar*H*), 7.77 (dd, *J* = 6.8, 1.3 Hz, 1H, Ar*H*), 7.54 – 7.44 (m, 3H, Ar*H*), 7.18 – 7.10 (m, 2H, Ar*H*), 6.59 – 6.53 (m, 2H, Ar*H*), 3.72 (s, 2H, Ph-N*H*$_2$), 2.14 (s, 2H, Si-C*H*$_2$-C≡C), 0.64 (s, 6H, Si(C*H*$_3$)$_2$).
$^{13}C\{^1H\}$ NMR (101 MHz, $CDCl_3$) δ 145.8, 137.0, 135.9, 133.9, 133.5, 132.8, 130.3, 129.3, 128.2, 126.0, 125.6, 125.2, 114.9, 114.5, 85.3, 80.8, 7.9, -1.6.
IR (cm^{-1}) 3467 (m), 3383 (m), 3053 (m), 1622 (s), 1511 (s), 1292 (m), 1148 (m).
HRMS (Sicrit plasma/LTQ-Orbitrap) m/z: [M + H]$^+$ Calcd for $C_{21}H_{22}NSi^+$ 316.1516; Found 316.1507.

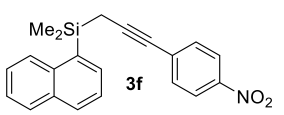

Dimethyl(naphthalen-1-yl)(3-(4-nitrophenyl)prop-2-yn-1-yl)silane (3f)

Prepared according to the general procedure D1 using (8-bromonaphthalen-1-yl)-trimethylsilane (134 mg, 480 μmol, 1.20 equiv) and 1-ethynyl-4-nitrobenzene (58.9 mg, 400 μmol, 1.00 equiv). The crude material was purified by column chromatography (20 – 40 % (v/v) DCM in pentane) to give **3f** (65 mg, 0.19 mmol, 47 % yield) as a pale-yellow oil.

R_f (50% DCM/Pentane) = 0.71.

^{1}H NMR (400 MHz, $CDCl_3$) δ 8.14 – 8.06 (m, 3H, Ar*H*), 7.94 – 7.87 (m, 2H, Ar*H*), 7.76 (dd, *J* = 6.8, 1.3 Hz, 1H, Ar*H*), 7.55 – 7.45 (m, 3H, Ar*H*), 7.41 – 7.35 (m, 2H, Ar*H*), 2.22 (s, 2H, Si-CH_2-C≡C), 0.66 (s, 6H, Si(CH_3)$_2$).
^{13}C{^{1}H} NMR (101 MHz, $CDCl_3$) δ 146.4, 136.93, 135.0, 134.0, 133.6, 132.1, 131.9, 130.7, 129.5, 127.9, 126.2, 125.7, 125.2, 123.6, 95.3, 79.5, 8.6, -1.4.
IR (cm^{-1}) 3050 (w), 2955 (w), 2209 (m), 1593 (m), 1516 (s), 1342 (s), 1109 (w).
HRMS (Sicrit plasma/LTQ-Orbitrap) m/z: [M + H]$^+$ Calcd for $C_{21}H_{20}NO_2Si^+$ 346.1258; Found 346.1253.

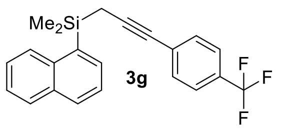

Dimethyl(naphthalen-1-yl)(3-(4-(trifluoromethyl)phenyl)prop-2-yn-1-yl)silane (3g)

Prepared according to the general procedure D1 using (8-bromonaphthalen-1-yl)-trimethylsilane (134 mg, 480 μmol, 1.20 equiv) and 1-ethynyl-4-(trifluoromethyl)benzene (68.1 mg, 400 μmol, 1.00 equiv). The crude material was purified by column chromatography (100 % pentane) to give **3g** (131 mg, 0.360 mmol, 89 % yield) as an orange oil.

R_f (Pentane) = 0.29.

^{1}H NMR (400 MHz, $CDCl_3$) δ 8.14 – 8.09 (m, 1H, Ar*H*), 7.93 – 7.87 (m, 2H, Ar*H*), 7.77 (dd, *J* = 6.9, 1.3 Hz, 1H, Ar*H*), 7.55 – 7.45 (m, 5H, Ar*H*), 7.42 – 7.35 (m, 2H, Ar*H*), 2.19 (s, 2H, C≡C-Ar-(CH_3)$_3$), 0.66 (s, 6H, Si(CH_3)$_2$).
^{13}C{^{1}H} NMR (101 MHz, $CDCl_3$) δ 137.0, 135.3, 134.0, 133.6, 131.7, 130.6, 128.4, 129.0 (q, $J_{C\text{-}F}$ = 32.5 Hz), 128.6 (q, $J_{C\text{-}F}$ = 1.5 Hz), 128.0, 126.1, 125.7, 125.22, 125.20 (q, $J_{C\text{-}F}$ = 3.7 Hz), 124.2 (q, $J_{C\text{-}F}$ = 271.9 Hz), 91.5, 79.6, 8.2, -1.5.
^{19}F NMR (376 MHz, $CDCl_3$) δ -62.7.
IR (cm^{-1}) 3056 (w), 2959 (w), 2211 (w), 1617 (w), 1509 (w), 1404 (w), 1325 (s), 1256 (w), 1166 (m), 1126 (s).
HRMS (Sicrit plasma/LTQ-Orbitrap) m/z: [M + H]$^+$ Calcd for $C_{22}H_{20}F_3Si^+$ 369.1281; Found 369.1282.

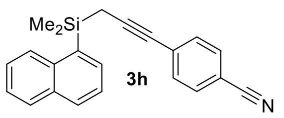

4-(3-(Dimethyl(naphthalen-1-yl)silyl)prop-1-yn-1-yl)benzonitrile (3h)

Prepared according to the general procedure D1 using (8-bromonaphthalen-1-yl)-trimethylsilane (134 mg, 480 μmol, 1.20 equiv) and 4-ethynylbenzonitrile (50.9 mg, 400 μmol, 1.00 equiv). The crude material was purified by column chromatography (0 – 5 % (v/v) EtOAc in pentane) to give **3h** (56 mg, 0.17 mmol, 43 % yield) as a pale-yellow solid.

R_f (50% DCM/Pentane) = 0.55.

^{1}H NMR (400 MHz, $CDCl_3$) δ 8.12 – 8.06 (m, 1H, Ar*H*), 7.94 – 7.86 (m, 2H, Ar*H*), 7.75 (dd, *J* = 6.8, 1.3 Hz, 1H, Ar*H*), 7.55 – 7.44 (m, 5H, Ar*H*), 7.37 – 7.31 (m, 2H, Ar*H*), 2.20 (s, 2H, Si-CH_2-C≡C), 0.65 (s, 6H, Si(CH_3)$_2$).
^{13}C{^{1}H} NMR (101 MHz, $CDCl_3$) δ 136.9, 135.0, 134.0, 133.6, 132.0, 132.0, 130.6, 129.8, 129.4, 127.9, 126.1, 125.7, 125.2, 118.9, 110.5, 94.1, 79.6, 8.4, -1.4.
IR (cm^{-1}) 3065 (m), 2956 (m), 2227 (m), 2210 (s), 1603 (m), 1504 (m), 1402 (m), 1267 (m), 1148 (m).
HRMS (Sicrit plasma/LTQ-Orbitrap) m/z: [M + H]$^+$ Calcd for $C_{22}H_{20}NSi^+$ 326.1360; Found 326.1359.

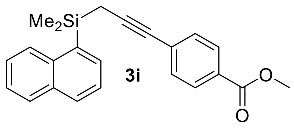

Methyl 4-(3-(dimethyl(naphthalen-1-yl)silyl)prop-1-yn-1-yl)benzoate (3i)

Prepared according to the general procedure D1 using (8-bromonaphthalen-1-yl)-trimethylsilane (134 mg, 480 μmol, 1.20 equiv) and methyl 4-ethynylbenzoate (64.1 mg, 400 μmol, 1.00 equiv). The crude material was purified by column chromatography (20 – 70 % (v/v) DCM in pentane) to give **3i** (106 mg, 0.300 mmol, 74 % yield) as a yellow solid.

Rf (50% DCM/Pentane) = 0.48.

^{1}H NMR (400 MHz, $CDCl_3$) δ 8.16 – 8.08 (m, 1H, Ar*H*), 7.96 – 7.86 (m, 4H, Ar*H*), 7.76 (dd, *J* = 6.8, 1.3 Hz, 1H, Ar*H*), 7.55 – 7.44 (m, 3H, Ar*H*), 7.37 – 7.32 (m, 2H, Ar*H*), 3.91 (s, 3H, Ph-COOCH_3), 2.20 (s, 2H, Si-CH_2-C≡C), 0.66 (s, 6H, Si(CH_3)$_2$).
$^{13}C\{^1H\}$ NMR (101 MHz, $CDCl_3$) δ 166.9, 137.0, 135.3, 134.0, 133.6, 131.4, 130.5, 129.7, 129.5, 129.4, 128.6, 128.0, 126.1, 125.7, 125.2, 92.2, 80.3, 52.3, 8.3, -1.5.
IR (cm^{-1}) 3059 (w), 2952 (w), 2255 (w), 2210 (w), 1721 (m), 1278 (m), 1111 (w).
HRMS (Sicrit plasma/LTQ-Orbitrap) m/z: $[M + H]^+$ Calcd for $C_{23}H_{23}O_2Si^+$ 359.1462; Found 359.1458.

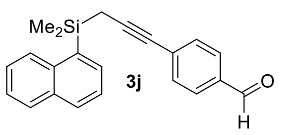

4-(3-(Dimethyl(naphthalen-1-yl)silyl)prop-1-yn-1-yl)benzaldehyde (3j)

Prepared according to the general procedure D1 using (8-bromonaphthalen-1-yl)-trimethylsilane (134 mg, 480 μmol, 1.20 equiv) and 4-ethynylbenzaldehyde (52.1 mg, 400 μmol, 1.00 equiv). The crude material was purified by column chromatography (10 – 60 % (v/v) DCM in pentane) to give **3j** (67 mg, 0.20 mmol, 51 % yield) as a pale-yellow oil.

Rf (50% DCM/Pentane) = 0.39.

^{1}H NMR (400 MHz, $CDCl_3$) δ 9.97 (s, 1H, Ar-C*H*O), 8.15 – 8.07 (m, 1H, Ar*H*), 7.95 – 7.87 (m, 2H, Ar*H*), 7.82 – 7.74 (m, 3H, Ar*H*), 7.53 – 7.40 (m, 5H, Ar*H*), 2.22 (s, 2H, Si-CH_2-C≡C), 0.66 (s, 6H, Si(CH_3)$_2$).
$^{13}C\{^1H\}$ NMR (101 MHz, $CDCl_3$) δ 191.7, 136.9, 135.2, 134.8, 134.0, 133.6, 132.0, 131.3, 130.6, 129.6, 129.4, 128.0, 126.1, 125.7, 125.2, 93.7, 80.3, 8.5, -1.4.
IR (cm^{-1}) 3053 (w), 2957 (m), 2924 (m), 2852 (w), 2210 (w), 1703 (m), 1601 (m), 1506 (m), 1393 (w), 1254 (m), 1220 (m), 1152 (m).
HRMS (Sicrit plasma/LTQ-Orbitrap) m/z: $[M + H]^+$ Calcd for $C_{22}H_{21}OSi^+$ 329.1356; Found 329.1349.

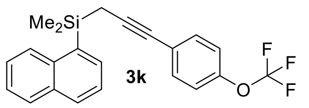

Dimethyl(naphthalen-1-yl)(3-(4-(trifluoromethoxy)phenyl)prop-2-yn-1-yl)silane (3k)

Prepared according to the general procedure D1 using (8-bromonaphthalen-1-yl)-trimethylsilane (134 mg, 480 μmol, 1.20 equiv) and 1-ethynyl-4-(trifluoromethoxy)benzene (74.5 mg, 400 μmol, 61.3 μL, 1.00 equiv). The crude material was purified by column chromatography (0 – 10 % (v/v) DCM in pentane) to give **3k** (130 mg, 0.340 mmol, 85 % yield) as an orange oil.

Rf (10% DCM/Pentane) = 0.53.

^{1}H NMR (400 MHz, $CDCl_3$) δ 8.14 – 8.08 (m, 1H, Ar*H*), 7.93 – 7.87 (m, 2H, Ar*H*), 7.76 (dd, *J* = 6.9, 1.3 Hz, 1H, Ar*H*), 7.55 – 7.44 (m, 3H, Ar*H*), 7.34 – 7.29 (m, 2H, Ar*H*), 7.13 – 7.07 (m, 2H, Ar*H*), 2.16 (s, 2H, Si-CH_2-C≡C), 0.65 (s, 6H, Si(CH_3)$_2$).
$^{13}C\{^1H\}$ NMR (101 MHz, $CDCl_3$) δ 148.2, 137.0, 135.4, 134.0, 133.6, 132.9, 130.5, 129.4, 128.0, 126.1, 125.7, 125.2, 123.6, 120.9, 120.6 (q , J_{C-F} = 257.1 Hz), 89.4, 79.3, 8.1, -1.5.
^{19}F NMR (376 MHz, $CDCl_3$) δ -57.8.
IR (cm^{-1}) 3054 (w), 2964 (w), 2214 (w), 1506 (m), 1256 (s), 1224 (s), 1206 (s), 1166 (s).
HRMS (Sicrit plasma/LTQ-Orbitrap) m/z: $[M + H]^+$ Calcd for $C_{22}H_{20}F_3OSi^+$ 385.1230; Found 385.1227.

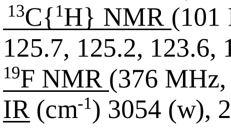

Dimethyl(naphthalen-1-yl)(3-(o-tolyl)prop-2-yn-1-yl)silane (3l)

Prepared according to the general procedure D1 using (8-bromonaphthalen-1-yl)-trimethylsilane (134 mg, 480 μmol, 1.20 equiv) and 1-ethynyl-2-methylbenzene (46.5 mg, 400 μmol, 50.4 μL, 1.00 equiv). The crude material was purified by column chromatography (0 – 10 % (v/v) DCM in pentane) to give **3l** (99 mg, 0.31 mmol, 79 % yield) as a pale-yellow oil.

Rf (10% DCM/Pentane) = 0.41.

^{1}H NMR (400 MHz, $CDCl_3$) δ 8.16 – 8.10 (m, 1H, Ar*H*), 7.93 – 7.86 (m, 2H, Ar*H*), 7.79 (dd, *J* = 6.8, 1.3 Hz, 1H, Ar*H*), 7.56 – 7.44 (m, 3H, Ar*H*), 7.33 (dd, *J* = 7.2, 1.3 Hz, 1H, Ar*H*), 7.17 – 7.06 (m, 3H, Ar*H*), 2.33 (s, 3H, Ar-CH_3), 2.24 (s, 2H, Si-CH_2-C≡C), 0.66 (s, 6H, Si(CH_3)$_2$).
$^{13}C\{^1H\}$ NMR (101 MHz, $CDCl_3$) δ 139.9, 137.0, 135.6, 134.0, 133.6, 132.0, 130.4, 129.37, 129.36, 128.0, 127.5, 126.1, 125.6, 125.5, 125.2, 124.6, 92.0, 79.4, 21.0, 8.3, -1.5.
IR (cm^{-1}) 3056 (m), 2957 (m), 2210 (w), 1485 (m), 1456 (w), 1254 (m), 1148 (m).
HRMS (Sicrit plasma/LTQ-Orbitrap) m/z: $[M + H]^+$ Calcd for $C_{22}H_{23}Si^+$ 315.1564; Found 315.1557.

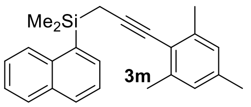

(3-Mesitylprop-2-yn-1-yl)dimethyl(naphthalen-1-yl)silane (3m)

Prepared according to the general procedure D1 using (8-bromonaphthalen-1-yl)-trimethylsilane (134 mg, 480 µmol, 1.20 equiv) and 2-ethynyl-1,3,5-trimethylbenzene (57.7 mg, 400 µmol, 62.6 µL, 1.00 equiv). The crude material was purified by column chromatography (0 – 10 % (v/v) DCM in pentane) to give **3m** (108 mg, 0.310 mmol, 79 % yield) as a pale-yellow oil.

R_f (10% DCM/Pentane) = 0.45.

^{1}H NMR (400 MHz, $CDCl_3$) δ 8.15 – 8.09 (m, 1H, Ar*H*), 7.93 – 7.86 (m, 2H, Ar*H*), 7.80 (dd, *J* = 6.8, 1.3 Hz, 1H, Ar*H*), 7.56 – 7.43 (m, 3H, Ar*H*), 6.84 – 6.81 (m, 2H, Ar*H*), 2.31 (s, 6H, Ar-$(CH_3)_2$), 2.29 (s, 2H, Si-CH_2-C≡C), 2.26 (s, 3H, Ar-CH_3), 0.65 (s, 6H, Si$(CH_3)_2$).
^{13}C{^{1}H} NMR (101 MHz, $CDCl_3$) δ 140.0, 137.0, 136.4, 135.7, 134.0, 133.6, 130.4, 129.4, 128.0, 127.5, 126.0, 125.6, 125.2, 121.5, 95.3, 78.1, 21.33, 21.26, 8.4, -1.5.
IR (cm^{-1}) 3040 (w), 2953 (m), 2914 (m), 2210 (w), 1611 (w), 1476 (w), 1253 (m), 1148 (m).
HRMS (Sicrit plasma/LTQ-Orbitrap) m/z: $[M + H]^+$ Calcd for $C_{24}H_{27}Si^+$ 343.1877; Found 343.1870.

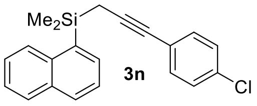

(3-(4-Chlorophenyl)prop-2-yn-1-yl)dimethyl(naphthalen-1-yl)silane (3n)

Prepared according to the general procedure D1 using (8-bromonaphthalen-1-yl)-trimethylsilane (134 mg, 480 µmol, 1.20 equiv) and 1-chloro-4-ethynylbenzene (54.6 mg, 400 µmol, 1.00 equiv). The crude material was purified by column chromatography (0 – 10 % (v/v) DCM in pentane) to give **3n** (72 mg, 0.22 mmol, 54 % yield) as a pale-yellow oil.

R_f (10% DCM/Pentane) = 0.47.

^{1}H NMR (400 MHz, $CDCl_3$) δ 8.15 – 8.09 (m, 1H, Ar*H*), 7.93 – 7.87 (m, 2H, Ar*H*), 7.77 (dd, *J* = 6.8, 1.3 Hz, 1H, Ar*H*), 7.55 – 7.45 (m, 3H, Ar*H*), 7.23 (s, 4H, Ar*H*), 2.16 (s, 2H, Si-CH_2-C≡C), 0.65 (s, 6H, Si$(CH_3)_2$).
^{13}C{^{1}H} NMR (101 MHz, $CDCl_3$) δ 137.0, 135.4, 134.0, 133.6, 133.1, 132.8, 130.5, 129.4, 128.6, 128.0, 126.1, 125.7, 125.2, 123.3, 89.4, 79.6, 8.1, -1.5.
IR (cm^{-1}) 3056 (m), 2961 (m), 2921 (w), 2900 (w), 2255 (w), 2212 (w), 1725 (w), 1490 (m), 1395 (w), 1254 (m), 1147 (m), 1091 (m).
HRMS (Sicrit plasma/LTQ-Orbitrap) m/z: $[M + H]^+$ Calcd for $C_{21}H_{20}ClSi^+$ 335.1017; Found 335.1013.

(3-(4-Fluorophenyl)prop-2-yn-1-yl)dimethyl(naphthalen-1-yl)silane (3o)

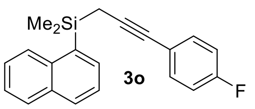

Prepared according to the general procedure D1 using (8-bromonaphthalen-1-yl)-trimethylsilane (134 mg, 480 µmol, 1.20 equiv) and 1-fluoro-4-ethynylbenzene (48.0 mg, 400 µmol, 1.00 equiv). The crude material was purified by column chromatography (0 – 10 % (v/v) DCM in pentane) to give **3o** (91 mg, 0.29 mmol, 71 % yield) as a pale-yellow oil.

R_f (10% DCM/Pentane) = 0.44.

^{1}H NMR (400 MHz, $CDCl_3$) δ 8.14 – 8.09 (m, 1H, Ar*H*), 7.93 – 7.86 (m, 2H, Ar*H*), 7.77 (dd, *J* = 6.9, 1.3 Hz, 1H, Ar*H*), 7.55 – 7.45 (m, 3H, Ar*H*), 7.31 – 7.26 (m, 2H, Ar*H*), 6.98 – 6.91 (m, 2H, Ar*H*), 2.15 (s, 2H, Si-CH_2-C≡C), 0.65 (s, 6H, Si$(CH_3)_2$).
^{13}C{^{1}H} NMR (101 MHz, $CDCl_3$) δ 162.0 (d, *J* = 247.8 Hz), 137.0, 135.5, 133.9, 133.6, 133.3 (d, *J* = 8.1 Hz), 130.5, 129.4, 128.1, 126.1, 125.6, 125.2, 120.8 (d, *J* = 3.6 Hz), 115.4 (d, *J* = 21.9 Hz), 87.8, 79.5, 8.0, -1.5.
^{19}F NMR (376 MHz, $CDCl_3$) δ -112.9.
IR (cm^{-1}) 3056 (w), 2963 (w), 2213 (w), 1728 (w), 1653 (w), 1602 (w), 1506 (s), 1397 (w), 1225 (m), 1152 (w).
HRMS (Sicrit plasma/LTQ-Orbitrap) m/z: $[M + H]^+$ Calcd for $C_{21}H_{20}FSi^+$ 319.1313; Found 319.1312.

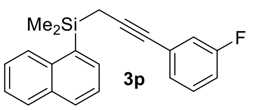

(3-(3-Fluorophenyl)prop-2-yn-1-yl)dimethyl(naphthalen-1-yl)silane (3p)

Prepared according to the general procedure D1 using (8-bromonaphthalen-1-yl)-trimethylsilane (134 mg, 480 µmol, 1.20 equiv) and 1-ethynyl-3-fluorobenzene (48.0 mg, 400 µmol, 46.2 µL, 1.00 equiv). The crude material was purified by column chromatography (0 – 10 % (v/v) DCM in pentane) to give **3p** (101 mg, 0.320 mmol, 79 % yield) as a pale-yellow oil.

R$_f$ (10% DCM/Pentane) = 0.47.

^{1}H NMR (400 MHz, $CDCl_3$) δ 8.15 – 8.07 (m, 1H, Ar*H*), 7.94 – 7.86 (m, 2H, Ar*H*), 7.77 (dd, *J* = 6.8, 1.3 Hz, 1H, Ar*H*), 7.56 – 7.46 (m, 3H, Ar*H*), 7.21 (td, *J* = 8.0, 6.0 Hz, 1H, Ar*H*), 7.09 (dt, *J* = 7.8, 1.3 Hz, 1H, Ar*H*), 7.03 – 6.91 (m, 2H, Ar*H*), 2.17 (s, 2H, Si-CH_2-C≡C), 0.65 (s, 6H, Si(CH_3)$_2$).
^{13}C{^{1}H} NMR (101 MHz, $CDCl_3$) δ 162.5 (d, J_{C-F} = 245.7 Hz), 137.0, 135.4, 134.0, 133.6, 130.5, 129.8 (d, J_{C-F} = 8.8 Hz), 129.4, 128.0, 127.4 (d, J_{C-F} = 2.7 Hz), 126.7 (d, J_{C-F} = 9.5 Hz), 126.1, 125.7, 125.2, 118.3 (d, J_{C-F} = 22.4 Hz), 114.6 (d, J_{C-F} = 21.2 Hz), 89.6, 79.6 (d, J_{C-F} = 3.3 Hz), 8.1, -1.5.
^{19}F NMR (376 MHz, $CDCl_3$) δ -113.6.
IR (cm^{-1}) 3063 (w), 2959 (w), 2222 (m), 1609 (m), 1579 (m), 1486 (m), 1433 (w), 1259 (m), 1145 (m).
HRMS (Sicrit plasma/LTQ-Orbitrap) m/z: [M + H]$^+$ Calcd for $C_{21}H_{20}FSi^+$ 319.1313; Found 319.1308.

(3-(6-Fluorocyclohexa-1,3-dien-1-yl)prop-2-yn-1-yl)dimethyl(naphthalen-1-yl)silane (3q)

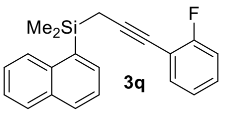

Prepared according to the general procedure D1 using (8-bromonaphthalen-1-yl)-trimethylsilane (134 mg, 480 μmol, 1.20 equiv) and 1-ethynyl-2-fluorobenzene (48.0 mg, 400 μmol, 45.3 μL, 1.00 equiv). The crude material was purified by column chromatography (0 – 10 % (v/v) DCM in pentane) to give **3q** (100 mg, 0.310 mmol, 79 % yield) as a pale-yellow oil.

R$_f$ (10% DCM/Pentane) = 0.41.

^{1}H NMR (400 MHz, $CDCl_3$) δ 8.14 (ddd, *J* = 7.3, 2.2, 0.9 Hz, 1H, Ar*H*), 7.93 – 7.86 (m, 2H, Ar*H*), 7.78 (dd, *J* = 6.8, 1.3 Hz, 1H, Ar*H*), 7.56 – 7.45 (m, 3H, Ar*H*), 7.35 – 7.30 (m, 1H, Ar*H*), 7.22 (dddd, *J* = 7.9, 7.0, 5.3, 1.8 Hz, 1H, Ar*H*), 7.08 – 7.01 (m, 2H, Ar*H*), 2.23 (s, 2H, Si-CH_2-C≡C), 0.67 (s, 6H, Si(CH_3)$_2$).
^{13}C{^{1}H} NMR (101 MHz, $CDCl_3$) δ 163.0 (d, J_{C-F} = 249.6 Hz), 137.0, 135.5, 134.0, 133.57 (d, J_{C-F} = 3.0 Hz), 133.57, 130.5, 129.4, 128.8 (d, J_{C-F} = 7.8 Hz), 128.1, 126.1, 125.6, 125.2, 123.9 (d, J_{C-F} = 3.7 Hz), 115.4 (d, J_{C-F} = 21.1 Hz), 113.2 (d, J_{C-F} = 16.0 Hz), 93.9 (d, J_{C-F} = 3.3 Hz), 73.9, 8.3, -1.6.
^{19}F NMR (376 MHz, $CDCl_3$) δ -111.1.
IR (cm^{-1}) 3058 (m), 2959 (m), 2921 (w), 2895 (w), 2221 (m), 1492 (m), 1451 (m), 1256 (s), 1217 (m), 1147 (m).
HRMS (Sicrit plasma/LTQ-Orbitrap) m/z: [M + H]$^+$ Calcd for $C_{21}H_{20}FSi^+$ 319.1313; Found 319.1306.

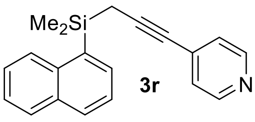

4-(3-(Dimethyl(naphthalen-1-yl)silyl)prop-1-yn-1-yl)pyridine (3r)

Prepared according to the general procedure D1 using (8-bromonaphthalen-1-yl)-trimethylsilane (134 mg, 480 μmol, 1.20 equiv) and 4-ethynylpyridine (41.2 mg, 400 μmol, 1.00 equiv). The crude material was purified by column chromatography (50 – 100 % (v/v) DCM in pentane) to give **3r** (46 mg, 0.15 mmol, 38 % yield) as a black oil.

R$_f$ (100% DCM) = 0.09.

^{1}H NMR (400 MHz, $CDCl_3$) δ 8.51 – 8.45 (m, 2H, Ar*H*), 8.12 – 8.07 (m, 1H, Ar*H*), 7.94 – 7.87 (m, 2H, Ar*H*), 7.75 (dd, *J* = 6.8, 1.3 Hz, 1H, Ar*H*), 7.55 – 7.45 (m, 3H, Ar*H*), 7.16 – 7.12 (m, 2H, Ar*H*), 2.20 (s, 2H, Si-CH_2-C≡C), 0.65 (s, 6H, Si(CH_3)$_2$).
^{13}C{^{1}H} NMR (101 MHz, $CDCl_3$) δ 149.7, 136.9, 135.0, 134.0, 133.6, 133.0, 130.6, 129.4, 127.9, 126.1, 125.8, 125.7, 125.2, 94.4, 78.6, 8.4, -1.5.
IR (cm^{-1}) 3055 (w), 2958 (w), 2925 (w), 2215 (m), 1592 (s), 1405 (w), 1255 (m), 1148 (m).
HRMS (ESI/QTOF) m/z: [M + H]$^+$ Calcd for $C_{20}H_{20}NSi^+$ 302.1360; Found 302.1361.

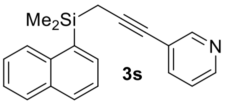

3-(3-(Dimethyl(naphthalen-1-yl)silyl)prop-1-yn-1-yl)pyridine (3s)

Prepared according to the general procedure D1 using (8-bromonaphthalen-1-yl)-trimethylsilane (134 mg, 480 μmol, 1.20 equiv) and 3-ethynylpyridine (41.2 mg, 400 μmol, 1.00 equiv). The crude material was purified by column chromatography (50 – 100 % (v/v) DCM in pentane) to give **3s** (72 mg, 0.24 mmol, 60 % yield) as a pale-yellow oil.

R$_f$ (100% DCM) = 0.19.

^{1}H NMR (400 MHz, $CDCl_3$) δ 8.55 – 8.50 (m, 1H, Ar*H*), 8.45 (dd, *J* = 4.9, 1.7 Hz, 1H, Ar*H*), 8.11 (ddt, *J* = 7.3, 2.8, 0.9 Hz, 1H, Ar*H*), 7.90 (ddd, *J* = 8.8, 6.0, 1.9 Hz, 2H, Ar*H*), 7.76 (dd, *J* = 6.9, 1.3 Hz, 1H, Ar*H*), 7.56 (dt, *J* = 8.0, 2.0 Hz, 1H, Ar*H*), 7.53 – 7.45 (m, 3H, Ar*H*), 7.17 (ddd, *J* = 7.9, 4.9, 0.9 Hz, 1H, Ar*H*), 2.19 (s, 2H, Si-CH_2-C≡C), 0.66 (s, 6H, Si(CH_3)$_2$).
^{13}C{^{1}H} NMR (101 MHz, $CDCl_3$) δ 152.4, 147.7, 138.4, 136.9, 135.2, 134.0, 133.6, 130.6, 129.4, 127.9, 126.1, 125.7, 125.2, 123.0, 121.8, 92.1, 77.4, 8.2, -1.5.
IR (cm^{-1}) 3052 (m), 2957 (m), 2214 (m), 1723 (w), 1505 (w), 1476 (w), 1407 (m), 1255 (m), 1152 (m).

HRMS (ESI/QTOF) m/z: $[M + H]^+$ Calcd for $C_{20}H_{20}NSi^+$ 302.1360; Found 302.1356.

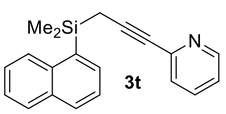

3t

2-(3-(Dimethyl(naphthalen-1-yl)silyl)prop-1-yn-1-yl)pyridine (3t)

Prepared according to the general procedure D1 using (8-bromonaphthalen-1-yl)-trimethylsilane (134 mg, 480 µmol, 1.20 equiv) and 2-ethynylpyridine (41.2 mg, 400 µmol, 40.4 µL, 1.00 equiv). The crude material was purified by column chromatography (10 – 50 % (v/v) EtOAc in pentane) and then (0-5% (v/v)MeOH in DCM) to give **3t** (32 mg, 0.11 mmol, 27 % yield) as a black oil.

R_f (100% DCM) = 0.28.

^{1}H NMR (400 MHz, $CDCl_3$) δ 8.53 (ddd, *J* = 4.9, 1.8, 1.0 Hz, 1H, Ar*H*), 8.14 – 8.08 (m, 1H, Ar*H*), 7.92 – 7.84 (m, 2H, Ar*H*), 7.77 (dd, *J* = 6.8, 1.3 Hz, 1H, Ar*H*), 7.57 (td, *J* = 7.8, 1.9 Hz, 1H, Ar*H*), 7.54 – 7.45 (m, 3H, Ar*H*), 7.25 (dd, *J* = 7.8, 1.1 Hz, 1H, Ar*H*), 7.14 (ddd, *J* = 7.6, 4.9, 1.2 Hz, 1H, Ar*H*), 2.22 (s, 2H, Si-CH_2-C≡C), 0.67 (s, 6H, Si(CH_3)$_2$).
^{13}C{^{1}H} NMR (101 MHz, $CDCl_3$) δ 149.9, 144.6, 136.9, 136.0, 135.3, 133.9, 133.6, 130.5, 129.4, 128.0, 126.8, 126.1, 125.6, 125.2, 122.0, 89.4, 80.6, 8.1, -1.5.
IR (cm^{-1}) 3051 (w), 2957 (w), 2925 (w), 2218 (m), 1582 (m), 1464 (m), 1427 (m), 1256 (m), 1148 (m)
HRMS (ESI/QTOF) m/z: $[M + H]^+$ Calcd for $C_{20}H_{20}NSi^+$ 302.1360; Found 302.1362.

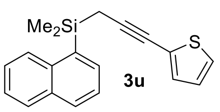

3u

Dimethyl(naphthalen-1-yl)(3-(thiophen-2-yl)prop-2-yn-1-yl)silane (3u)

Prepared according to the general procedure D1 using (8-bromonaphthalen-1-yl)-trimethylsilane (134 mg, 480 µmol, 1.20 equiv) and 3-ethynylthiophene (43.3 mg, 400 µmol, 38.0 µL, 1.00 equiv). The crude material was purified by column chromatography (0 – 15 % (v/v) DCM in pentane) to give **3u** (66 mg, 0.22 mmol, 54 % yield) as a pale-yellow oil.

R_f (10% DCM/Pentane) = 0.42.

^{1}H NMR (400 MHz, $CDCl_3$) δ 8.14 – 8.09 (m, 1H, Ar*H*), 7.93 – 7.86 (m, 2H, Ar*H*), 7.76 (dd, *J* = 6.8, 1.3 Hz, 1H, Ar*H*), 7.57 – 7.45 (m, 3H, Ar*H*), 7.14 (dd, *J* = 5.2, 1.2 Hz, 1H, Ar*H*), 7.04 (dd, *J* = 3.6, 1.1 Hz, 1H, Ar*H*), 6.92 (dd, *J* = 5.2, 3.6 Hz, 1H, Ar*H*), 2.19 (s, 2H, Si-CH_2-C≡C), 0.65 (s, 6H, Si(CH_3)$_2$).
^{13}C{^{1}H} NMR (101 MHz, $CDCl_3$) δ 137.0, 135.4, 134.0, 133.6, 130.6, 130.5, 129.4, 128.0, 126.8, 126.1, 125.64, 125.60, 125.2, 125.1, 92.5, 73.6, 8.4, -1.5.
IR (cm^{-1}) 3071 (s), 2959 (s), 2217 (m), 1505 (m), 1396 (w), 1254 (m), 1147 (m).
HRMS (Sicrit plasma/LTQ-Orbitrap) m/z: $[M + H]^+$ Calcd for $C_{19}H_{19}SSi^+$ 307.0971; Found 307.0968.

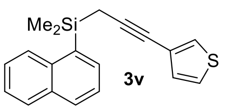

3v

Dimethyl(naphthalen-1-yl)(3-(thiophen-3-yl)prop-2-yn-1-yl)silane (3v)

Prepared according to the general procedure D1 using (8-bromonaphthalen-1-yl)-trimethylsilane (134 mg, 480 µmol, 1.20 equiv) and 3-ethynylthiophene (43.3 mg, 400 µmol, 39.4 µL, 1.00 equiv). The crude material was purified by column chromatography (0 – 15 % (v/v) DCM in pentane) to give **3v** (96 mg, 0.31 mmol, 78 % yield) as a pale-yellow oil.

R_f (10% DCM/Pentane) = 0.42.

^{1}H NMR (400 MHz, $CDCl_3$) δ 8.14 – 8.10 (m, 1H, Ar*H*), 7.92 – 7.85 (m, 2H, Ar*H*), 7.76 (dd, *J* = 6.8, 1.3 Hz, 1H, Ar*H*), 7.55 – 7.45 (m, 3H, Ar*H*), 7.25 (dd, *J* = 3.0, 1.2 Hz, 1H, Ar*H*), 7.21 (dd, *J* = 4.9, 3.0 Hz, 1H, Ar*H*), 7.00 (dd, *J* = 5.0, 1.2 Hz, 1H, Ar*H*), 2.15 (s, 2H, Si-CH_2-C≡C), 0.64 (s, 6H, Si(CH_3)$_2$).
^{13}C{^{1}H} NMR (101 MHz, $CDCl_3$) δ 137.0, 135.6, 134.0, 133.6, 130.5, 130.2, 129.4, 128.1, 127.1, 126.1, 125.6, 125.2, 125.0, 123.7, 87.6, 75.6, 8.0, -1.5.
IR (cm^{-1}) 3108 (w), 3051 (w), 2957 (w), 2219 (w), 1505 (w), 1256 (m), 1148 (w).
HRMS (Sicrit plasma/LTQ-Orbitrap) m/z: $[M + H]^+$ Calcd for $C_{19}H_{19}SSi^+$ 307.0971; Found 307.0969.

D.3. General Procedure for the migratory Sonogashira reaction with aliphatic alkynes.

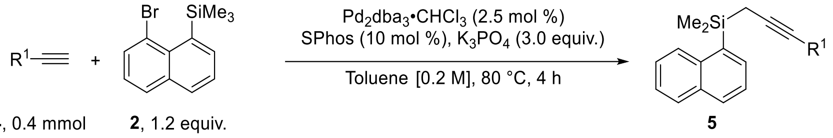

Scheme S6. Migratory Sonogashira reaction for aliphatic substituted alkynes.

An oven-dried 8 mL microwave tube equipped with a Teflon coated stirring bar was charged with SPhos (16.4 mg, 40.0 µmol, 10.0 mol%), Pd_2dba_3•$CHCl_3$ (10.4 mg, 10.0 µmol, 2.5 mol %) and tripotassium phosphate (255 mg, 1.20 mmol, 3.0 equiv) in the glove box. Toluene (1.2 mL) was added and the mixture was stirred at **50 °C for 10 minutes**. Afterwards, a solution of the electrophile (0.480 mmol, 1.2 equiv) in toluene (0.8 mL) and the corresponding alkyne (0.400 mmol) were added. The resulting solution was then stirred at **80 °C for 4 h**. Next, the reaction mixture was allowed to cool down to room temperature and filtered through a plug of silica gel eluting with EtOAc (10 mL) and concentrated in vacuo. The crude material was purified by flash column chromatography on silica gel using a Biotage flash chromatography machine to afford the corresponding product.

D.4. Characterization of the aliphatic propargyl silanes.

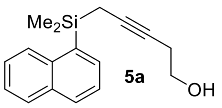

5-(Dimethyl(naphthalen-1-yl)silyl)pent-3-yn-1-ol (5a)

Prepared according to the general procedure D3 using (8-bromonaphthalen-1-yl)-trimethylsilane (134 mg, 480 µmol, 1.20 equiv) and but-3-yn-1-ol (28.0 mg, 30.3 µL, 400 µmol, 1.00 equiv). The crude material was purified by column chromatography (5 – 15 % (v/v) EtOAc in pentane) to give **5a** (58 mg, 0.21 mmol, 54 % yield) as a pale-yellow oil.

R_f (15% EtOAc/Pentane) = 0.65.

^{1}H NMR (400 MHz, $CDCl_3$) δ 8.09 – 8.05 (m, 1H, Ar*H*), 7.91 – 7.86 (m, 2H, Ar*H*), 7.73 (dd, *J* = 6.8, 1.3 Hz, 1H, Ar*H*), 7.55 – 7.44 (m, 3H, Ar*H*), 3.56 (t, *J* = 6.1 Hz, 2H, C≡C-CH_2-CH_2-OH), 2.38 (tt, *J* = 6.1, 2.7 Hz, 2H, C≡C-CH_2-CH_2-OH), 1.93 (t, *J* = 2.7 Hz, 2H, Si-CH_2-C≡C), 0.58 (s, 6H, Si(CH_3)$_2$).
^{13}C{^{1}H} NMR (101 MHz, $CDCl_3$) δ 137.0, 135.6, 133.9, 133.5, 130.5, 129.4, 128.0, 126.0, 125.6, 125.2, 79.9, 76.2, 61.6, 23.5, 7.1, -1.6.
IR (cm^{-1}) 3047 (w), 2956 (m), 1711 (w), 1509 (m), 1254 (m), 1219 (w), 1166 (m), 1146 (m).
HRMS (APPI/LTQ-Orbitrap) m/z: [M]$^+$ Calcd for $C_{17}H_{20}OSi^+$ 268.1278; Found 268.1276.

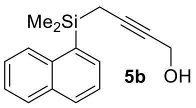

4-(Dimethyl(naphthalen-1-yl)silyl)but-2-yn-1-ol (5b)

Prepared according to the general procedure D3 using (8-bromonaphthalen-1-yl)-trimethylsilane (134 mg, 480 µmol, 1.20 equiv) and prop-2-yn-1-ol (22.4 mg, 23.6 µL, 400 µmol, 1.00 equiv). The crude material was purified by column chromatography (5 – 15 % (v/v) EtOAc in pentane) to give **5b** (32 mg, 0.13 mmol, 31 % yield) as a pale-yellow oil.

R_f (15% EtOAc/Pentane) = 0.71.

^{1}H NMR (400 MHz, $CDCl_3$) δ 8.10 – 8.03 (m, 1H, Ar*H*), 7.92 – 7.86 (m, 2H, Ar*H*), 7.72 (dd, *J* = 6.8, 1.3 Hz, 1H, Ar*H*), 7.56 – 7.44 (m, 3H, Ar*H*), 4.21 (t, *J* = 2.6 Hz, 2H, C≡C-CH_2-OH), 1.98 (t, *J* = 2.6 Hz, 2H, Si-CH_2-C≡C), 0.59 (s, 6H, Si(CH_3)$_2$).
^{13}C{^{1}H} NMR (101 MHz, $CDCl_3$) δ 136.9, 135.4, 133.9, 133.5, 130.5, 129.4, 128.0, 126.0, 125.7, 125.2, 84.3, 78.3, 51.8, 7.2, -1.6.
IR (cm^{-1}) 3055 (w), 2957 (w), 2864 (w), 2218 (w), 1506 (w), 1396 (w), 1256 (m), 1148 (w), 1011 (m).
HRMS (Sicrit plasma/LTQ-Orbitrap) m/z: [M + H]$^+$ Calcd for $C_{16}H_{19}OSi^+$ 255.1200; Found 255.1199.

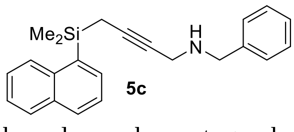

N-Benzyl-4-(dimethyl(naphthalen-1-yl)silyl)but-2-yn-1-amine (5c)

Prepared according to the general procedure D3 using (8-bromonaphthalen-1-yl)-trimethylsilane (134 mg, 480 µmol, 1.20 equiv) and N-benzylprop-2-yn-1-amine (58.1 mg, 400 µmol, 1.00 equiv). The crude material was purified by column chromatography (0 – 40 % (v/v) EtOAc in pentane) to give **5c** (78 mg, 0.23 mmol, 57 % yield) as a pale-yellow oil.

R_f (40% EtOAc/Pentane) = 0.46.

^{1}H NMR (400 MHz, $CDCl_3$) δ 8.12 – 8.07 (m, 1H, Ar*H*), 7.90 – 7.84 (m, 2H, Ar*H*), 7.75 (dd, *J* = 6.8, 1.3 Hz, 1H, Ar*H*), 7.55 – 7.43 (m, 3H, Ar*H*), 7.36 – 7.26 (m, 4H, Ar*H*), 7.25 – 7.22 (m, 1H, Ar*H*), 3.77 (s, 2H Ph-CH_2-NH), 3.38 (t, *J* = 2.5 Hz, 2H, NH-CH_2-C≡C), 1.99 (t, *J* = 2.5 Hz, 2H, Si-CH_2-C≡C), 1.41 (s, 1H, N*H*), 0.61 (s, 6H, Si(CH_3)$_2$).
^{13}C{^{1}H} NMR (101 MHz, $CDCl_3$) δ 139.9, 137.0, 135.6, 133.9, 133.6, 130.4, 129.4, 128.5, 128.5, 128.0, 127.1, 126.0, 125.6, 125.2, 81.1, 77.9, 52.4, 38.2, 7.2, -1.5.
IR (cm^{-1}) 3059 (m), 3032 (m), 2957 (m), 2911 (m), 2842 (m), 1502 (m), 1454 (m), 1324 (w), 1254 (m), 1146 (w).
HRMS (ESI/QTOF) m/z: [M + H]$^+$ Calcd for $C_{23}H_{26}NSi^+$ 344.1829; Found 344.1829.

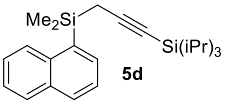

(3-(Dimethyl(naphthalen-1-yl)silyl)prop-1-yn-1-yl)triisopropylsilane (5d)

Prepared according to the general procedure D3 using (8-bromonaphthalen-1-yl)-trimethylsilane (134 mg, 480 µmol, 1.20 equiv) and ethynyl-tri(propan-2-yl)silane (73.0 mg, 89.7 µL, 400 µmol, 1.00 equiv). The crude material was purified by column chromatography (pentane) to give **5d** (74 mg, 0.20 mmol, 49 % yield) as a pale-yellow oil.

R_f (Pentane) = 0.57.

^{1}H NMR (400 MHz, $CDCl_3$) δ 8.09 – 8.02 (m, 1H, Ar*H*), 7.91 – 7.83 (m, 2H, Ar*H*), 7.74 (dd, *J* = 6.8, 1.3 Hz, 1H, Ar*H*), 7.56 – 7.39 (m, 3H, Ar*H*), 2.07 (s, 2H, Si-CH_2-C≡C), 1.09 – 0.97 (m, 21H, Si(C*H*-(CH_3)$_2$)$_3$), 0.61 (s, 6H, Si(CH_3)$_2$).
^{13}C{^{1}H} NMR (101 MHz, $CDCl_3$) δ 136.9, 135.6, 134.0, 133.5, 130.4, 129.3, 128.0, 126.0, 125.6, 125.2, 106.3, 79.9, 18.8, 11.6, 8.9, -1.6.
IR (cm^{-1}) 3061 (w), 2950 (s), 2864 (s), 2157 (m), 1505 (w), 1463 (m), 1386 (w), 1254 (m), 1148 (m).
HRMS (Sicrit plasma/LTQ-Orbitrap) m/z: [M + H]$^+$ Calcd for $C_{24}H_{37}Si_2^+$ 381.2428; Found 381.2428.

D.5. Unsuccessful substrates.

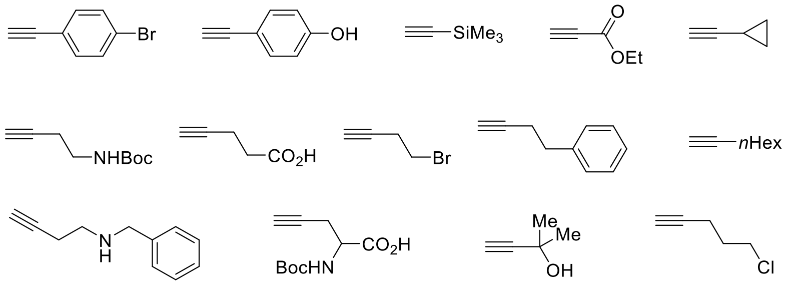

Scheme 7. Unsuccessful substrates in the migratory Sonogashira reaction.

D.6. Addition of propargylic silane to glucal

(3-Acetoxy-6-(1-phenylpropa-1,2-dien-1-yl)-3,6-dihydro-2H-pyran-2-yl)methyl acetate (7)

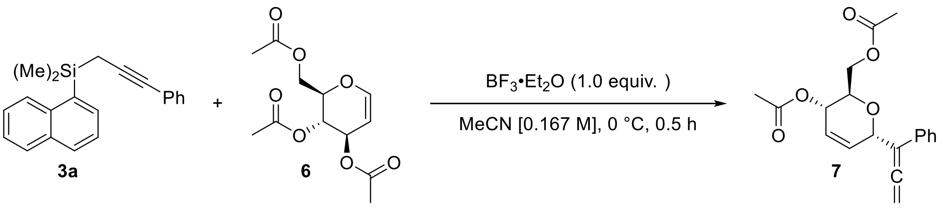

Scheme 8. Addition of propargylic silane to glucal

According to a reported procedure,[9] tri-O-acetyl-D-glucal (172 mg, 632 µmol, 2.0 equiv) was added in an 8 mL oven dried microwave tube. The tube was evacuated and back-filled with N_2 3 times. A solution of the propargyl silane (95 mg, 316 µmol, 1.0 equiv) in MeCN (1.9 mL) was charged and the tube was cooled down to **0 °C**. A solution of boron trifluoride diethyl etherate (45 mg, 39 µL, 316 µmol, 1.0 equiv) in MeCN (0.3 mL) was added and the mixture was stirred at **0 °C for 0.5 h**. The reaction was quenched with sat. aq. $NaHCO_3$ (2 mL), extracted with Et_2O (3x2 mL) dried on $MgSO_4$, filtered and concentrated under vacuo. The crude material was purified by column chromatography (20 – 40 % (v/v) EtOAc in pentane) and then by reverse phase column chromatography (0 – 95 % MeCN in H_2O) to provide product **7** (50 mg, 0.15 mmol, 48 % yield) as a pale-yellow oil.

Rf (30% EtOAc/Pentane) = 0.68.

^{1}H NMR (400 MHz, $CDCl_3$) δ 7.55 – 7.52 (m, 2H, Ar*H*), 7.36 – 7.32 (m, 2H, Ar*H*), 7.25 – 7.22 (m, 1H, Ar*H*), 6.00 (ddd, *J* = 10.2, 3.2, 1.7 Hz, 1H, O-CH-CH=C*H*-), 5.86 (dt, *J* = 10.2, 1.8 Hz, 1H, O-CH-C*H*=CH-), 5.34 – 5.28 (m, 2H, O-C*H*-CH=CH-CH(OAc) and O-CH-CH=CH-C*H*(OAc)), 5.20 (d, *J* = 2.2 Hz, 2H, C=C=CH_2), 4.24 (dd, *J* = 12.0, 6.5 Hz, 1H, AcO-CH_aH_b-), 4.06 (dd, *J* = 12.0, 2.6 Hz, 1H, AcO-CH_aH_b-), 3.95 (ddd, *J* = 8.9, 6.5, 2.6 Hz, 1H, AcOCH_2C*H*-), 2.09 (s, 3H), 1.88 (s, 3H).
^{13}C{^{1}H} NMR (101 MHz, $CDCl_3$) δ 210.0, 170.9, 170.5, 134.4, 130.9, 128.5, 127.1, 126.8, 125.5, 103.8, 79.6, 71.6, 68.6, 65.5, 63.1, 21.1, 20.7.
IR (cm^{-1}) 2924 (w), 1938 (w), 1741 (s), 1494 (w), 1451 (w), 1372 (m), 1235 (s), 1047 (m).
HRMS (ESI/QTOF) m/z: $[M + Na]^+$ Calcd for $C_{19}H_{20}NaO_5^+$ 351.1203; Found 351.1207.

D.7. Hydrogenations of the triple bond
Cinnamyldimethyl(naphthalen-1-yl)silane (8)

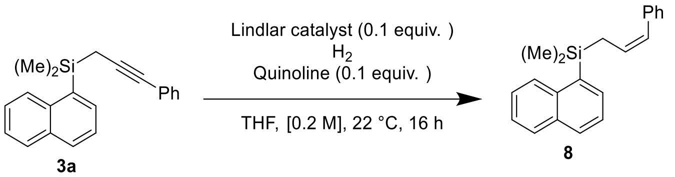

According to a reported procedure,[10] Pd/C poisoned with lead (5% palladium (85.1 mg, 40.0 µmol, 0.100 equiv)) was charged into a 8 mL oven-dried microwave tube. The tube was caped using a rubber septum, put under vacuum and backfilled with nitrogen three times. A solution of alkynyl silane (120 mg, 400 µmol, 1.00 equiv.) in THF (2 mL) and quinoline (5.17 mg, 4.73 µL, 40.0 µmol, 0.100 equiv) were injected into the microwave tube at 22 °C. The tube was filled with H_2 gas (1 atm). The reaction mixture was kept stirring at **22 °C for 16 h**. The solid was filtered off using celite and the filtrate was concentrated under vacuo. The crude material was purified by column chromatography (pentane) to give product **8** (89 mg, 0.29 mmol, 74 % yield) as a colorless oil.

Rf (Pentane) = 0.19.

^{1}H NMR (400 MHz, $CDCl_3$) δ 8.04 – 8.00 (m, 1H, Ar*H*), 7.90 – 7.84 (m, 2H, Ar*H*), 7.67 (dd, *J* = 6.8, 1.3 Hz, 1H, Ar*H*), 7.51 – 7.40 (m, 3H, Ar*H*), 7.26 – 7.20 (m, 4H, Ar*H*), 7.19 – 7.13 (m, 1H, Ar*H*), 6.36 (dt, *J* = 11.6, 1.6 Hz, 1H, CH=C*H*-Ph), 5.74 (dt, *J* = 11.6, 8.9 Hz, 1H, C*H*=CH-Ph), 2.29 (dd, *J* = 9.0, 1.6 Hz, 2H, CH_2-CH=CH-Ph), 0.49 (s, 6H, Si(CH_3)$_2$).
^{13}C{^{1}H} NMR (101 MHz, $CDCl_3$) δ 138.1, 137.0, 136.5, 133.9, 133.6, 130.2, 129.3, 128.7, 128.6, 128.2, 128.1, 127.9, 126.3, 125.9, 125.5, 125.2, 19.2, -1.3.
IR (cm^{-1}) 3053 (m), 3009 (m), 2953 (m), 1633 (w), 1503 (w), 1447 (w), 1390 (w), 1256 (m), 1148 (m).

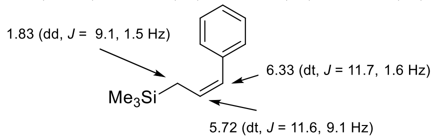

HRMS: not found due to low polarity and poor ionizability of the product. NMR comparison of a related product – cinnamyltrimethysilane - showed consistent chemical shift and coupling constant patterns.[11]

Dimethyl(naphthalen-1-yl)(3-phenylpropyl)silane (9)

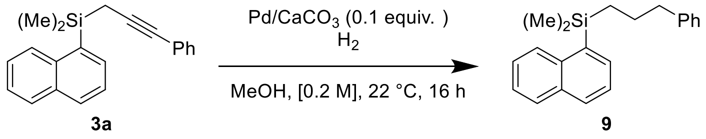

According to a reported procedure,[10] Pd/$CaCO_3$ (5% palladium (85.1 mg, 40.0 µmol, 0.100 equiv)) was charged into an 8 mL oven-dried microwave tube. The tube was caped using a rubber septum, put under vacuum and backfilled with nitrogen three times. A solution of Alkynyl silane (120 mg, 400 µmol, 1.00 equiv) in THF (2 mL) was injected into the microwave tube at 22 °C, and the tube was filled with H_2 gas (1 atm). The reaction mixture was kept stirring at **22 °C for 16 h**. The solid was filtered off using celite and the filtrate was concentrated under vacuo. No purification was needed. The product **9** (120 mg, 0.390 mmol, 99 % yield) was obtained as a pale-yellow oil.

Rf (Pentane) = 0.24.

^{1}H NMR (400 MHz, $CDCl_3$) δ 8.09 – 8.03 (m, 1H, Ar*H*), 7.90 – 7.83 (m, 2H, Ar*H*), 7.66 (dd, *J* = 6.8, 1.3 Hz, 1H, Ar*H*), 7.52 – 7.40 (m, 3H, Ar*H*), 7.26 – 7.22 (m, 2H, Ar*H*), 7.19 – 7.13 (m, 1H, Ar*H*), 7.13 –

7.08 (m, 2H, Ar*H*), 2.61 (t, *J* = 7.6 Hz, 2H, Si-CH_2-CH_2-CH_2-Ph), 1.72 – 1.61 (m, 2H, Si-CH_2-CH_2-CH_2-Ph), 1.07 – 1.00 (m, 2H, Si-CH_2-CH_2-CH_2-Ph), 0.45 (s, 6H, Si(CH_3)$_2$).
<u>^{13}C{^{1}H} NMR</u> (101 MHz, $CDCl_3$) δ 142.6, 137.3, 137.2, 133.7, 133.5, 129.9, 129.3, 128.6, 128.3, 128.2, 125.8, 125.7, 125.4, 125.2, 39.8, 26.3, 16.5, -1.3.
<u>IR</u> (cm^{-1}) 3056 (s), 3027 (s), 2930 (s), 2858 (s), 1501 (m), 1165 (m), 1145 (m), 986 (m).
HRMS (Sicrit plasma/LTQ-Orbitrap) m/z: [M+H-napthalene]$^+$ Calcd for $C_{11}H_{17}Si^+$ 177.1094; Found 177.1067.

D.8. General Procedure for conversion of propargyl silanes to allenes.

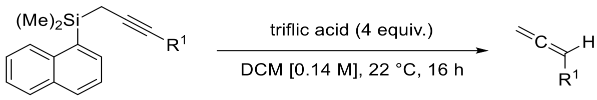

3 → 10

According to a reported procedure,[12] the alkynyl silane (400 µmol, 1.00 equiv) was charged in an oven-dried 8 mL microwave tube as a solution in DCM (2 mL). The tube was cooled down to 0 °C and trifluoromethanesulfonic acid (240 mg, 142 µL, 1.60 mmol, 4.00 equiv) was added. The solution was allowed to warm at **22 °C** and was stirred **16 h**. The reaction mixture was quenched with saturated $NaHCO_3$ (2 mL), the two layers were separated and the aqueous layer was extracted with DCM (3x2mL). The regrouped organic layers were dried on $MgSO_4$ and concentrated under vacuo.

D.9. Characterization data of the allenes

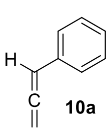

10a

Propa-1,2-dien-1-ylbenzene (10a)

Prepared according to a modified general procedure D8 at -78 °C using dimethyl(naphthalen-1-yl)(3-phenylprop-2-yn-1-yl)silane (120 mg, 400 µmol, 1.00 equiv). The NMR yields were determined using trichloroethylene as an internal standard (13.2 mg, 9.00 µL, 100 µmol, 0.250 equiv). The purification was unsuccessful due to coelution with byproduct.

Selected peaks: <u>^{1}H NMR</u> (400 MHz, $CDCl_3$) δ 6.17 (t, *J* = 6.8 Hz, 1H, CH_2=C=C*H*-Ar), 5.15 (d, *J* = 6.8 Hz, 2H, CH_2=C=CH-Ar).
Allene **10a** is a known compound, the spectral data were consistent with the values reported in literature.
<u>^{1}H NMR yield</u> = 56% by integration of the allene peak at 6.17 ppm.

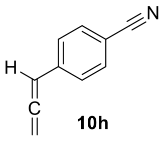

10h

4-(Propa-1,2-dien-1-yl)benzonitrile (10h)

Prepared according to the general procedure D8 using 4-(3-(dimethyl(naphthalen-1-yl)silyl)prop-1-yn-1-yl)benzonitrile (130 mg, 400 µmol, 1.00 equiv).The crude material was purified by column chromatography (10 – 35 % (v/v) DCM in pentane) to give **10h** (50 mg, 0.35 mmol, 89 % yield) as a pale-yellow oil.

<u>^{1}H NMR</u> (400 MHz, $CDCl_3$) δ 7.63 – 7.53 (m, 2H, Ar*H*), 7.42 – 7.32 (m, 2H, Ar*H*), 6.18 (t, *J* = 6.7 Hz, 1H, CH_2=C=C*H*-Ar), 5.24 (d, *J* = 6.7 Hz, 2H, CH_2=C=CH-Ar).
<u>^{13}C{^{1}H} NMR</u> (101 MHz, $CDCl_3$) δ 211.0, 139.4, 132.5, 127.3, 119.2, 110.3, 93.5, 79.9.
Spectral data were consistent with the values reported in literature.[14]

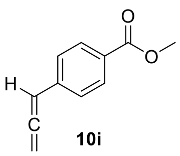

10i

Methyl 4-(propa-1,2-dien-1-yl)benzoate (10i)

Prepared according to the general procedure D8 using methyl 4-(3-(dimethyl(naphthalen-1-yl)silyl)prop-1-yn-1-yl)benzoate (143 mg, 400 µmol, 1.00 equiv).The crude material was purified by column chromatography (10 – 50 % (v/v) DCM in pentane) to give **10i** (54 mg, 0.31 mmol, 77 % yield) as a pale-yellow oil.

<u>^{1}H NMR</u> (400 MHz, $CDCl_3$) δ 8.02 – 7.92 (m, 2H, Ar*H*), 7.39 – 7.31 (m, 2H, Ar*H*), 6.20 (t, *J* = 6.8 Hz, 1H, CH_2=C=C*H*-Ar), 5.21 (d, *J* = 6.8 Hz, 2H, CH_2=C=CH-Ar), 3.91 (s, 3H, COOCH_3).
<u>^{13}C{^{1}H} NMR</u> (101 MHz, $CDCl_3$) δ 210.8, 167.1, 139.2, 130.1, 128.6, 126.7, 93.8, 79.4, 52.2.
Spectral data were consistent with the values reported in literature. [14]

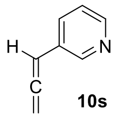

3-(propa-1,2-dien-1-yl)pyridine (10s)

Prepared according to the general procedure D8 using 3-(3-(dimethyl(naphthalen-1-yl)silyl)prop-1-yn-1-yl)pyridine (121 mg, 400 µmol, 1.00 equiv).The crude material was purified by column chromatography (0 – 20 % (v/v) EtOAc in pentane) to give **10s** (41 mg, 0.35 mmol, 87 % yield) as a pale-yellow oil.

R_f (30% EtOAc/Pentane) = 0.49.

^{1}H NMR (400 MHz, $CDCl_3$) δ 8.51 (d, *J* = 2.5 Hz, 1H, Ar*H*), 8.42 (dd, *J* = 4.8, 1.6 Hz, 1H, Ar*H*), 7.61 (dt, *J* = 7.9, 2.0 Hz, 1H, Ar*H*), 7.23 (ddd, *J* = 7.9, 4.8, 0.9 Hz, 1H, Ar*H*), 6.14 (t, *J* = 6.8 Hz, 1H, CH_2=C=C*H*-Ph), 5.20 (d, *J* = 6.8 Hz, 2H, CH_2=C=CH-Ph).
^{13}C{^{1}H} NMR (101 MHz, $CDCl_3$) δ 210.1, 148.2, 148.1, 133.7, 130.1, 123.7, 90.9, 79.6.
IR (cm^{-1}) 2955 (m), 2885 (m), 1748 (w), 1374 (m), 1243 (w), 1046 (w).
HRMS (Sicrit plasma/LTQ-Orbitrap) m/z: [M + H]$^+$ Calcd for $C_8H_8N^+$ 118.0651; Found 118.0651.
Spectral data were consistent with the values reported in literature.[15]

D.10. General Procedure for conversion of propargyl silanes to methyl alkynes.

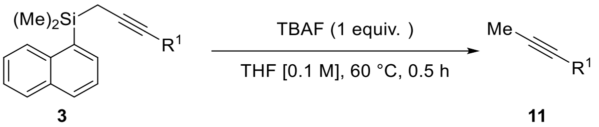

According to a reported procedure,[12] an 8 mL oven dried microwave tube was put under nitrogen atmosphere. A solution of the alkynyl silane (400 µmol, 1.00 equiv) in THF (3.6 mL) was charged into the tube. A solution of tetrabutylammonium fluoride (105 mg, 400 µL, 400 µmol, 1.00 M, 1.00 equiv) in THF was added and the mixture was stirred at **60 °C for 0.5 h**. The reaction was quenched with sat. aq. $NaHCO_3$ (0.5 mL) and extracted with EtOAc (3x2mL). The combined organic layers were dried on $MgSO_4$ and concentrated *in vacuo*.

D.11. Characterization of methyl alkynes

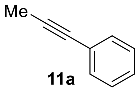

Prop-1-yn-1-ylbenzene (11a)

Prepared according to the general procedure D10 using dimethyl(naphthalen-1-yl)(3-phenylprop-2-yn-1-yl)silane (30.0 mg, 100 µmol, 1.00 equiv). The NMR yields were determined using trichloroethylene as an internal standard (13.2 mg, 9.00 µL, 100 µmol, 1.00 equiv).

The purification was unsuccessful due to coelution with byproduct.

Selected peaks: ^{1}H NMR (400 MHz, $CDCl_3$) δ 6.16 (t, *J* = 6.8 Hz, 1H, PhC*H*=C=CH_2), 5.14 (d, *J* = 6.8 Hz, 2H, PhCH=C=CH_2), 2.04 (s, 3H, PhC≡C-CH_3).
Alkyne **11a** is a known compound, the spectral data were consistent with the values reported in literature.[16]
^{1}H NMR yield = 55% by integration of the methyl peak at 2.04 ppm.
Allene **10a** is a known compound, the spectral data were consistent with the values reported in literature.[14]
^{1}H NMR yield = 37% by integration of the allene peak at 6.16 ppm.

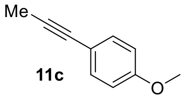

1-Methoxy-4-(prop-1-yn-1-yl)benzene (11c)

Prepared according to the general procedure D10 using (3-(4-methoxyphenyl)prop-2-yn-1-yl)dimethyl(naphthalen-1-yl)silane (33.0 mg, 100 µmol, 1.00 equiv). The NMR yields were determined using trichloroethylene as an internal standard (13.2 mg, 9.00 µL, 100 µmol, 1.00 equiv).

Purification was unsuccessful due to coelution with byproduct.

Selected peaks: ^{1}H NMR (400 MHz, $CDCl_3$) δ 6.12 (t, *J* = 6.8 Hz, 1H, ArC*H*=C=CH_2), 5.12 (d, *J* = 6.8 Hz, 2H, ArCH=C=CH_2), 2.03 (s, 3H, ArC≡C-CH_3).
Alkyne **11c** is a known compound, the spectral data were consistent with the values reported in literature.[17]
^{1}H NMR yield = 49% by integration of the methyl peak at 2.03 ppm.
Allene **10c** is a known compound, the spectral data were consistent with the values reported in literature.[13]
^{1}H NMR yield = 50 % by integration of the allene peak at 6.12 ppm.

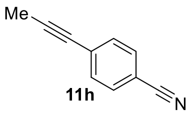

11h

4-(Prop-1-yn-1-yl)benzonitrile (11h)

Prepared according to the general procedure D10 using 4-(3-(dimethyl(naphthalen-1-yl)silyl)prop-1-yn-1-yl)benzonitrile (130 mg, 400 µmol, 1.00 equiv).The crude material was purified by column chromatography (10 – 35 % (v/v) DCM in pentane) to give **11h** (34 mg, 0.24 mmol, 60 % yield) as a white solid.

^{1}H NMR (400 MHz, $CDCl_3$) δ 7.59 – 7.52 (m, 2H, Ar*H*), 7.47 – 7.40 (m, 2H, Ar*H*), 2.07 (s, 3H, C*H*$_3$-C≡C-Ar).
^{13}C{^{1}H} NMR (101 MHz, $CDCl_3$) δ 132.2, 132.1, 129.2, 118.8, 111.0, 91.2, 78.7, 4.7.
Spectral data were consistent with the values reported in literature.[18]

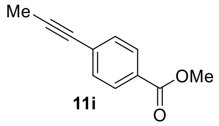

11i

Methyl 4-(prop-1-yn-1-yl)benzoate (11i)

Prepared according to the general procedure D10 using methyl 4-(3-(dimethyl(naphthalen-1-yl)silyl)prop-1-yn-1-yl)benzoate (143 mg, 400 µmol, 1.00 equiv).The crude material was purified by column chromatography (10 – 50 % (v/v) DCM in pentane) to give **11i** (61 mg, 0.35 mmol, 88 % yield) as a white solid.

^{1}H NMR (400 MHz, $CDCl_3$) δ 8.22 – 7.81 (m, 2H, Ar*H*), 7.47 – 7.40 (m, 2H, Ar*H*), 3.91 (s, 3H, COOC*H*$_3$), 2.08 (s, 3H, C*H*$_3$-C≡C-Ar).
^{13}C{^{1}H} NMR (101 MHz, $CDCl_3$) δ 166.8, 131.6, 129.5, 129.0, 129.0, 89.5, 79.4, 52.3, 4.6.
Spectral data were consistent with the values reported in literature.[18]

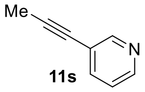

11s

3-(Prop-1-yn-1-yl)pyridine (11s)

Prepared according to the general procedure D10 using 3-(3-(dimethyl(naphthalen-1-yl)silyl)prop-1-yn-1-yl)pyridine (121 mg, 400 µmol, 1.00 equiv). The crude material was purified by column chromatography (20 – 40 % (v/v) EtOAc in pentane) to give **11s** (32 mg, 0.27 mmol, 68 % yield) as a pale-yellow oil.

R_f (30% EtOAc/Pentane) = 0.59.

^{1}H NMR (400 MHz, $CDCl_3$) δ 8.62 (dd, *J* = 2.1, 0.9 Hz, 1H, Ar*H*), 8.48 (dd, *J* = 4.9, 1.7 Hz, 1H, Ar*H*), 7.66 (dt, *J* = 7.9, 2.0 Hz, 1H, Ar*H*), 7.20 (ddd, *J* = 7.9, 4.9, 0.9 Hz, 1H, Ar*H*), 2.07 (s, 3H, C*H*$_3$-C≡C-Ph).
^{13}C{^{1}H} NMR (101 MHz, $CDCl_3$) δ 152.3, 148.0, 138.4, 122.9, 121.2, 89.5, 76.6, 4.4.
IR (cm^{-1}) 3032 (m), 2918 (m), 2258 (m), 2222 (m), 1559 (m), 1478 (s), 1408 (s), 1025 (m).
HRMS (ESI/QTOF) m/z: $[M + H]^+$ Calcd for $C_8H_8N^+$ 118.0651; Found 118.0657.
Spectral data were consistent with the values reported in literature.[18]

D.12. Byproduct of electron rich propargyl silane reaction with electrophile

1,3-Bis((Z)-3-(4-methoxyphenyl)-3-(naphthalen-1-yl)allyl)-1,1,3,3-tetramethyldisiloxane (S6)

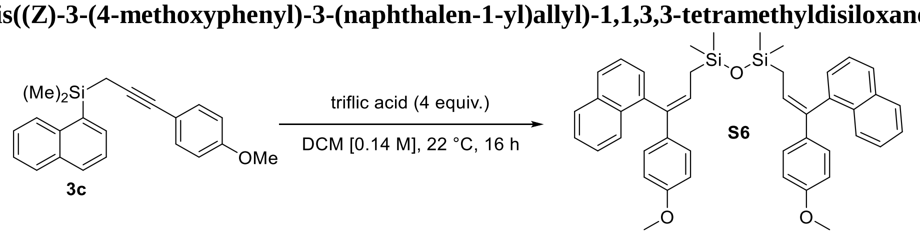

Prepared according to the general procedure D10 using (3-(4-methoxyphenyl)prop-2-yn-1-yl)dimethyl(naphthalen-1-yl)silane (132 mg, 400 µmol, 1.00 equiv).The crude material was purified by column chromatography (20 – 40 % (v/v) DCM in pentane) to give **S6** (121 mg, 0.170 mmol, 89 % yield) as a pale-yellow oil.

R_f (30% DCM/Pentane) = 0.26.

^{1}H NMR (400 MHz, $CDCl_3$) δ 7.86 (d, *J* = 7.6 Hz, 2H, Ar*H*), 7.78 (ddd, *J* = 13.4, 8.5, 2.4 Hz, 4H, Ar*H*), 7.49 – 7.40 (m, 4H, Ar*H*), 7.37 – 7.30 (m, 2H, Ar*H*), 7.29 – 7.26 (m, 2H, Ar*H*), 7.12 – 7.03 (m, 4H, Ar*H*), 6.76 – 6.66 (m, 4H, Ar*H*), 6.34 (dd, *J* = 9.4, 7.6 Hz, 2H, Me_2Si-CH_2-C*H*=C), 3.74 (s, 6H, Ph-O-C*H*$_3$), 1.46 – 1.27 (m, 4H, Me_2Si-C*H*$_2$-CH=C), 0.01 – -0.08 (m, 12H, Si(C*H*$_3$)$_2$).
^{13}C{^{1}H} NMR (101 MHz, $CDCl_3$) δ 158.4, 138.1, 137.4, 135.6, 134.0, 132.2, 128.3, 128.0, 127.4, 127.2, 126.4, 126.0, 125.8, 125.7, 125.2, 113.7, 55.4, 23.2, 0.8.
IR (cm^{-1}) 3061 (w), 2954 (w), 2835 (w), 1774 (w), 1718 (w), 1606 (m), 1510 (s), 1287 (m), 1249 (s).
HRMS (APPI/LTQ-Orbitrap) m/z: $[M + H]^+$ Calcd for $C_{44}H_{47}O_3Si_2^+$ 679.3058; Found 679.3065.

D.13. Degradation study of the electron rich propargyl silane reaction with electrophile product

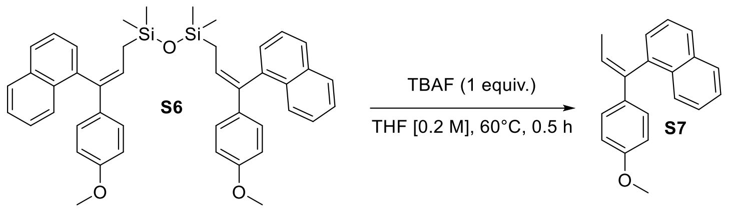

An 8 mL oven dried microwave tube was put under nitrogen atmosphere. A solution of the alkenyl silane dimer **S6** (34.9 mg, 100 µmol, 1.0 equiv) in THF (0.4 mL) was charged into the tube. A solution of tetrabutylammonium fluoride (26.1 mg, 100 µL, 100 µmol, 1.00M, 1.0 equiv) in THF was added and the mixture was stirred at **60 °C for 0.5 h**. The reaction was quenched with 0.5 mL of saturated $NaHCO_3$ and extracted with EtOAc (3x1 mL). The combined organic layers were dried on $MgSO_4$ and concentrated *in vacuo*. The crude material was purified by preparative TLC to give product **S7** (22.0 mg, 80.2 µmol, 80 % yield) as a colorless oil.

R_f (20% DCM/Pentane) = 0.32.

^{1}H NMR (400 MHz, $CDCl_3$) δ 7.90 – 7.86 (m, 1H, Ar*H*), 7.84 (dt, *J* = 8.3, 1.2 Hz, 1H, Ar*H*), 7.77 (dq, *J* = 8.4, 1.0 Hz, 1H, Ar*H*), 7.52 (dd, *J* = 8.3, 7.0 Hz, 1H, Ar*H*), 7.45 (ddd, *J* = 8.2, 6.8, 1.3 Hz, 1H, Ar*H*), 7.37 (ddd, *J* = 8.2, 6.8, 1.4 Hz, 1H, Ar*H*), 7.30 (dd, *J* = 7.0, 1.3 Hz, 1H, Ar*H*), 7.18 – 7.13 (m, 2H, Ar*H*), 6.78 – 6.72 (m, 2H, Ar*H*), 6.43 (q, *J* = 6.9 Hz, 1H, CH_3-C*H*=C), 3.75 (s, 3H, Ar-O-CH_3), 1.53 (d, *J* = 6.9 Hz, 3H, CH_3-CH=C).
^{13}C{^{1}H} NMR (101 MHz, $CDCl_3$) δ 158.7, 139.8, 138.0, 135.0, 134.0, 132.2, 128.4, 127.6, 127.5, 127.4, 126.2, 126.1, 125.8, 125.7, 123.8, 113.7, 55.4, 15.8.
IR (cm^{-1}) 3040 (w), 3003 (w), 2958 (m), 2925 (m), 2852 (w), 1606 (m), 1509 (s), 1290 (m), 1248 (s).
HRMS (Nanochip-based ESI/LTQ-Orbitrap) m/z: $[M + H]^+$ Calcd for $C_{20}H_{19}O^+$ 275.1430; Found 275.1431.

E. X-Ray Crystallographic Data

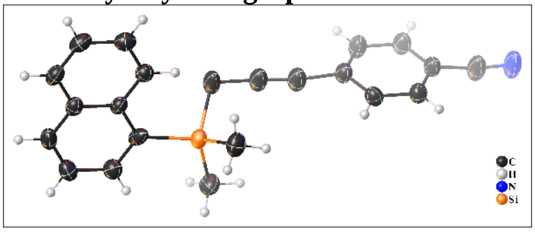

Experimental. Single colourless prism-shaped crystals of **le01-320** were used as supplied. A suitable crystal with dimensions 0.24 × 0.15 × 0.08 mm³ was selected and mounted on a SuperNova, Dual, Cu at home/near, AtlasS2 diffractometer. The crystal was kept at a steady T = 140.00(10) K during data collection. The structure was solved with the **ShelXT** 2018/2 (Sheldrick, 2015) solution program using dual methods and by using **Olex2** 1.5 (Dolomanov et al., 2009) as the graphical interface. The model was refined with **ShelXL** 2018/3 (Sheldrick, 2015) using full-matrix least-squares minimisation on $\boldsymbol{F^2}$.

Crystal Data. $C_{22}H_{19}NSi$, M_r = 325.47, orthorhombic, *Pnma* (No. 62), a = 13.6987(3) Å, b = 7.41005(18) Å, c = 17.6230(4) Å, $\alpha = \beta = \gamma =$ 90°, V = 1788.87(7) Å³, T = 140.00(10) K, Z = 4, Z' = 0.5, μ(Cu K_α) = 1.148, 12136 reflections measured, 1891 unique (R_{int} = 0.0390) which were used in all calculations. The final wR_2 was 0.1731 (all data) and R_1 was 0.0740 (I≥2σ(I)).

**Compound**	**LE01-320**
Formula	$C_{22}H_{19}NSi$
$D_{calc.}$/ g cm⁻³	1.208
μ/mm⁻¹	1.148
Formula Weight	325.47
Colour	colourless
Shape	prism-shaped
Size/mm³	0.24×0.15×0.08
T/K	140.00(10)
Crystal System	orthorhombic
Space Group	*Pnma*
a/Å	13.6987(3)
b/Å	7.41005(18)
c/Å	17.6230(4)
α/°	90
β/°	90
γ/°	90
V/Å³	1788.87(7)
Z	4
Z'	0.5
Wavelength/Å	1.54184
Radiation type	Cu$K\alpha$
Θ_{min}/°	4.087
Θ_{max}/°	72.553
Measured Refl's.	12136
Indep't Refl's	1891
Refl's I≥2σ(I)	1855
R_{int}	0.0390
Parameters	169
Restraints	336
Largest Peak/e Å⁻³	0.441
Deepest Hole/e Å⁻³	-0.351
GooF	1.080
wR_2 (all data)	0.1731
wR_2	0.1727
R_1 (all data)	0.0750
R_1	0.0740
CCDC number	2242318

F. References

(1) Sheldrick, G. M. SHELXT – Integrated Space-Group and Crystal-Structure Determination. *Acta Cryst A* **2015**, *71* (1), 3–8. https://doi.org/10.1107/S2053273314026370.

(2) Sheldrick, G. M. Crystal Structure Refinement with SHELXL. *Acta Cryst C* **2015**, *71* (1), 3–8. https://doi.org/10.1107/S2053229614024218.

(3) Zalesskiy, S. S.; Ananikov, V. P. $Pd_2(Dba)_3$ as a Precursor of Soluble Metal Complexes and Nanoparticles: Determination of Palladium Active Species for Catalysis and Synthesis. *Organometallics* **2012**, *31* (6), 2302–2309. https://doi.org/10.1021/om201217r.

(4) Buzzetti, L.; Puriņš, M.; Greenwood, P. D. G.; Waser, J. Enantioselective Carboetherification/Hydrogenation for the Synthesis of Amino Alcohols via a Catalytically Formed Chiral Auxiliary. *J. Am. Chem. Soc.* **2020**, *142* (41), 17334–17339. https://doi.org/10.1021/jacs.0c09177.

(5) Han, J.-L.; Qin, Y.; Ju, C.-W.; Zhao, D. Divergent Synthesis of Vinyl-, Benzyl-, and Borylsilanes: Aryl to Alkyl 1,5-Palladium Migration/Coupling Sequences. *Angew. Chem., Int. Ed.* **2020**, *59* (16), 6555–6560. https://doi.org/10.1002/anie.201914740.

(6) Hudson, S. A.; McLean, K. J.; Surade, S.; Yang, Y.-Q.; Leys, D.; Ciulli, A.; Munro, A. W.; Abell, C. Application of Fragment Screening and Merging to the Discovery of Inhibitors of the Mycobacterium Tuberculosis Cytochrome P450 CYP121. *Angew. Chem., Int. Ed.* **2012**, *51* (37), 9311–9316. https://doi.org/10.1002/anie.201202544.

(7) Althuon, D.; Rönicke, F.; Fürniss, D.; Quan, J.; Wellhöfer, I.; Jung, N.; Schepers, U.; Bräse, S. Functionalized Triazolopeptoids – a Novel Class for Mitochondrial Targeted Delivery. *Org. Biomol. Chem.* **2015**, *13* (14), 4226–4230. https://doi.org/10.1039/C5OB00250H.

(8) Hess, W.; Burton, J. W. Palladium-Catalysed Cyclisation of N-Alkynyl Aminomalonates. *Chemistry – A European Journal* **2010**, *16* (41), 12303–12306. https://doi.org/10.1002/chem.201001951.

(9) Isobe, M.; Phoosaha, W.; Saeeng, R.; Kira, K.; Yenjai, C. Different C-Glycosidation Products of Glucal with Alkynyl or Propargyl Silanes under Acidic Conditions. *Org. Lett.* **2003**, *5* (25), 4883–4885. https://doi.org/10.1021/ol035957w.

(10) Yang, L.-L.; Ouyang, J.; Zou, H.-N.; Zhu, S.-F.; Zhou, Q.-L. Enantioselective Insertion of Alkynyl Carbenes into Si–H Bonds: An Efficient Access to Chiral Propargylsilanes and Allenylsilanes. *J. Am. Chem. Soc.* **2021**, *143* (17), 6401–6406. https://doi.org/10.1021/jacs.1c03435.

(11) Cai, G.; Zhou, Z.; Wu, W.; Yao, B.; Zhang, S.; Li, X. Pd-Catalyzed C(Sp3)–C(Sp2) Cross-Coupling of Y(CH2SiMe3)3(THF)2 with Vinyl Bromides and Triflates. *Org. Biomol. Chem.* **2016**, *14* (37), 8702–8706. https://doi.org/10.1039/C6OB01765G.

(12) Liu, R.; Hu, R. J.; Zhang, P.; Skolnick, P.; Cook, J. M. Synthesis and Pharmacological Properties of Novel 8-Substituted Imidazobenzodiazepines: High-Affinity, Selective Probes for A5-Containing GABAA Receptors. *J. Med. Chem.* **1996**, *39* (9), 1928–1934. https://doi.org/10.1021/jm950887n.

(13) Woof, C. R.; Durand, D. J.; Webster, R. L. Polymerization of Allenes by Using an Iron(II) β-Diketiminate Pre-Catalyst to Generate High Mn Polymers. *Chem. - Eur. J.* **2021**, *27* (48), 12335–12340. https://doi.org/10.1002/chem.202101078.

(14) Lim, J.; Choi, J.; Kim, H.-S.; Kim, I. S.; Nam, K. C.; Kim, J.; Lee, S. Synthesis of Terminal Allenes via a Copper-Catalyzed Decarboxylative Coupling Reaction of Alkynyl Carboxylic Acids. *J. Org. Chem.* **2016**, *81* (1), 303–308. https://doi.org/10.1021/acs.joc.5b02361.

(15) Nakamura, H.; Onagi, S.; Kamakura, T. Synthesis of Heterocyclic Allenes via Palladium-Catalyzed Hydride-Transfer Reaction of Propargylic Amines. *J. Org. Chem.* **2005**, *70* (6), 2357–2360. https://doi.org/10.1021/jo0479664.

(16) Feofanov, M.; Sharapa, D. I.; Akhmetov, V. Alumina-Mediated Soft Propargylic C–H Activation in Unactivated Alkynes. *Green Chem.* **2022**, *24* (12), 4761–4765. https://doi.org/10.1039/D2GC00555G.

(17) Wigman, B.; Lee, W.; Wei, W.; Houk, K. N.; Nelson, H. M. Electrochemical Fluorination of Vinyl Boronates through Donor-Stabilized Vinyl Carbocation Intermediates**. *Angew. Chem., Int. Ed.* **2022**, *61* (12), e202113972. https://doi.org/10.1002/anie.202113972.

(18) Yoneyama, H.; Numata, M.; Uemura, K.; Usami, Y.; Harusawa, S. Transformation of Carbonyl Compounds into Homologous Alkynes under Neutral Conditions: Fragmentation of Tetrazoles Derived from Cyanophosphates. *J. Org. Chem.* **2017**, *82* (11), 5538–5556. https://doi.org/10.1021/acs.joc.7b00346.

G. NMR Spectra

G.1. Propargyl silanes.

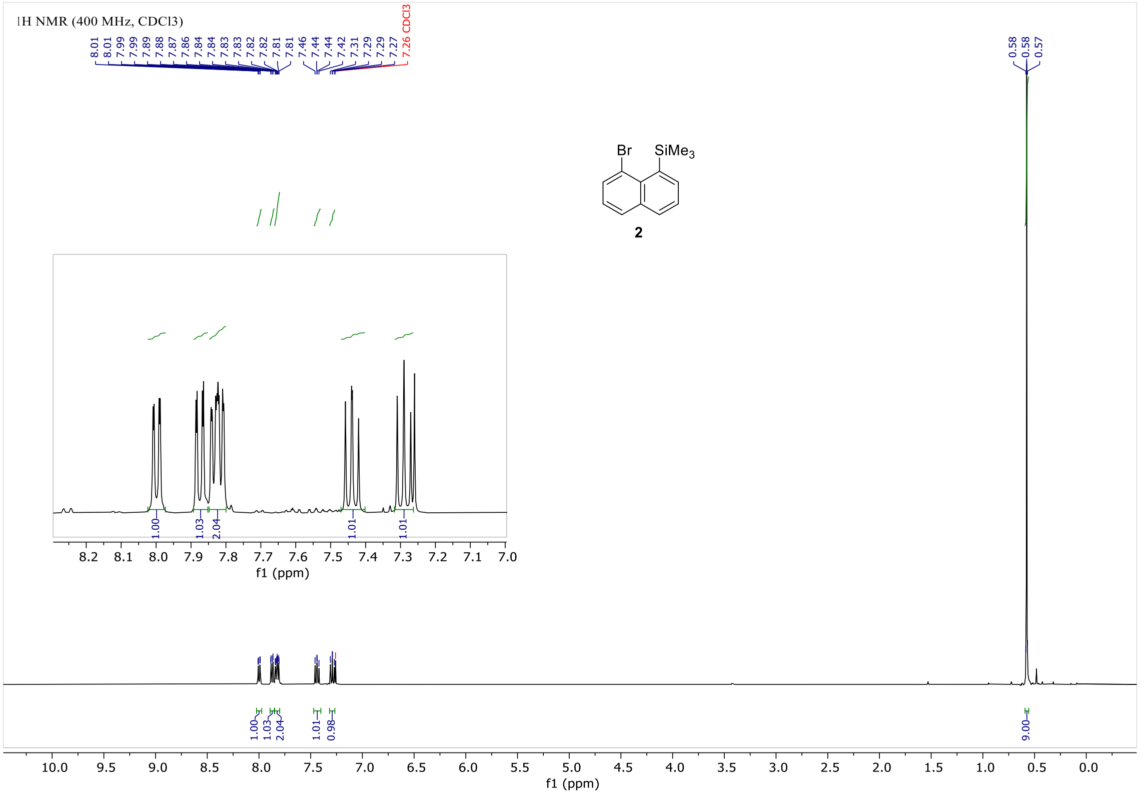

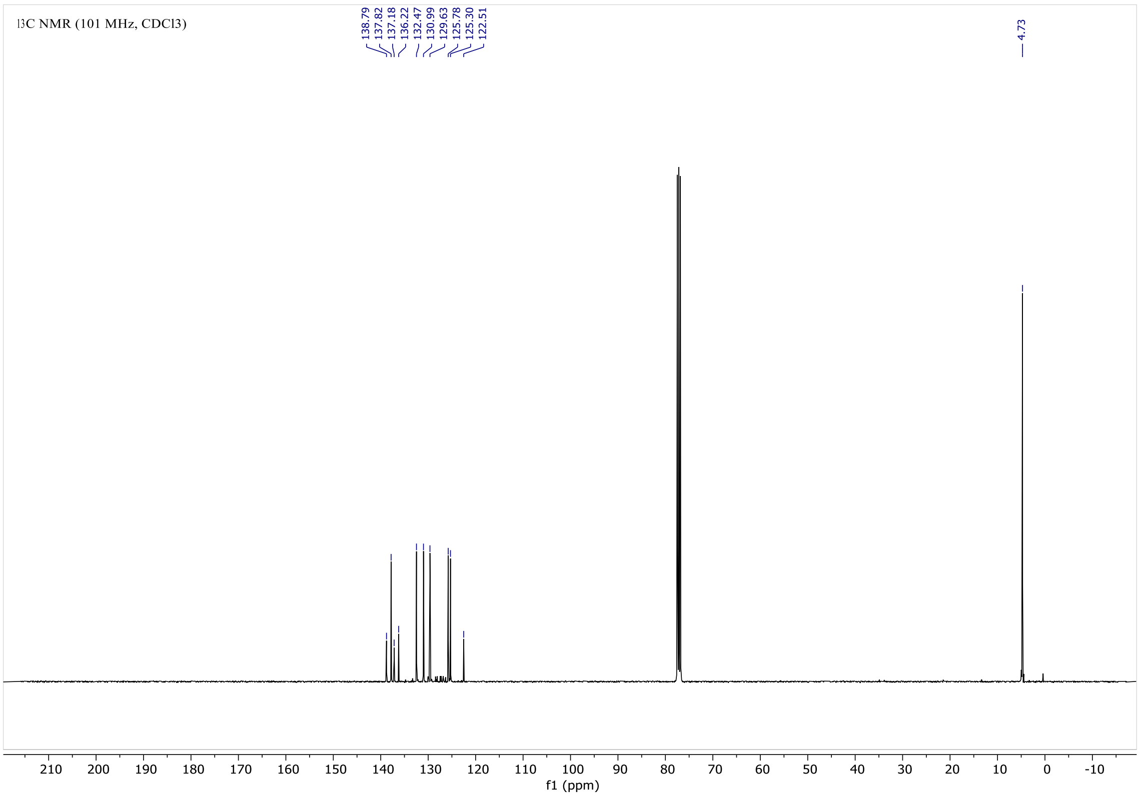

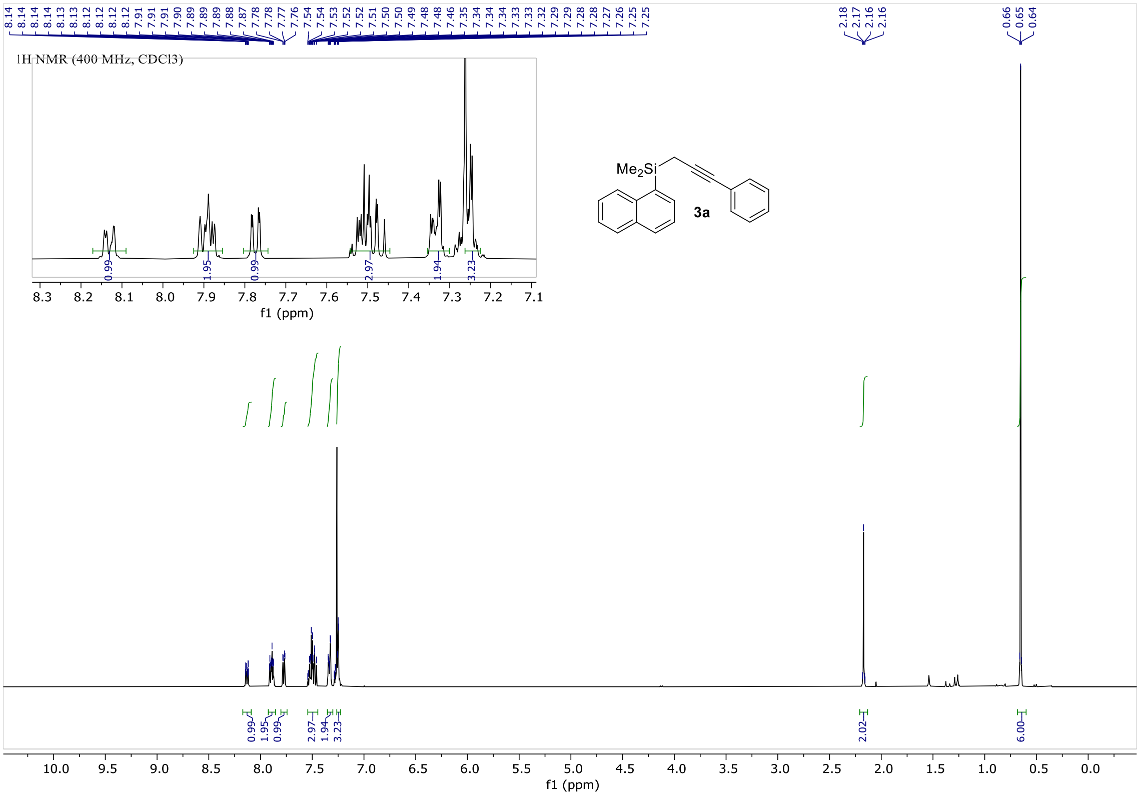
1H NMR (400 MHz, CDCl3)
Me2Si
3a
f1 (ppm)

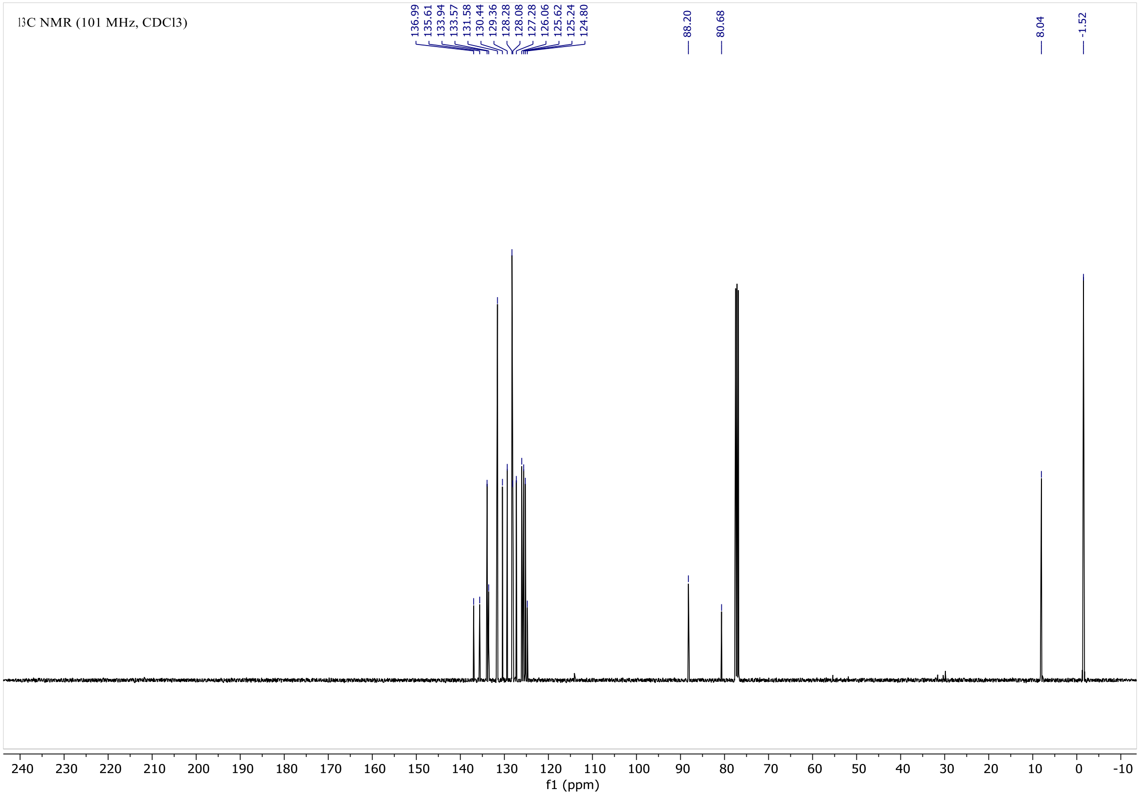
13C NMR (101 MHz, CDCl3)
136.99
135.61
133.94
133.57
131.58
130.44
129.36
128.28
128.08
127.28
126.06
125.62
125.24
124.80
88.20
80.68
8.04
-1.52
f1 (ppm)

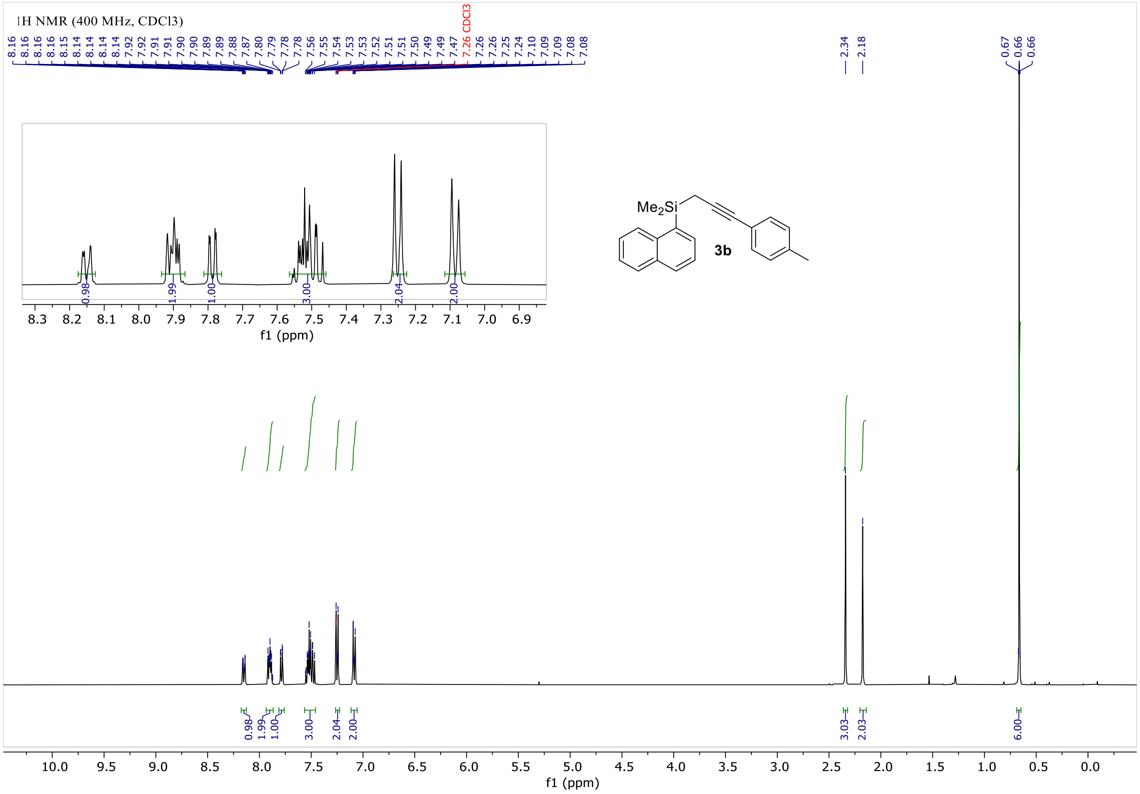
1H NMR (400 MHz, CDCl3)
Me2Si
3b
f1 (ppm)

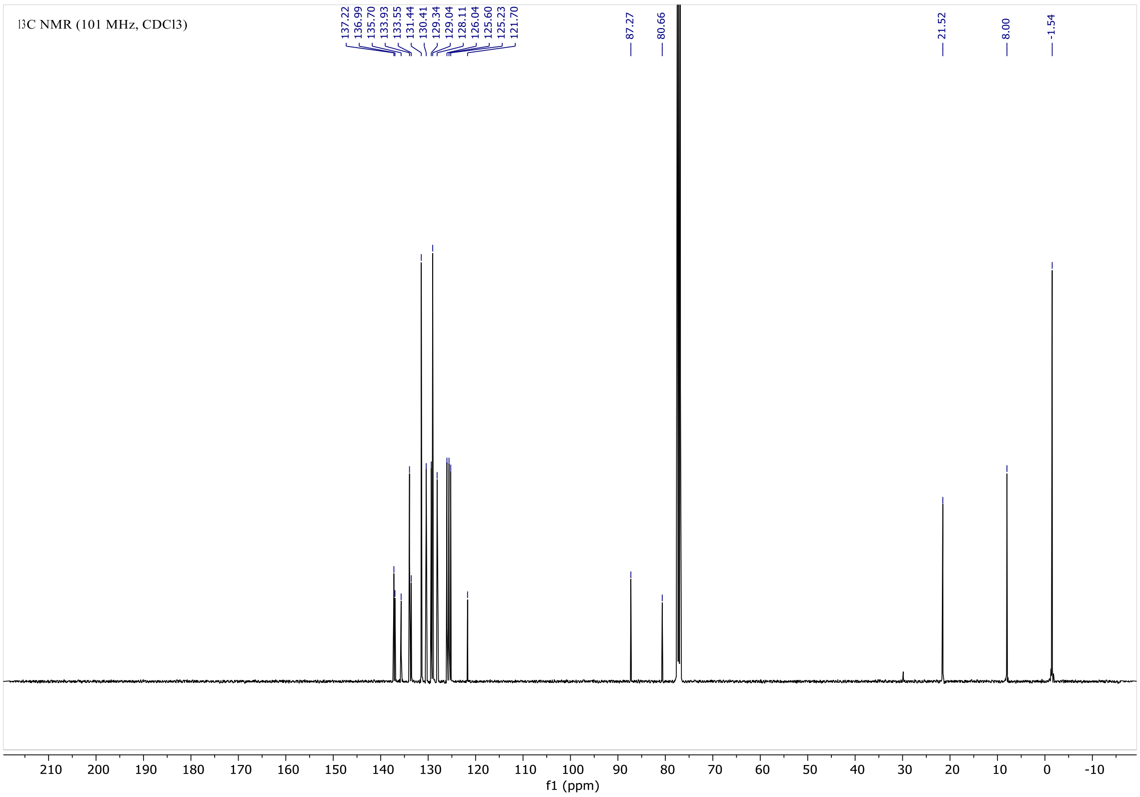
13C NMR (101 MHz, CDCl3)
f1 (ppm)

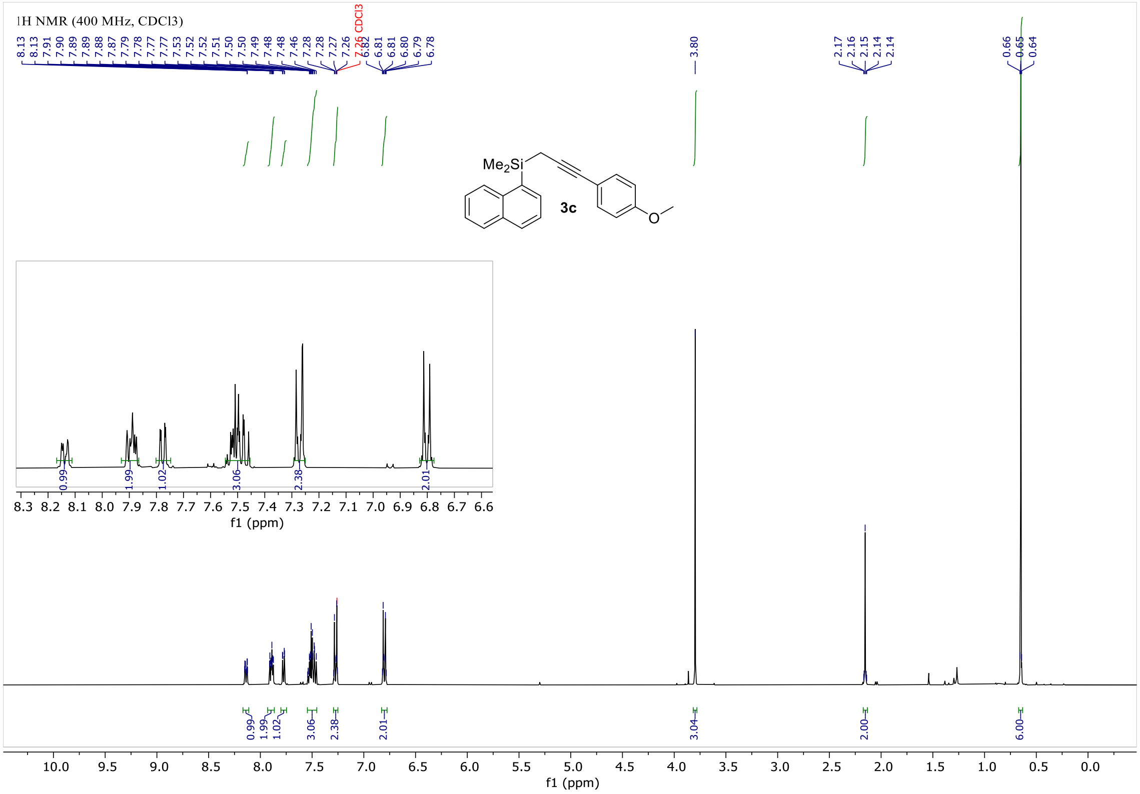
1H NMR (400 MHz, CDCl3)
8.13 8.13 7.91 7.90 7.89 7.89 7.88 7.87 7.79 7.78 7.77 7.77 7.53 7.52 7.52 7.51 7.50 7.50 7.49 7.48 7.48 7.46 7.28 7.28 7.27 7.26
7.26 CDCl3
6.82 6.81 6.81 6.80 6.79 6.78
3.80
2.17 2.16 2.15 2.14 2.14
0.66 0.65 0.64
Me2Si
O
3c
0.99 1.99 1.02 3.06 2.38 2.01 3.04 2.00 6.00
f1 (ppm)

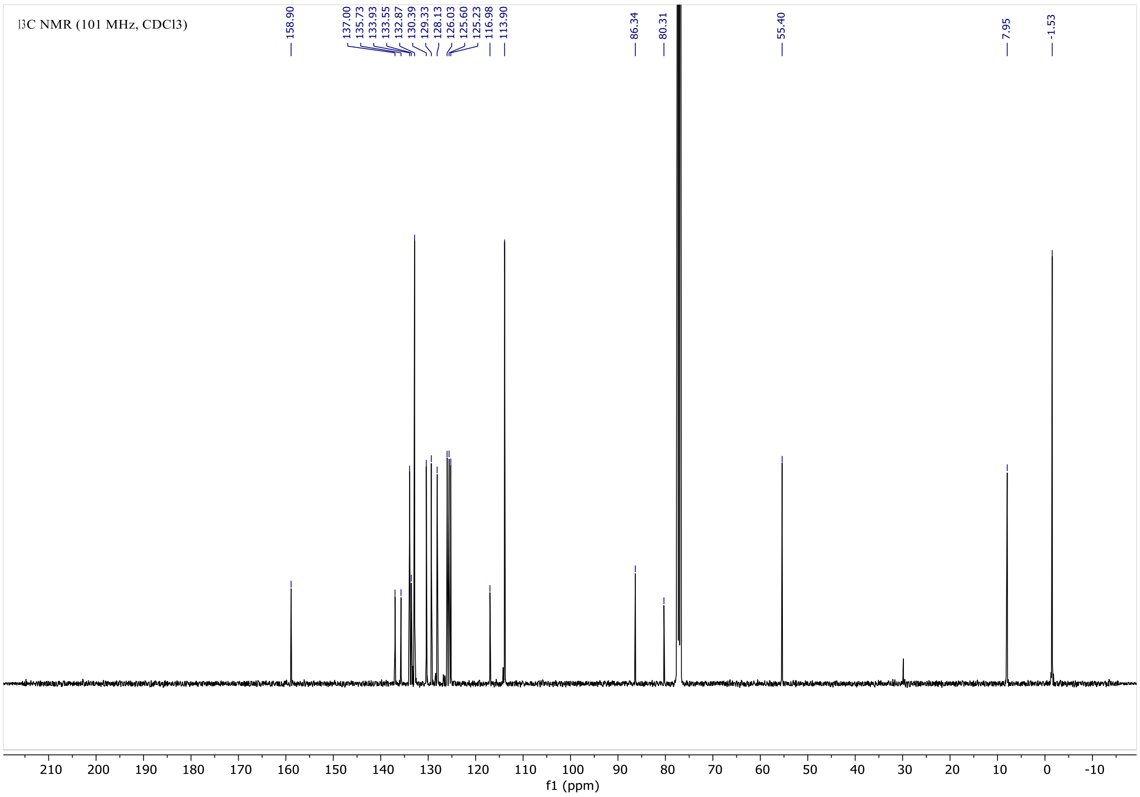
13C NMR (101 MHz, CDCl3)
158.90
137.00 135.73 133.93 133.55 132.87 130.39 129.33 128.13 126.03 125.60 125.23 116.98 113.90
86.34
80.31
55.40
7.95
-1.53
f1 (ppm)

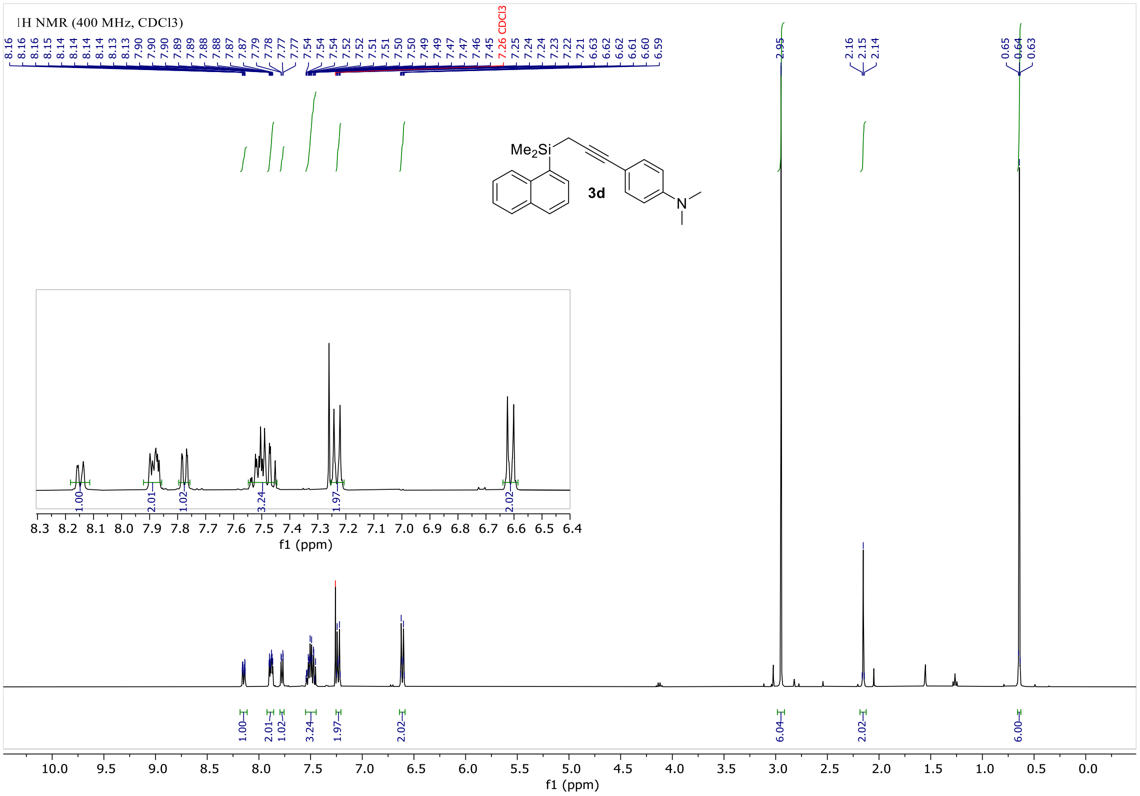
1H NMR (400 MHz, CDCl3)
8.16 8.16 8.15 8.14 8.14 8.14 8.14 8.13 7.90 7.90 7.90 7.89 7.88 7.88 7.87 7.87 7.79 7.78 7.77 7.77 7.54 7.54 7.54 7.52 7.52 7.51 7.51 7.50 7.50 7.49 7.49 7.47 7.46 7.45 7.26 CDCl3 7.25 7.24 7.24 7.23 7.22 7.21 6.63 6.62 6.62 6.61 6.60 6.59
2.95
2.16 2.15 2.14
0.65 0.64 0.63
Me2Si
3d
N
f1 (ppm)

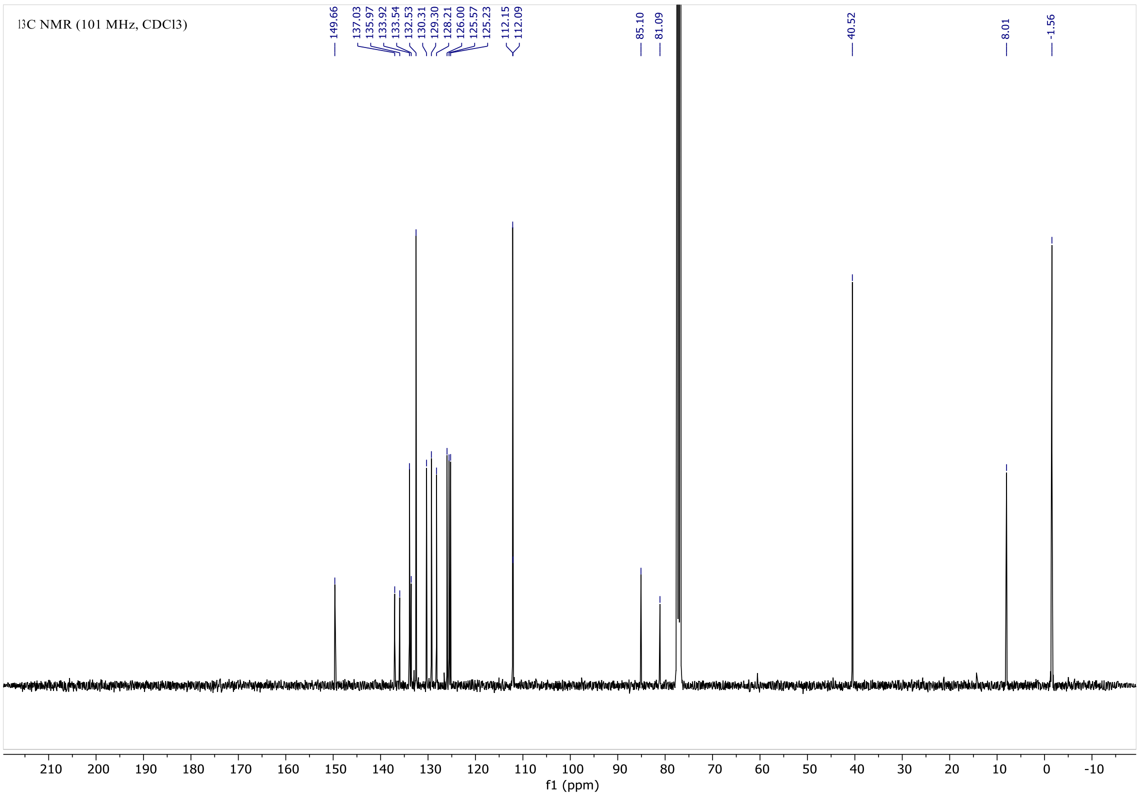
13C NMR (101 MHz, CDCl3)
149.66 137.03 135.97 133.92 133.54 132.53 130.31 129.30 128.21 126.00 125.57 125.23 112.15 112.09
85.10 81.09
40.52
8.01
-1.56
f1 (ppm)

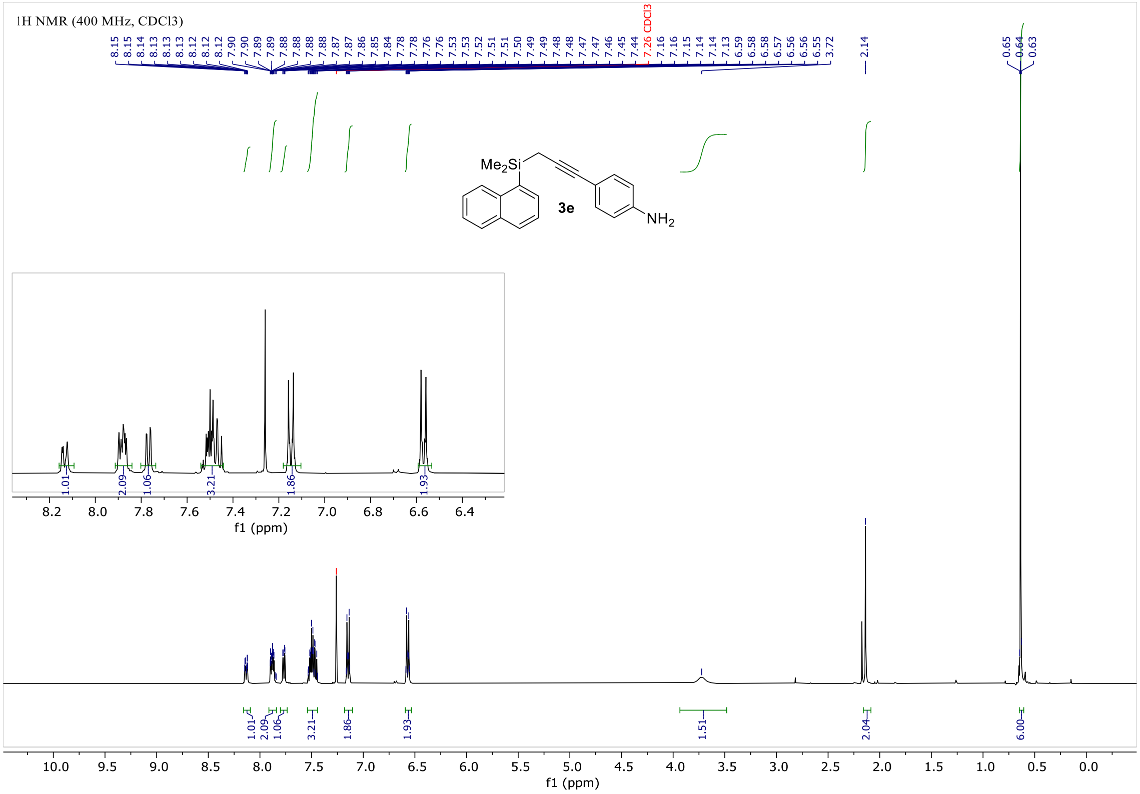
1H NMR (400 MHz, CDCl3)
3e
Me2Si
NH2
f1 (ppm)

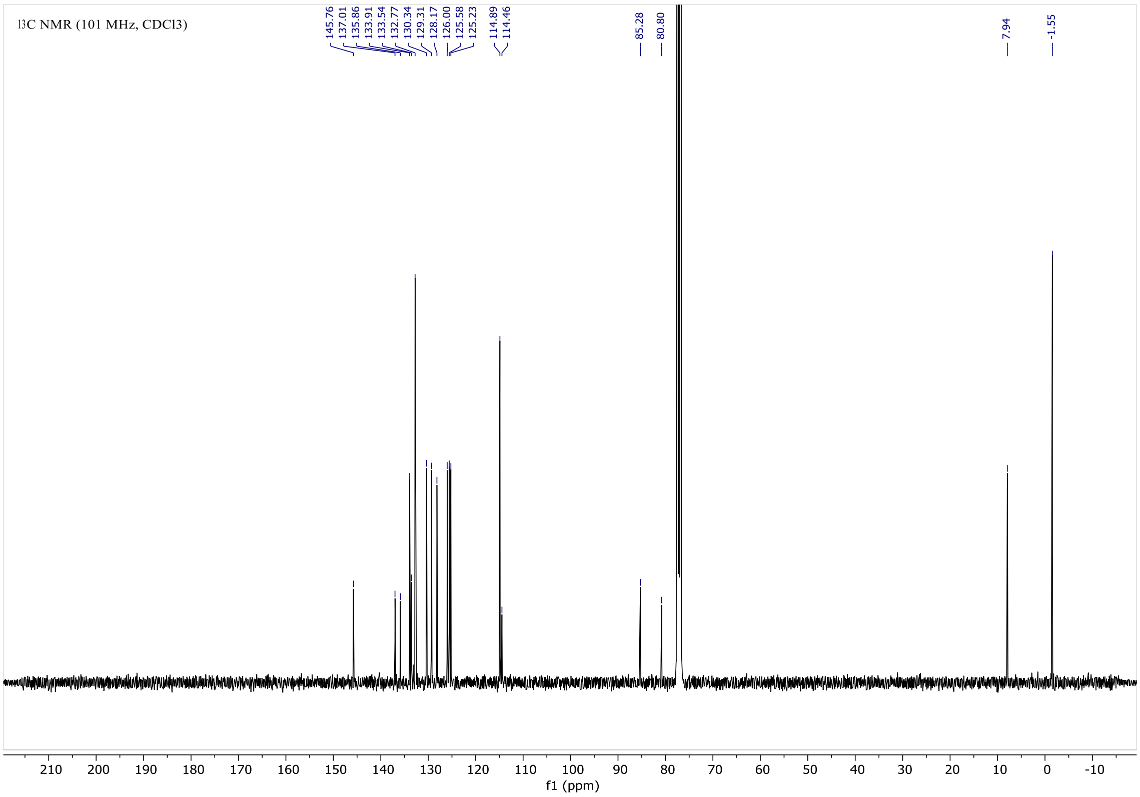
13C NMR (101 MHz, CDCl3)
f1 (ppm)

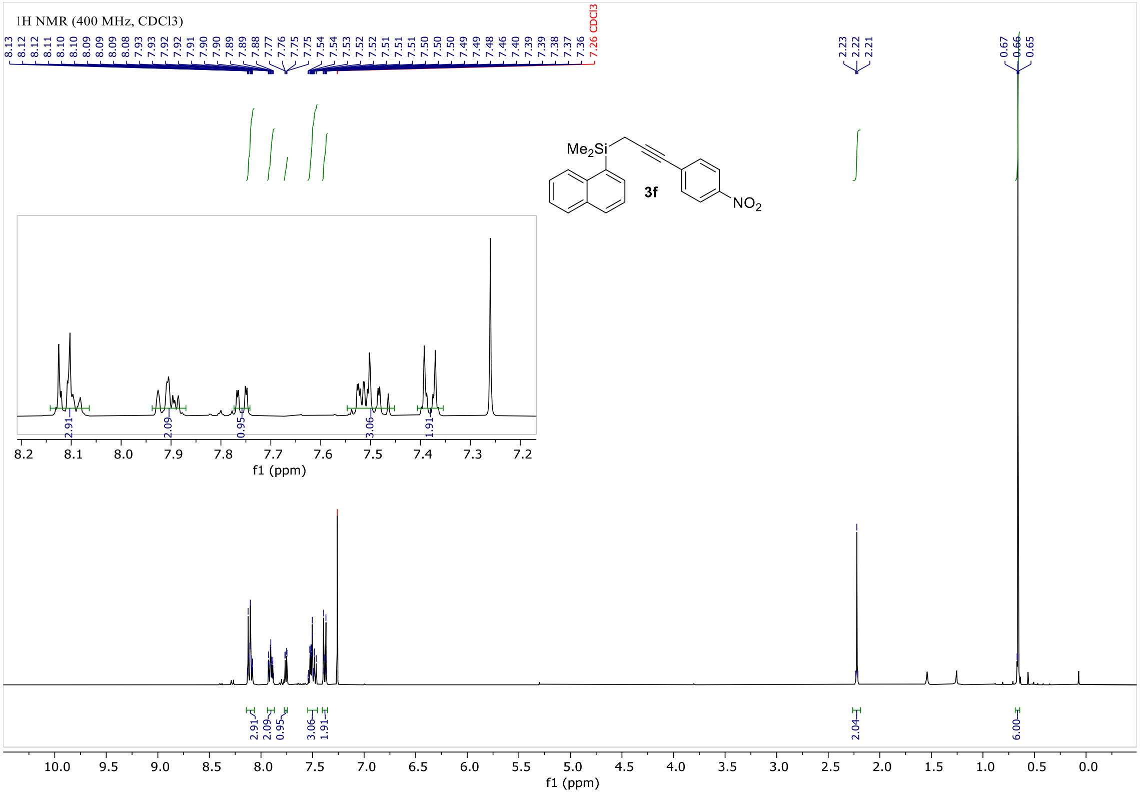
1H NMR (400 MHz, CDCl3)
Me2Si
3f
NO2
f1 (ppm)
f1 (ppm)

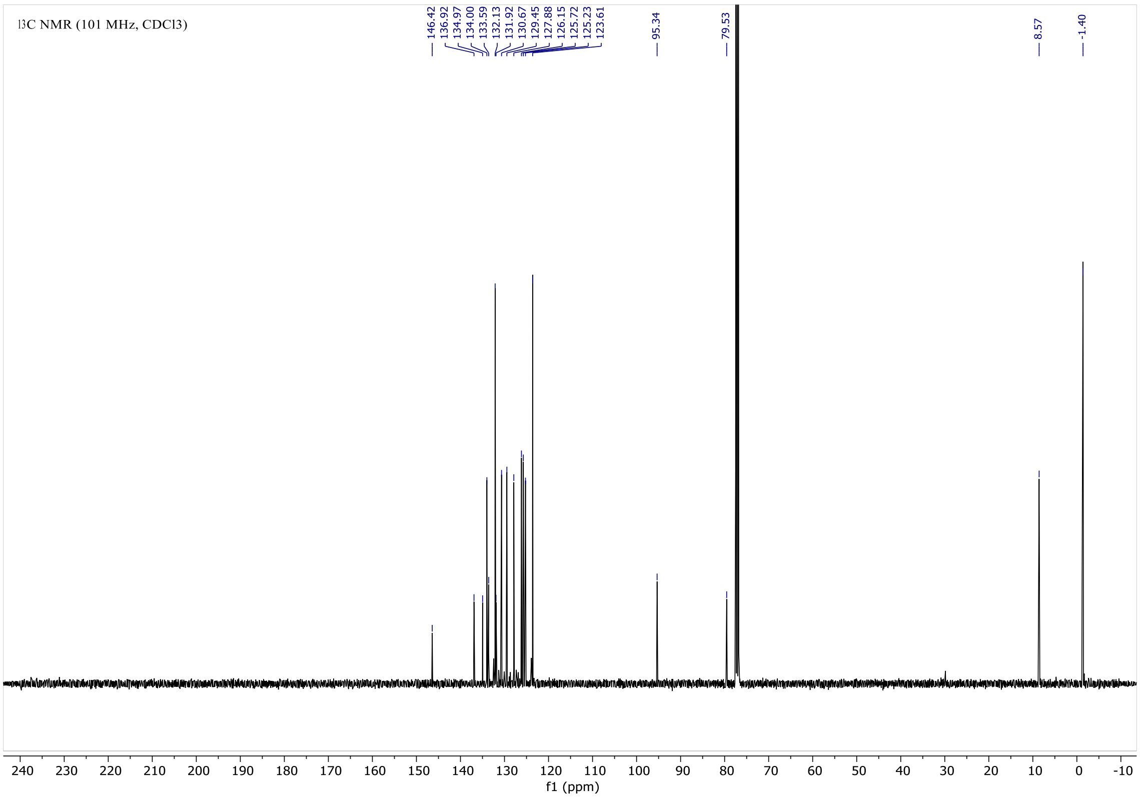
13C NMR (101 MHz, CDCl3)
146.42
136.92
134.97
134.00
133.59
132.13
131.92
130.67
129.45
127.88
126.15
125.72
125.23
123.61
95.34
79.53
8.57
-1.40
f1 (ppm)

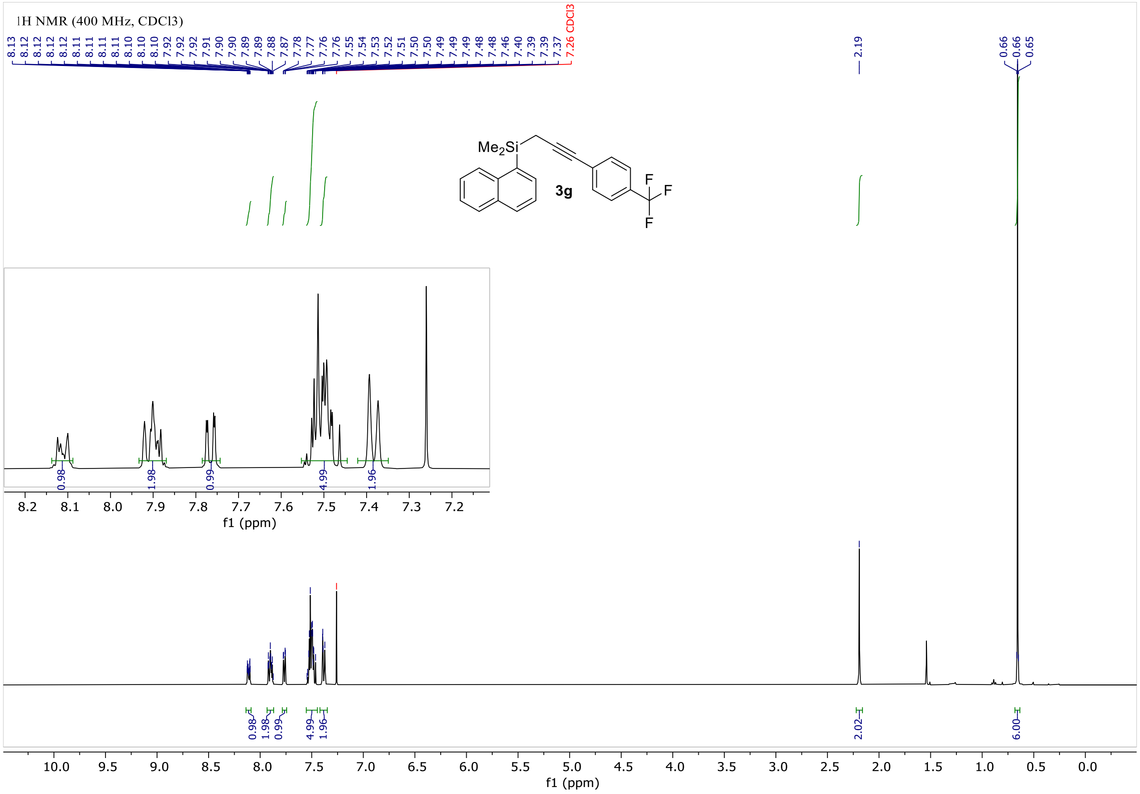
1H NMR (400 MHz, CDCl3)
Me2Si
3g
F
F
F
f1 (ppm)

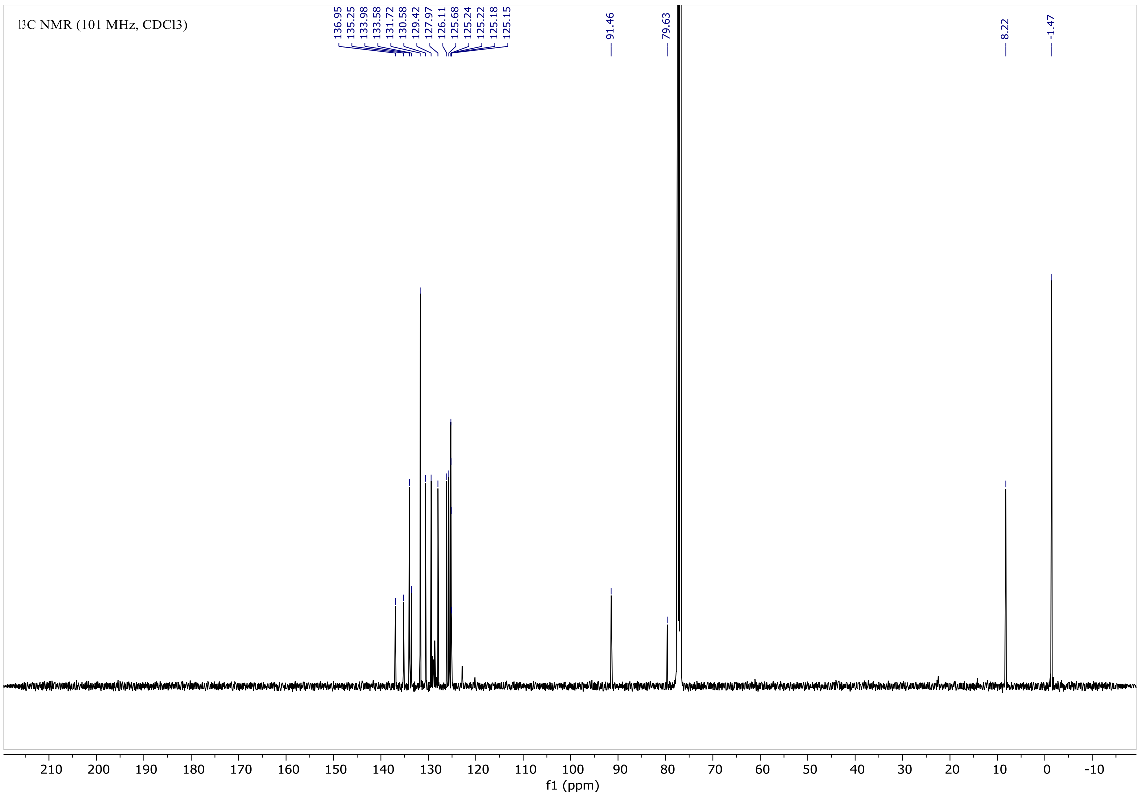
13C NMR (101 MHz, CDCl3)
f1 (ppm)

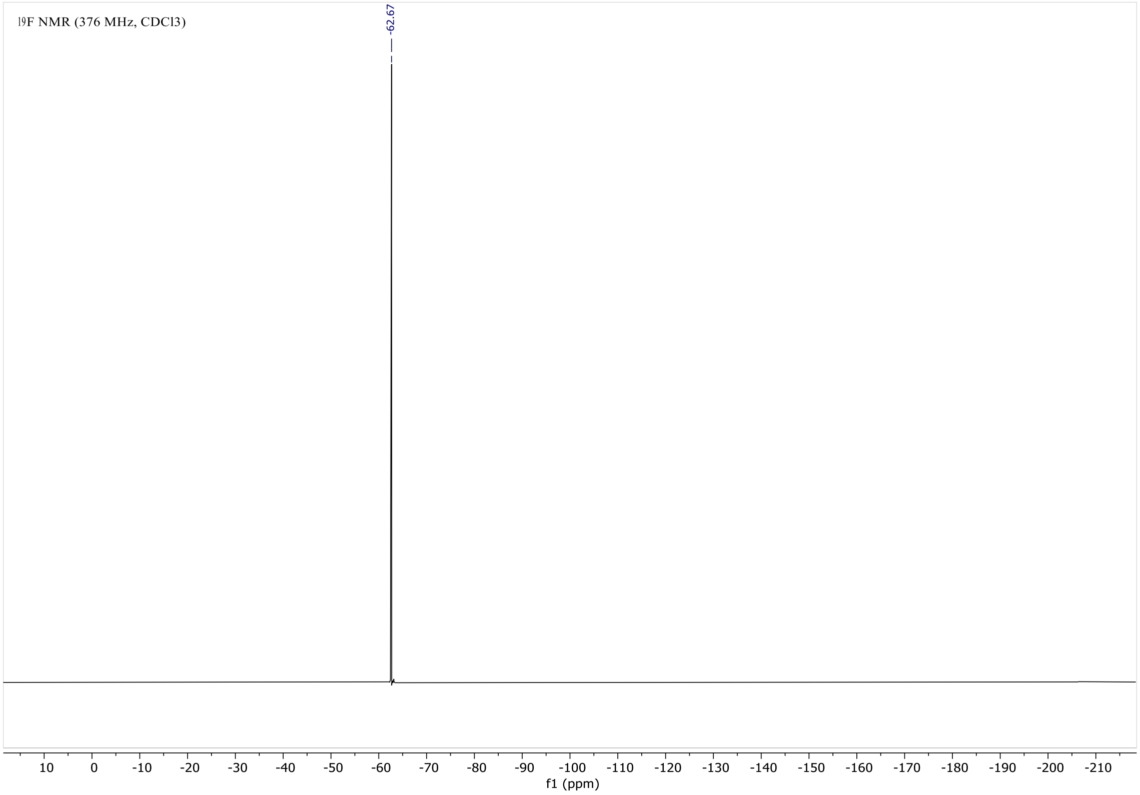

19F NMR (376 MHz, CDCl3)
-62.67
f1 (ppm)

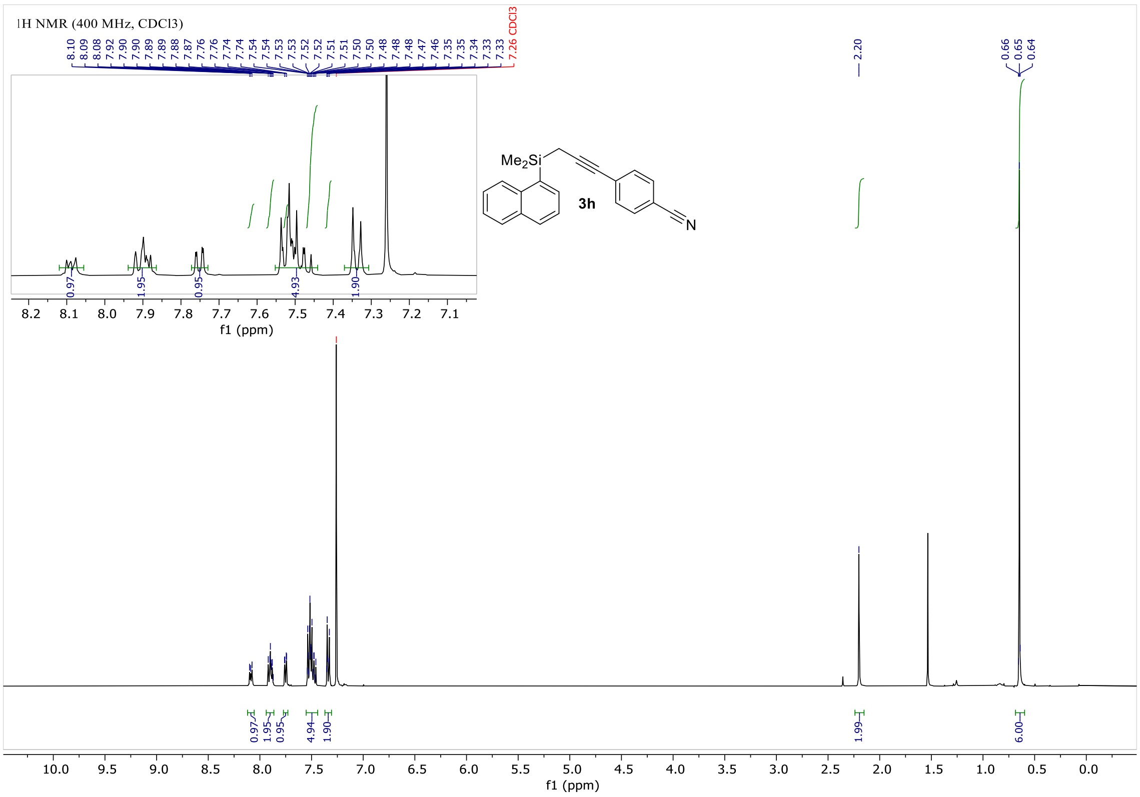

1H NMR (400 MHz, CDCl3)
7.26 CDCl3
2.20
0.66
0.65
0.64
Me2Si
3h
N
f1 (ppm)

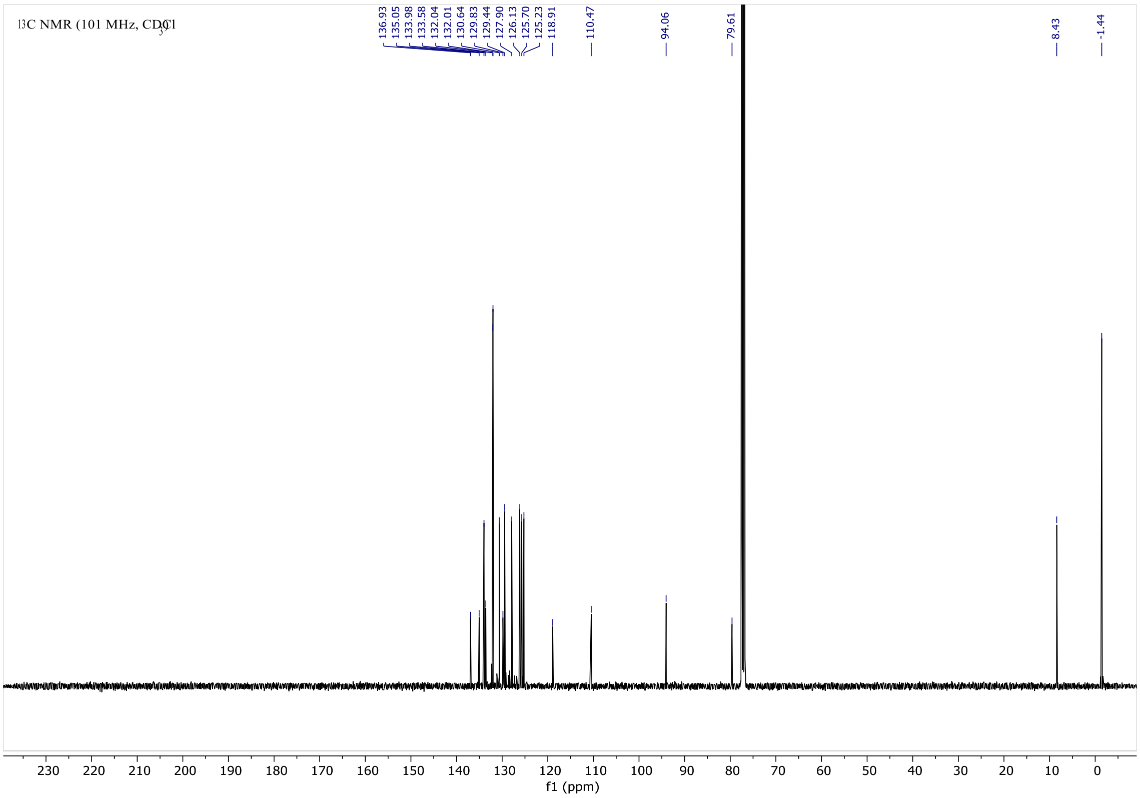
13C NMR (101 MHz, CDCl3)
136.93
135.05
133.98
133.58
132.04
132.01
130.64
129.83
129.44
127.90
126.13
125.70
125.23
118.91
110.47
94.06
79.61
8.43
-1.44
f1 (ppm)

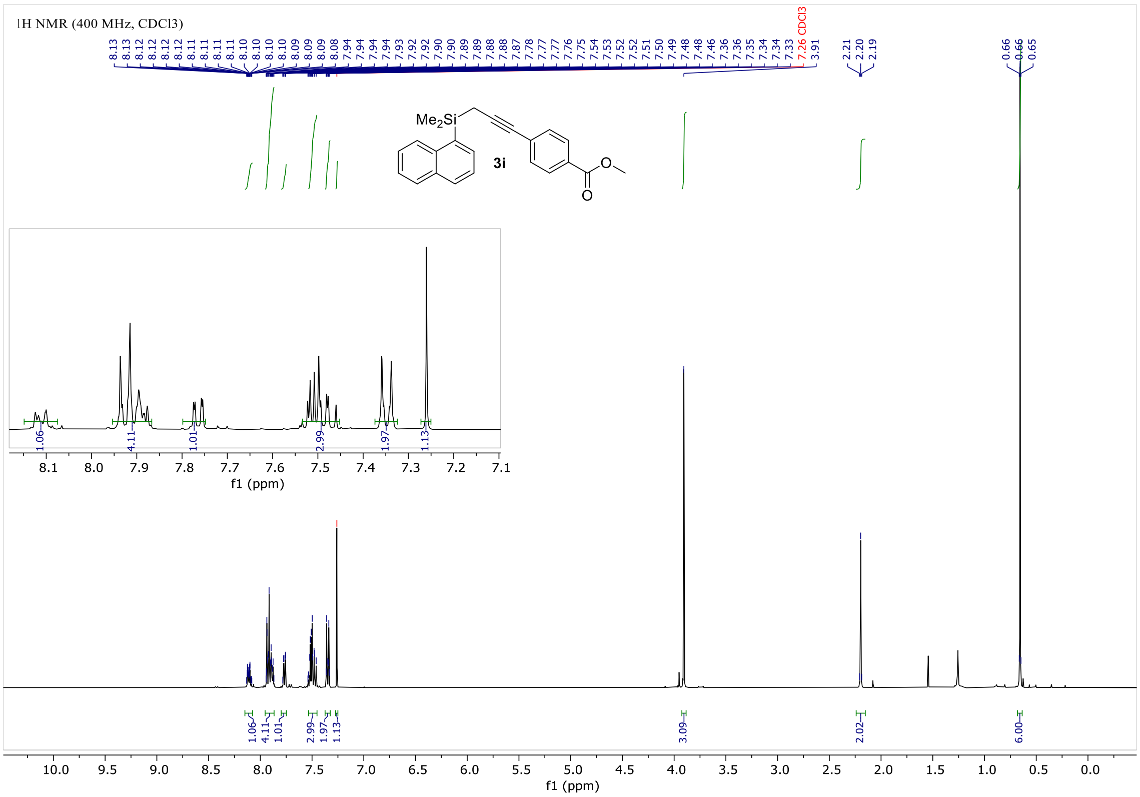
1H NMR (400 MHz, CDCl3)
7.26 CDCl3
3.91
2.21
2.20
2.19
0.66
0.66
0.65
Me2Si
3i
O
1.06
4.11
1.01
2.99
1.97
1.13
3.09
2.02
6.00
f1 (ppm)

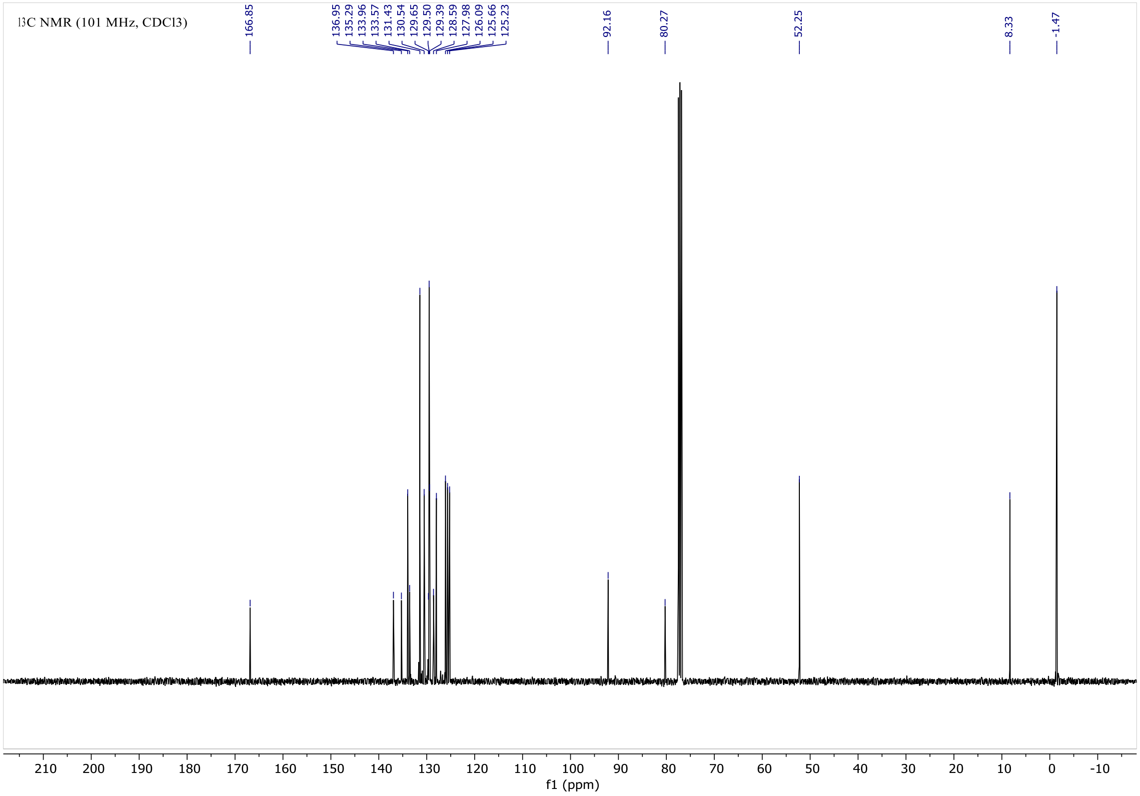
13C NMR (101 MHz, CDCl3)
166.85
136.95
135.29
133.96
133.57
131.43
130.54
129.65
129.50
129.39
128.59
127.98
126.09
125.66
125.23
92.16
80.27
52.25
8.33
-1.47
f1 (ppm)

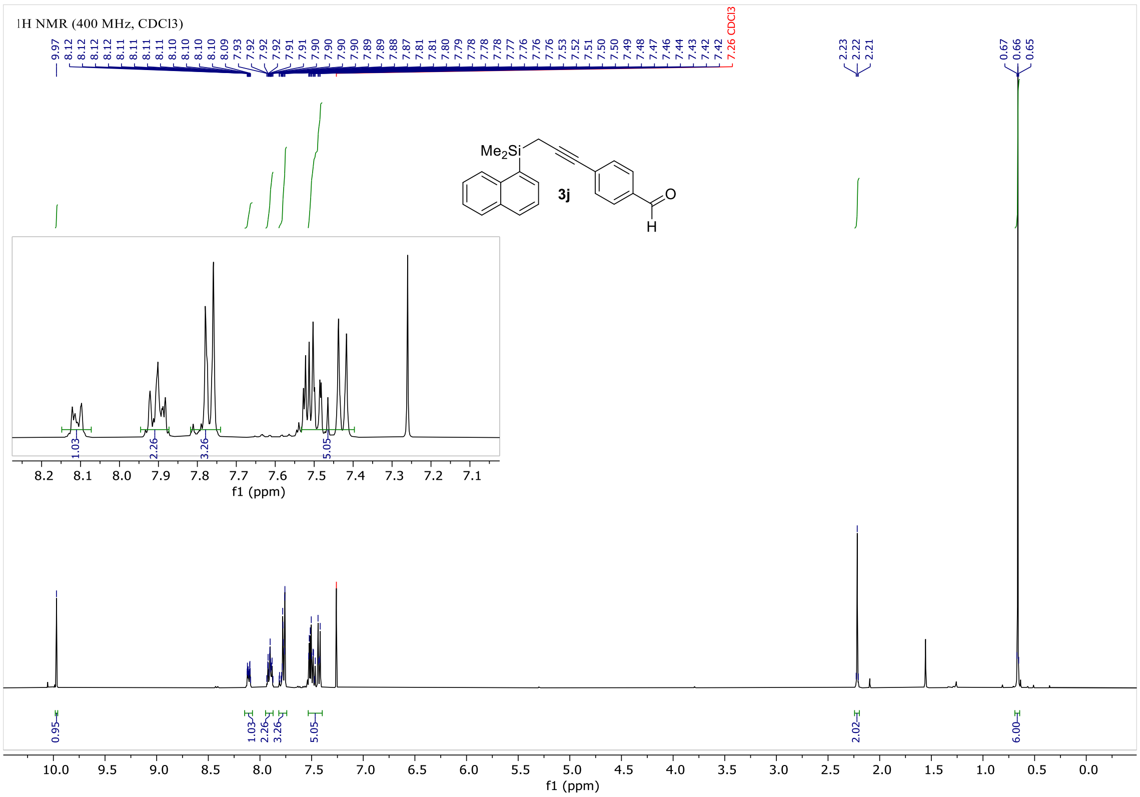
1H NMR (400 MHz, CDCl3)
9.97
8.12
8.12
8.12
8.12
8.11
8.11
8.11
8.10
8.10
8.10
8.09
7.93
7.92
7.92
7.92
7.91
7.91
7.90
7.90
7.90
7.90
7.89
7.89
7.88
7.87
7.81
7.81
7.80
7.79
7.78
7.78
7.78
7.77
7.76
7.76
7.53
7.52
7.51
7.50
7.50
7.49
7.48
7.47
7.46
7.44
7.43
7.43
7.42
7.42
7.26 CDCl3
2.23
2.22
2.21
0.67
0.66
0.65
Me2Si
3j
O
H
1.03
2.26
3.26
5.05
0.95
2.02
6.00
f1 (ppm)

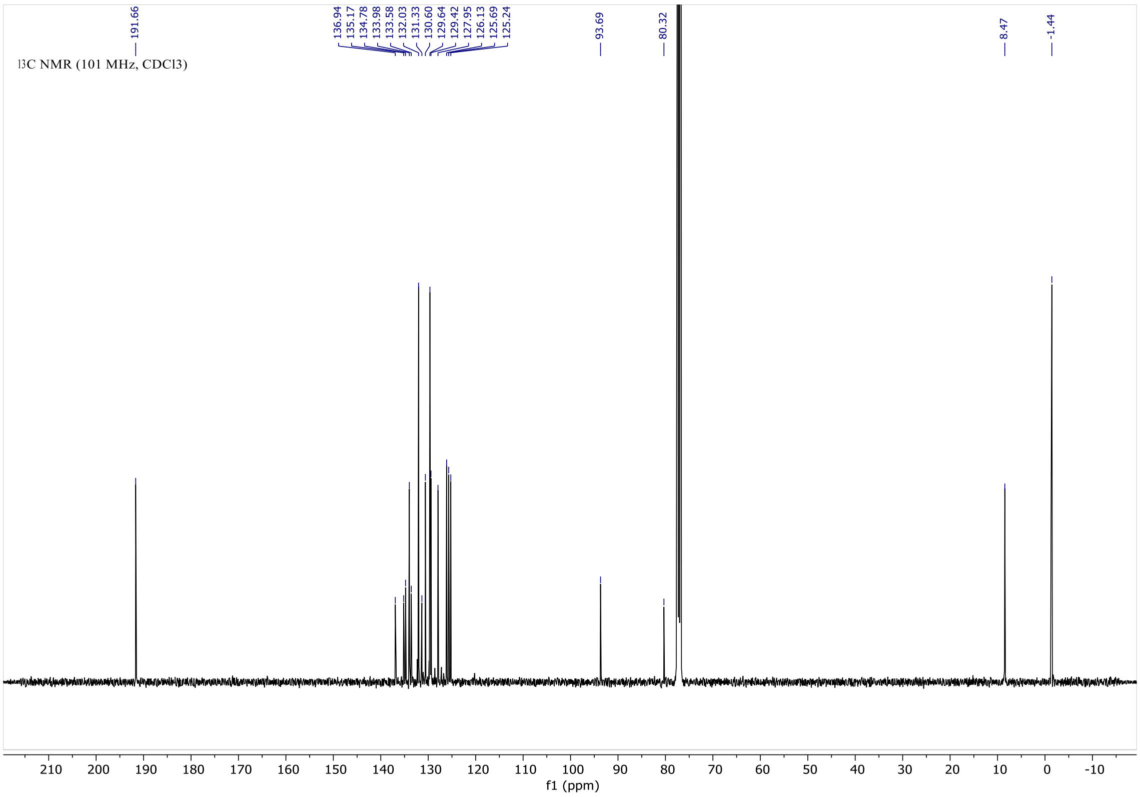
13C NMR (101 MHz, CDCl3)
191.66
136.94 135.17 134.78 133.98 133.58 132.03 131.33 130.60 129.64 129.42 127.95 126.13 125.69 125.24
93.69
80.32
8.47
-1.44
f1 (ppm)

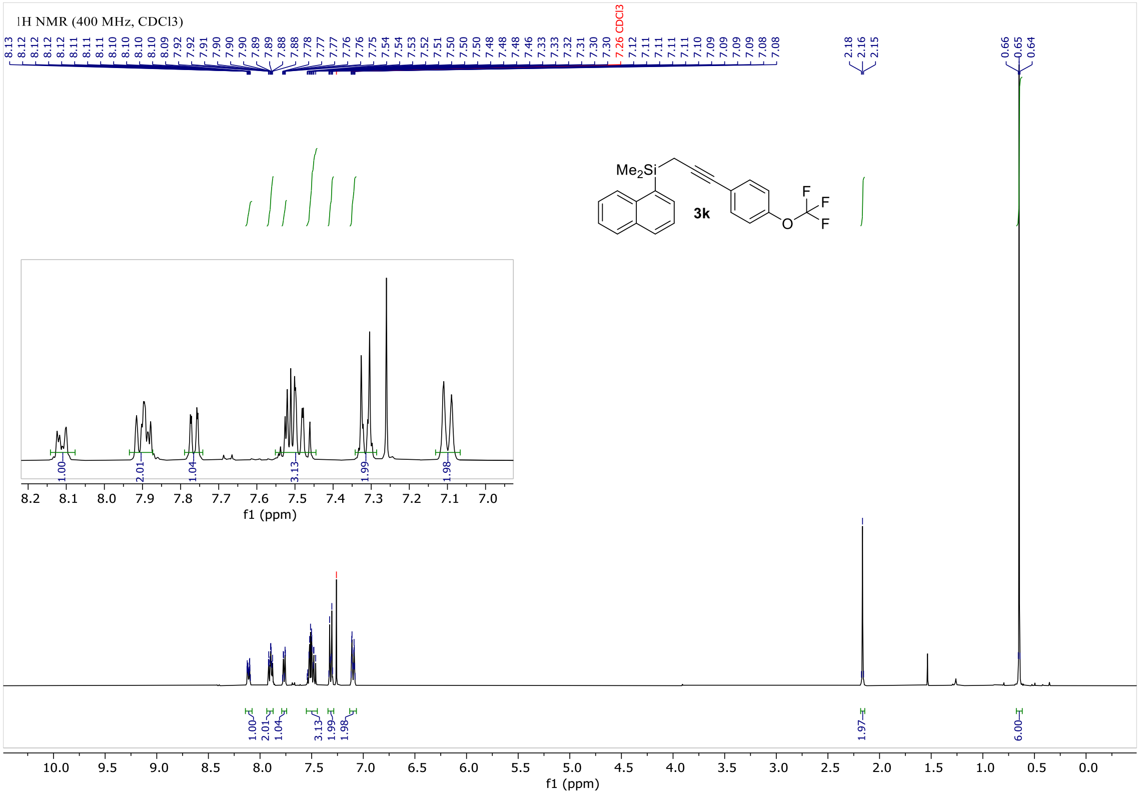
1H NMR (400 MHz, CDCl3)
Me2Si
3k
F
F
F
O
2.18 2.16 2.15
0.66 0.65 0.64
7.26 CDCl3
f1 (ppm)

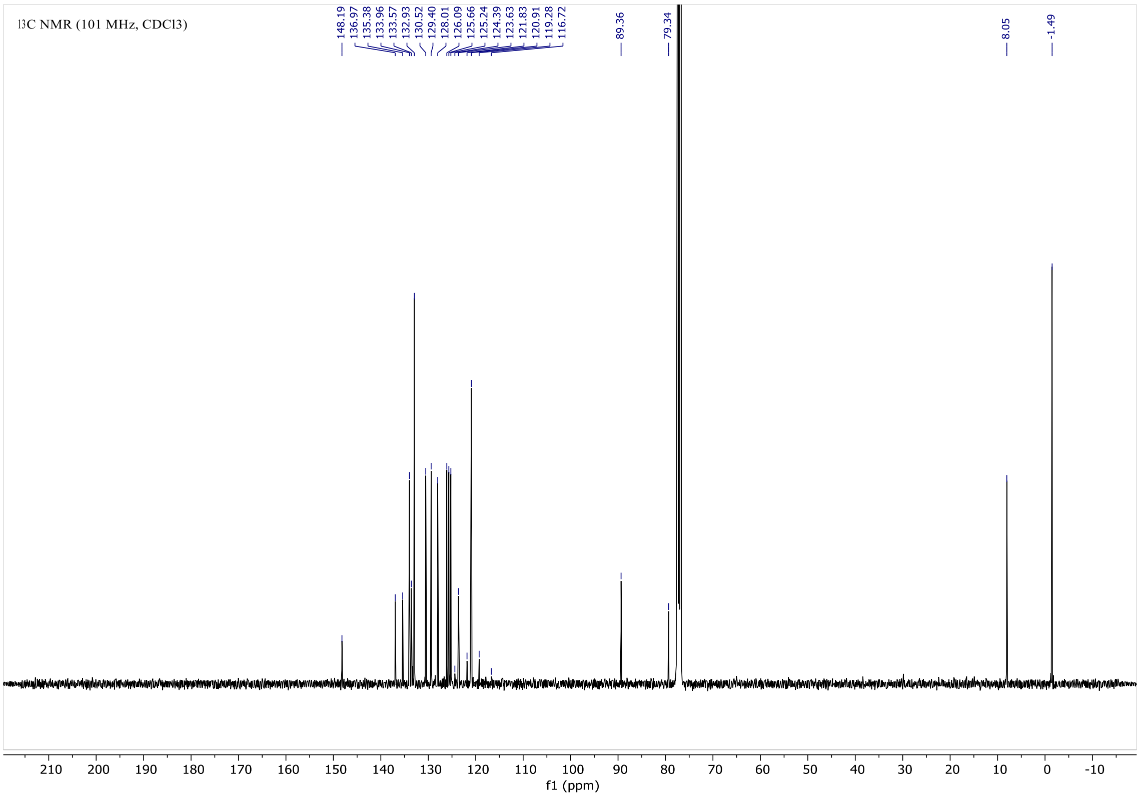

13C NMR (101 MHz, CDCl3)
148.19
136.97
135.38
133.96
133.57
132.93
130.52
129.40
128.01
126.09
125.66
125.24
124.39
123.63
121.83
120.91
119.28
116.72
89.36
79.34
8.05
-1.49
f1 (ppm)

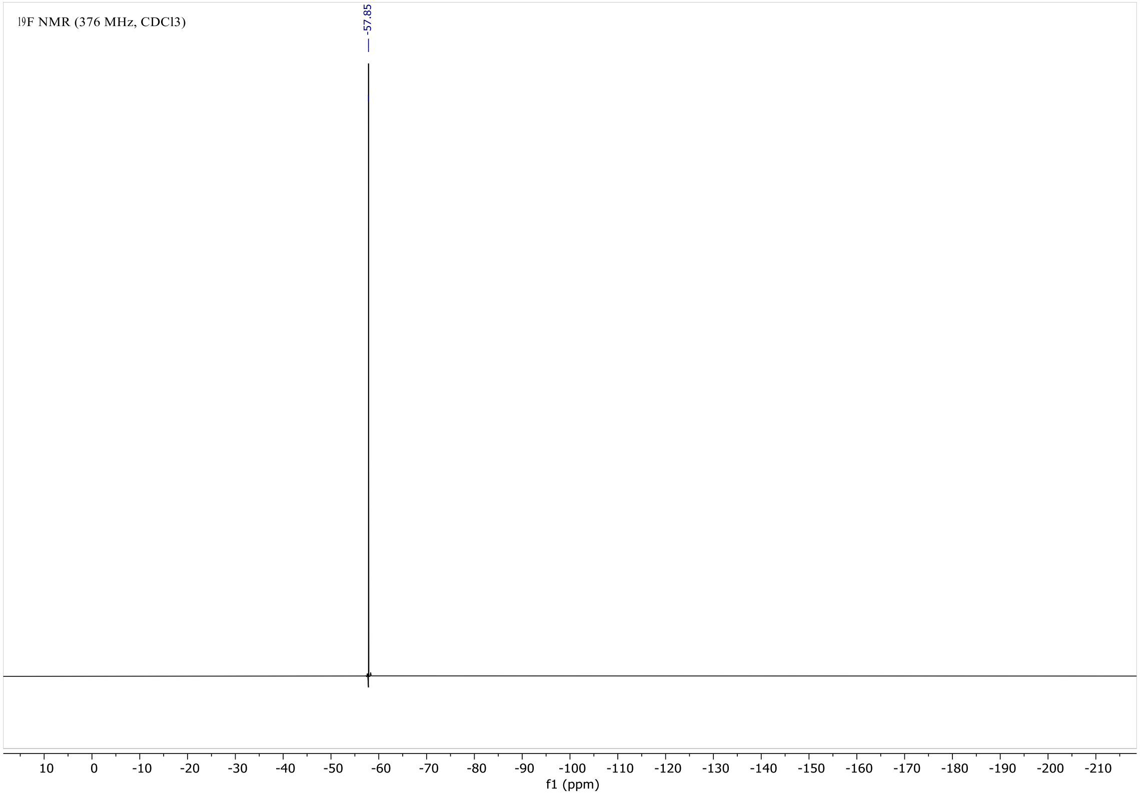

19F NMR (376 MHz, CDCl3)
-57.85
f1 (ppm)

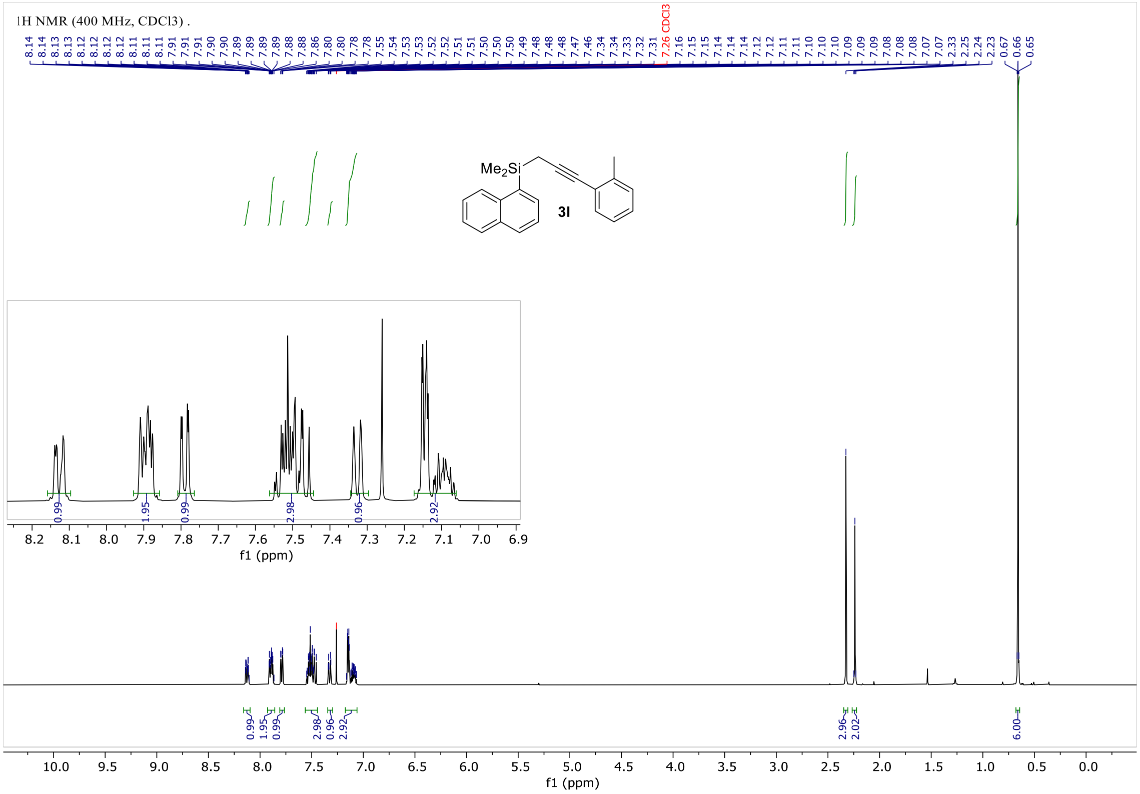
1H NMR (400 MHz, CDCl3) .
8.14 8.14 8.13 8.12 8.12 8.12 8.11 8.11 7.91 7.91 7.90 7.89 7.89 7.89 7.88 7.86 7.80 7.80 7.78 7.55 7.54 7.53 7.53 7.52 7.51 7.51 7.50 7.50 7.49 7.48 7.48 7.47 7.46 7.34 7.34 7.33 7.32 7.31 7.26 CDCl3 7.16 7.15 7.15 7.14 7.14 7.14 7.12 7.12 7.11 7.11 7.10 7.10 7.09 7.09 7.09 7.09 7.08 7.08 7.07 7.07 2.33 2.25 2.24 2.23 0.67 0.66 0.65
Me2Si
3l
0.99 1.95 0.99 2.98 0.96 2.92 2.96 2.02 6.00
f1 (ppm)

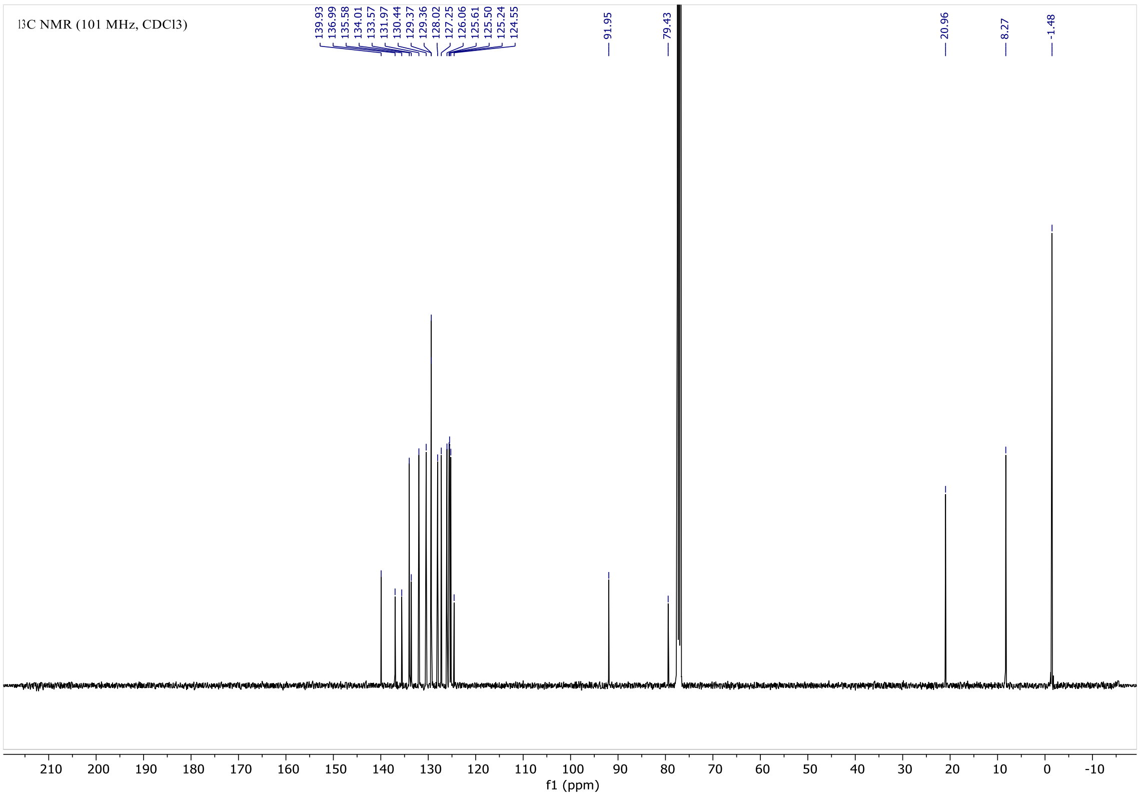
13C NMR (101 MHz, CDCl3)
139.93 136.99 135.58 134.01 133.57 131.97 130.44 129.37 129.36 128.02 127.25 126.06 125.61 125.50 125.24 124.55 91.95 79.43 20.96 8.27 -1.48
f1 (ppm)

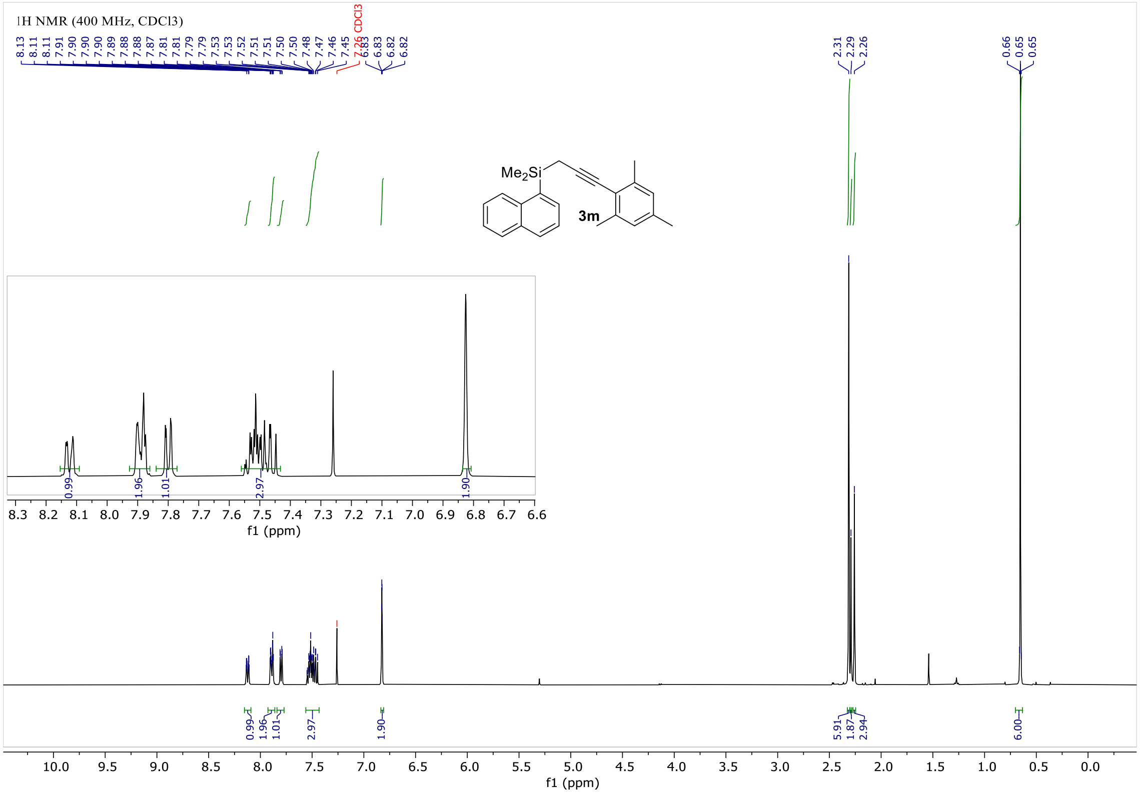
1H NMR (400 MHz, CDCl3)
Me2Si
3m
f1 (ppm)

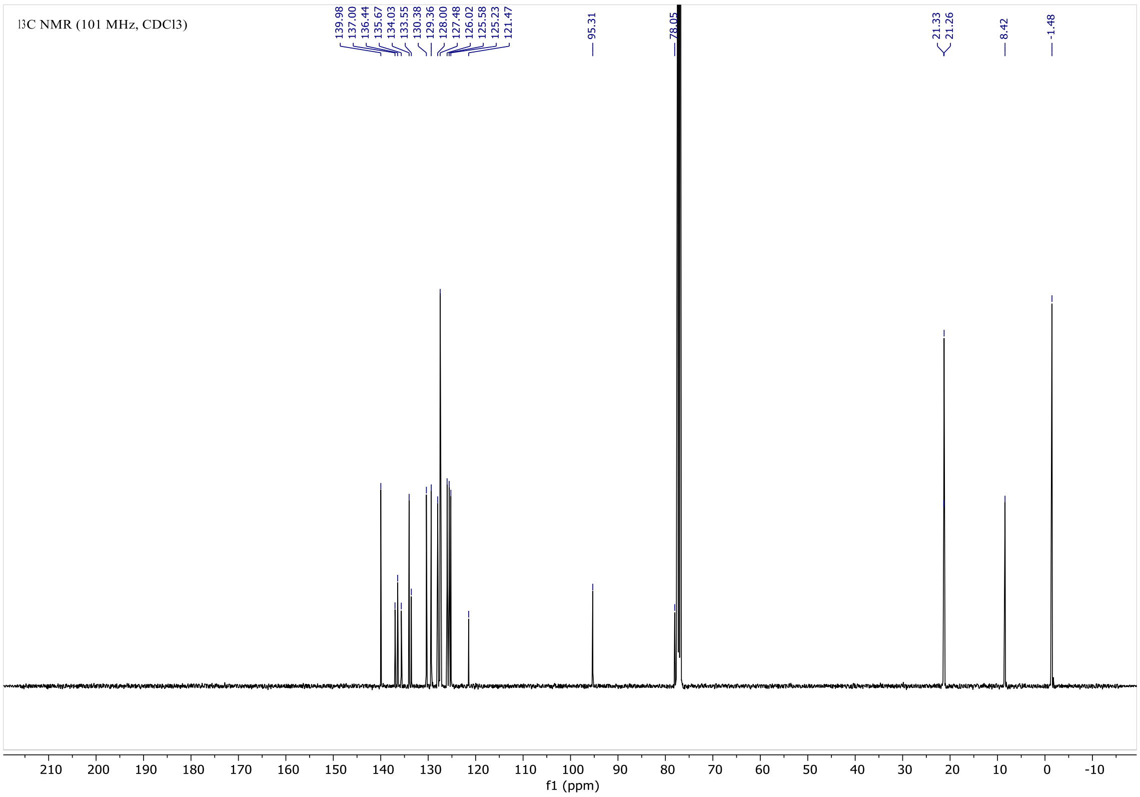
13C NMR (101 MHz, CDCl3)
f1 (ppm)

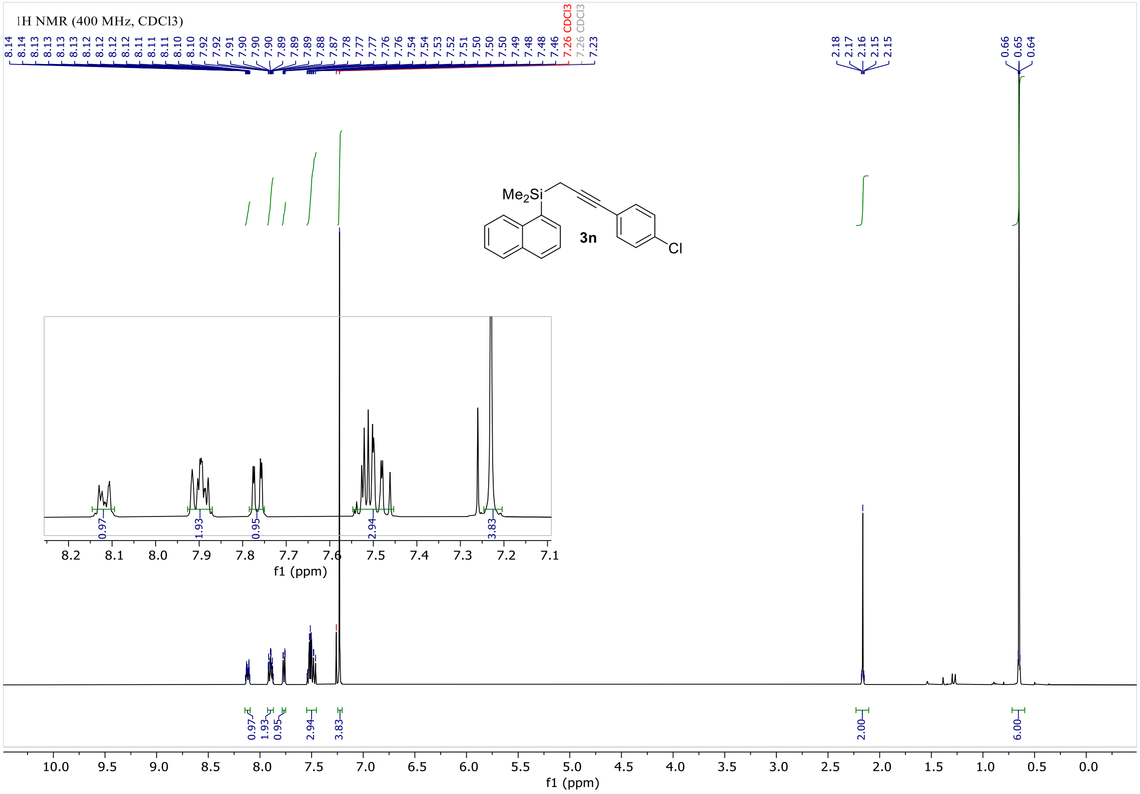
1H NMR (400 MHz, CDCl3)
Me2Si
3n
Cl
f1 (ppm)

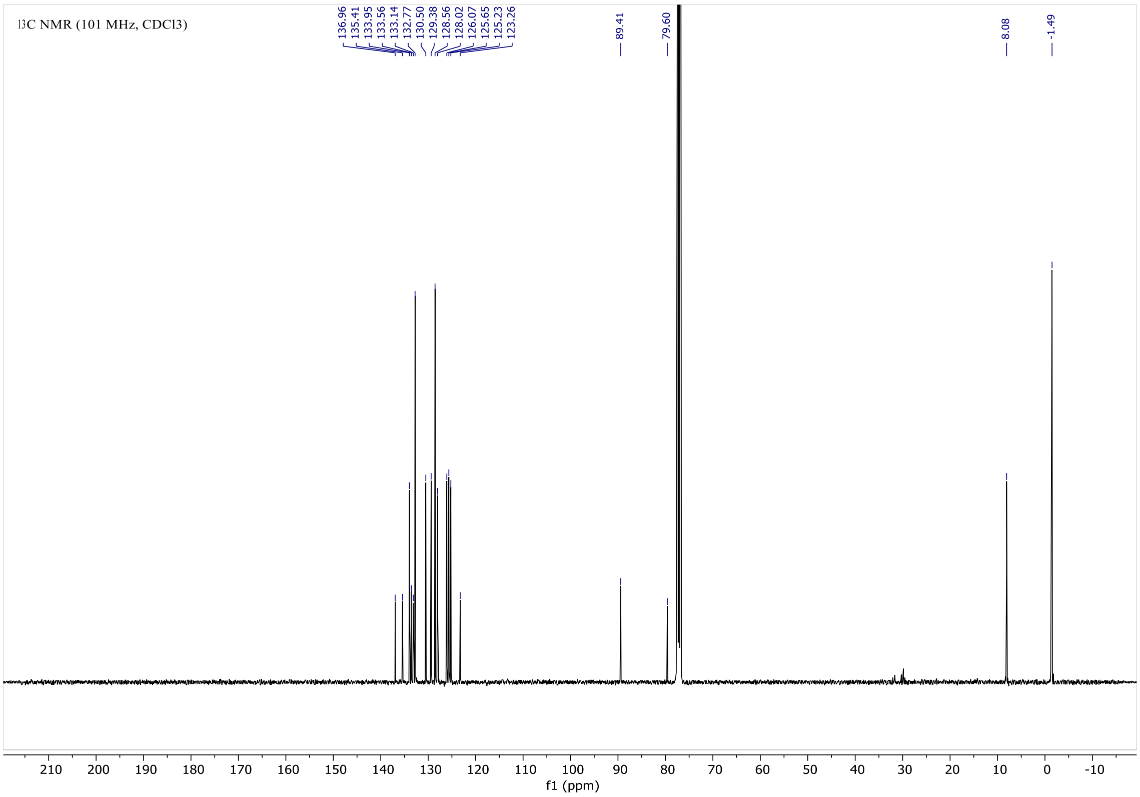
13C NMR (101 MHz, CDCl3)
f1 (ppm)

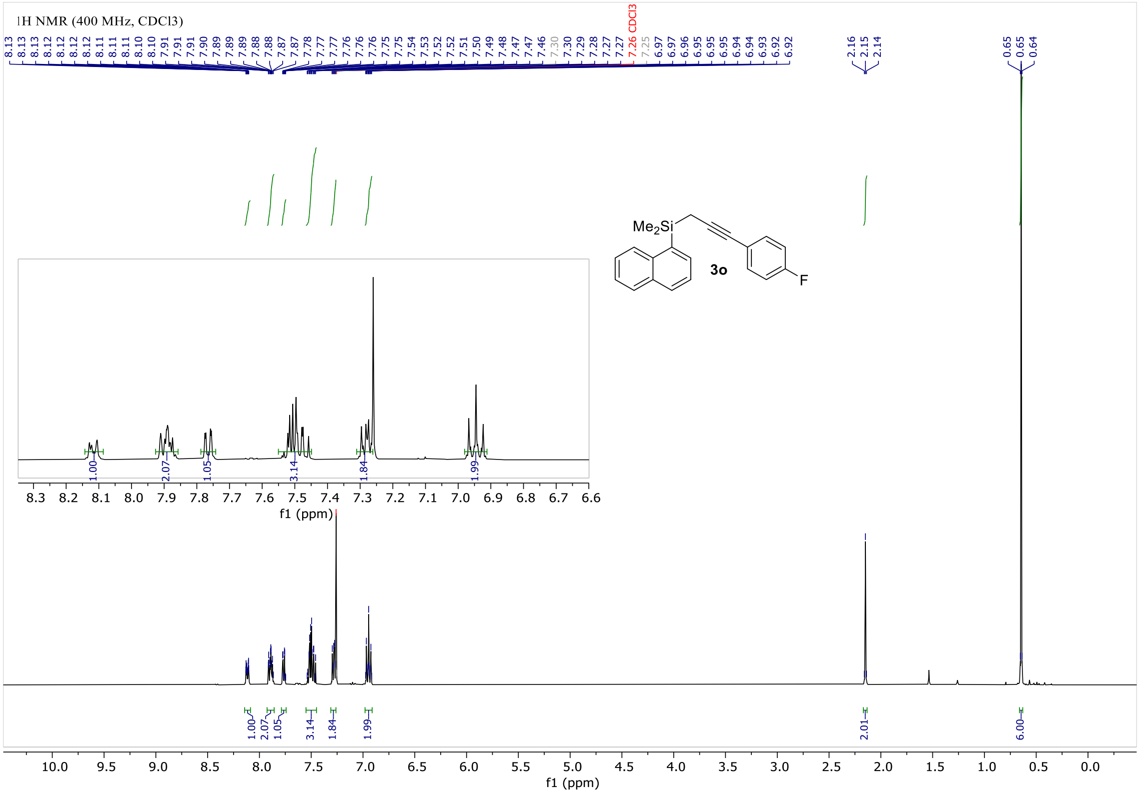
1H NMR (400 MHz, CDCl3)
Me2Si
3o
F
f1 (ppm)

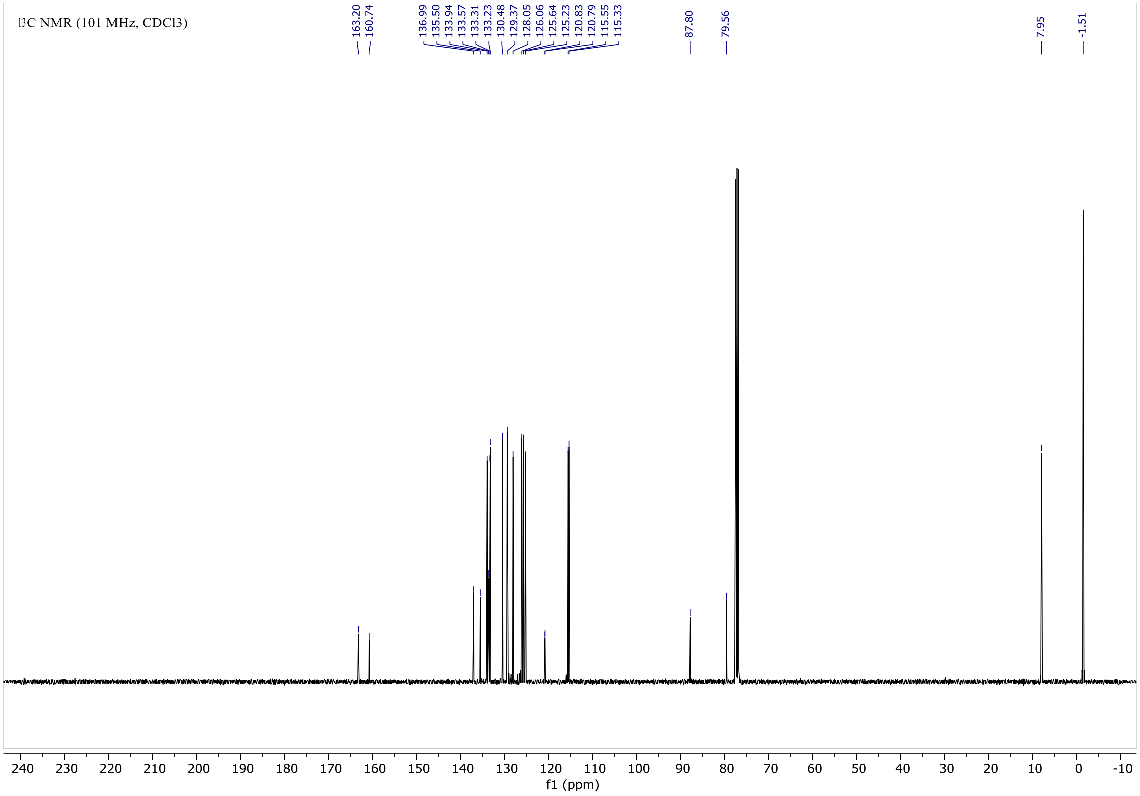
13C NMR (101 MHz, CDCl3)
163.20
160.74
136.99
135.50
133.94
133.57
133.31
133.23
130.48
129.37
128.05
126.06
125.64
125.23
120.83
120.79
115.55
115.33
87.80
79.56
7.95
-1.51
f1 (ppm)

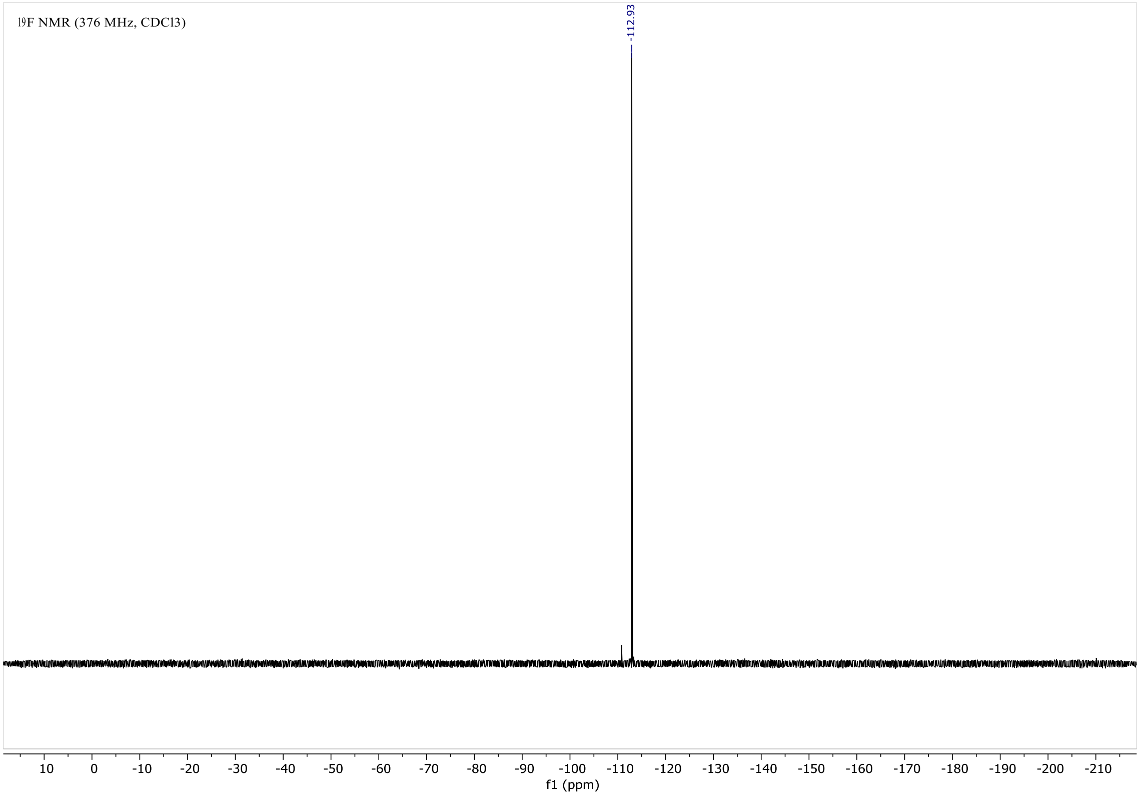
19F NMR (376 MHz, CDCl3)
-112.93
f1 (ppm)

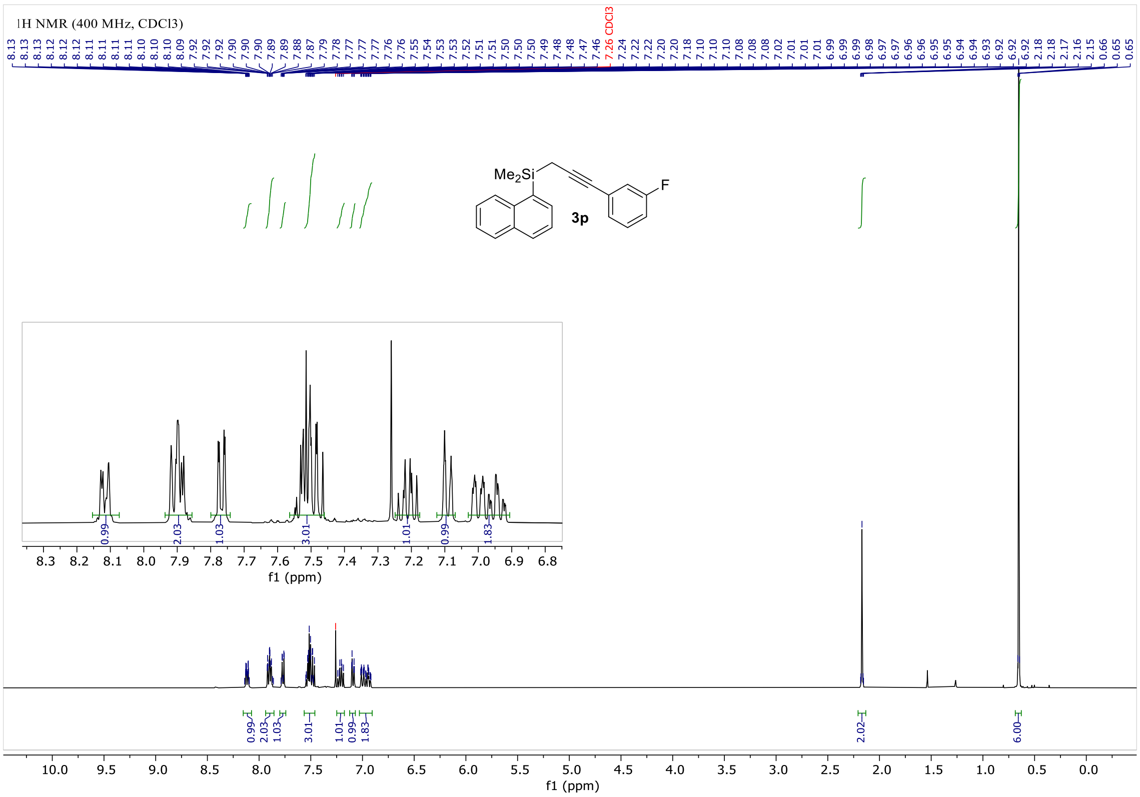
1H NMR (400 MHz, CDCl3)
7.26 CDCl3
Me2Si
F
3p
0.99
2.03
1.03
3.01
1.01
0.99
1.83
2.02
6.00
f1 (ppm)

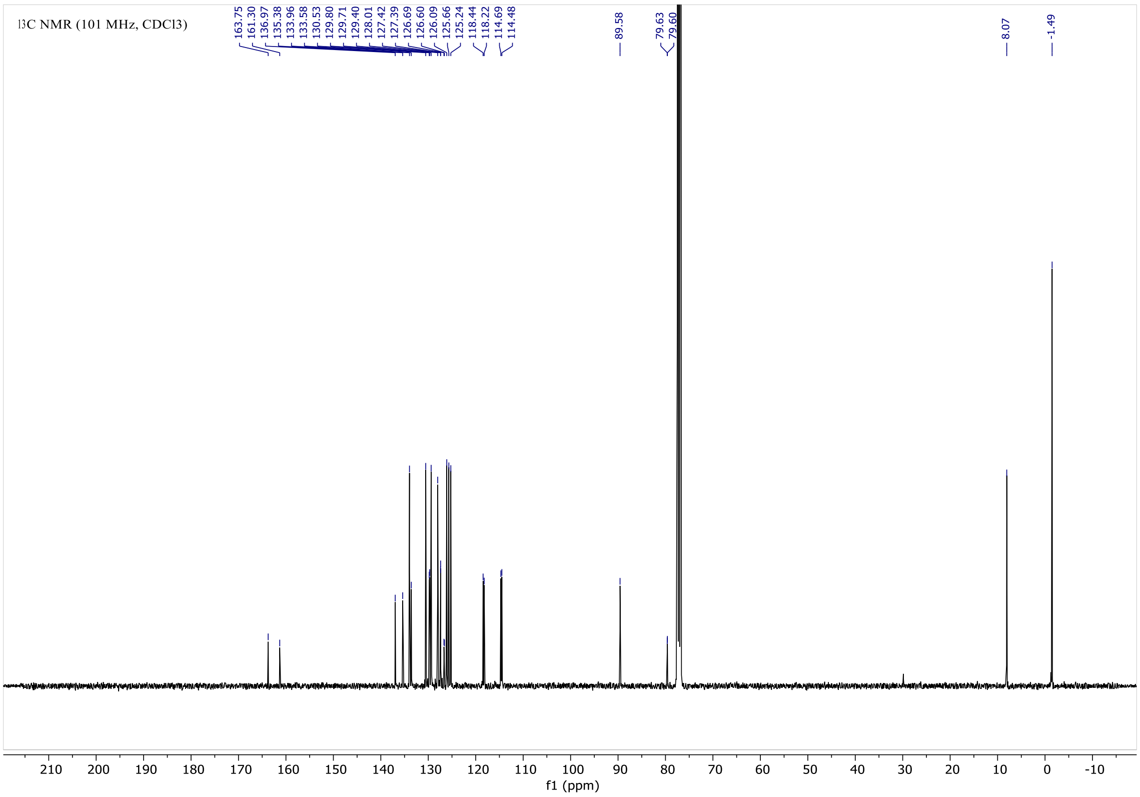
13C NMR (101 MHz, CDCl3)
163.75
161.30
136.97
135.38
133.96
133.58
130.53
129.80
129.71
129.40
128.01
127.42
127.39
126.69
126.60
126.09
125.66
125.24
118.44
118.22
114.69
114.48
89.58
79.63
79.60
8.07
-1.49
210 200 190 180 170 160 150 140 130 120 110 100 90 80 70 60 50 40 30 20 10 0 -10
f1 (ppm)

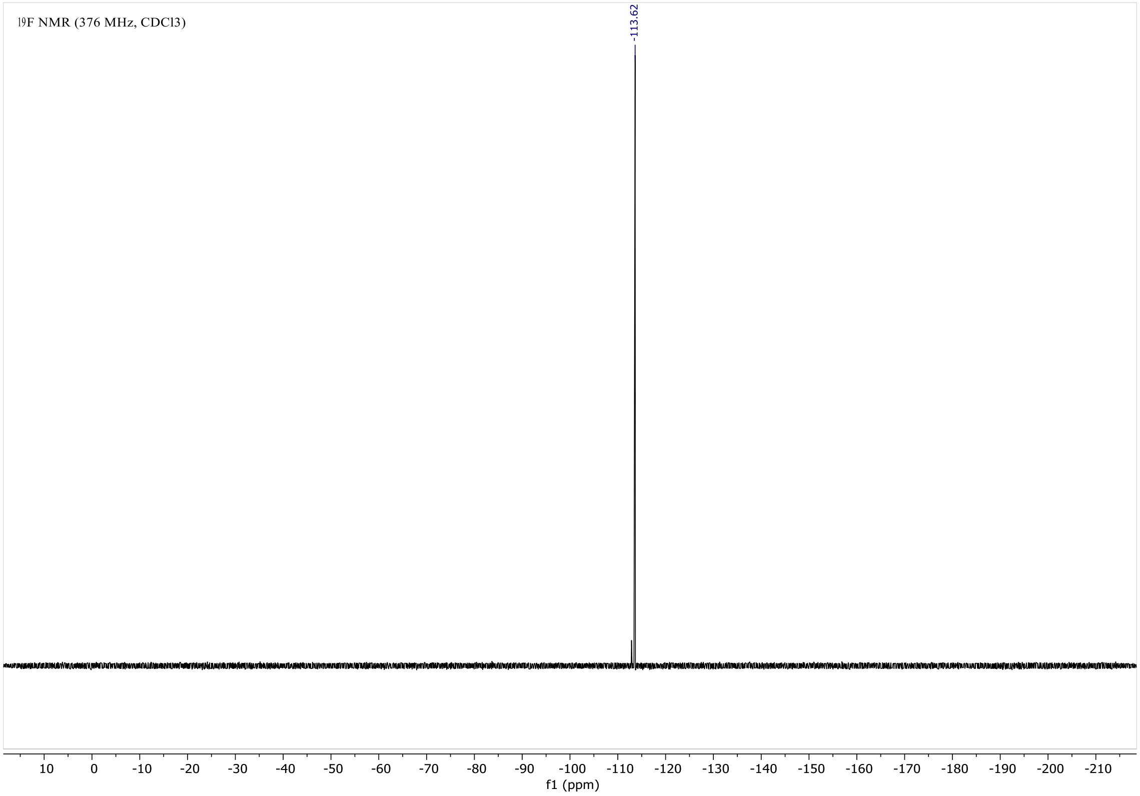
19F NMR (376 MHz, CDCl3)
-113.62
10 0 -10 -20 -30 -40 -50 -60 -70 -80 -90 -100 -110 -120 -130 -140 -150 -160 -170 -180 -190 -200 -210
f1 (ppm)

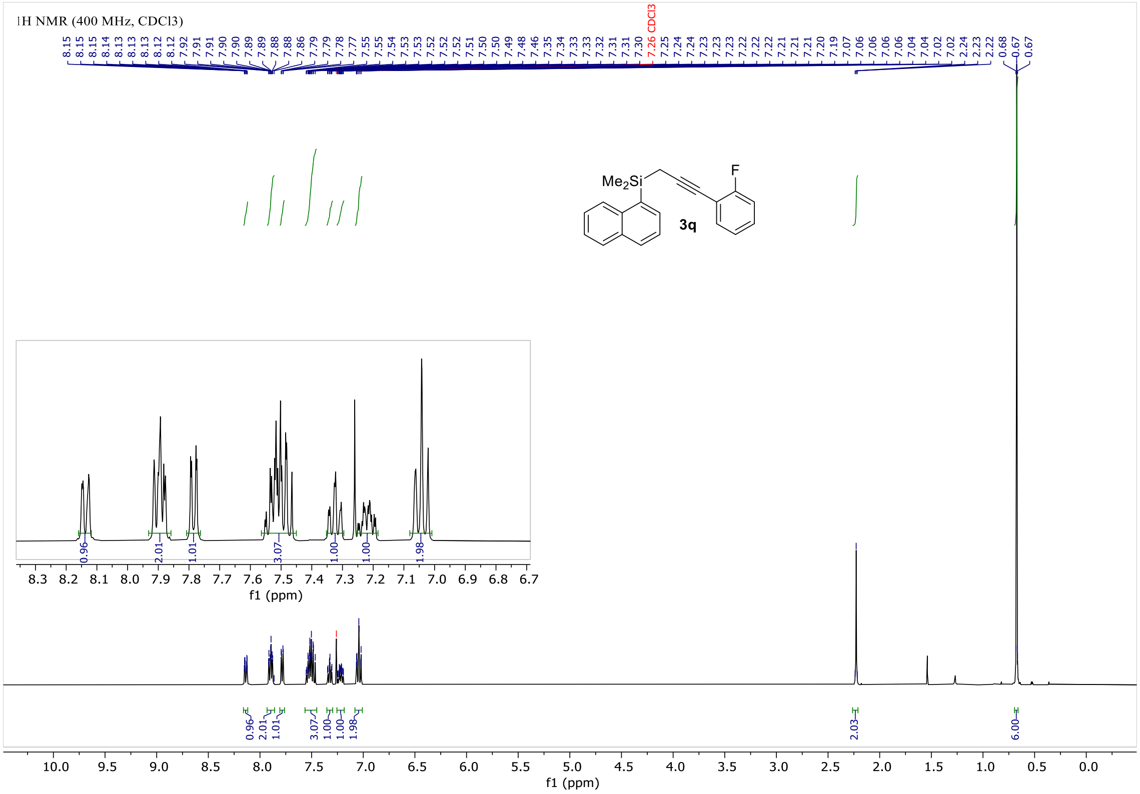
1H NMR (400 MHz, CDCl3)
8.15
8.15
8.14
8.13
8.13
8.13
8.12
8.12
7.92
7.91
7.91
7.90
7.90
7.89
7.89
7.88
7.88
7.86
7.79
7.79
7.78
7.77
7.55
7.55
7.54
7.53
7.53
7.52
7.52
7.51
7.50
7.50
7.49
7.48
7.46
7.35
7.34
7.33
7.33
7.32
7.31
7.31
7.30
7.26 CDCl3
7.25
7.24
7.24
7.23
7.23
7.22
7.22
7.22
7.21
7.21
7.21
7.20
7.19
7.07
7.06
7.06
7.06
7.06
7.04
7.04
7.02
7.02
2.24
2.23
2.22
0.68
0.67
0.67
Me2Si
F
3q
0.96
2.01
1.01
3.07
1.00
1.00
1.98
2.03
6.00
f1 (ppm)

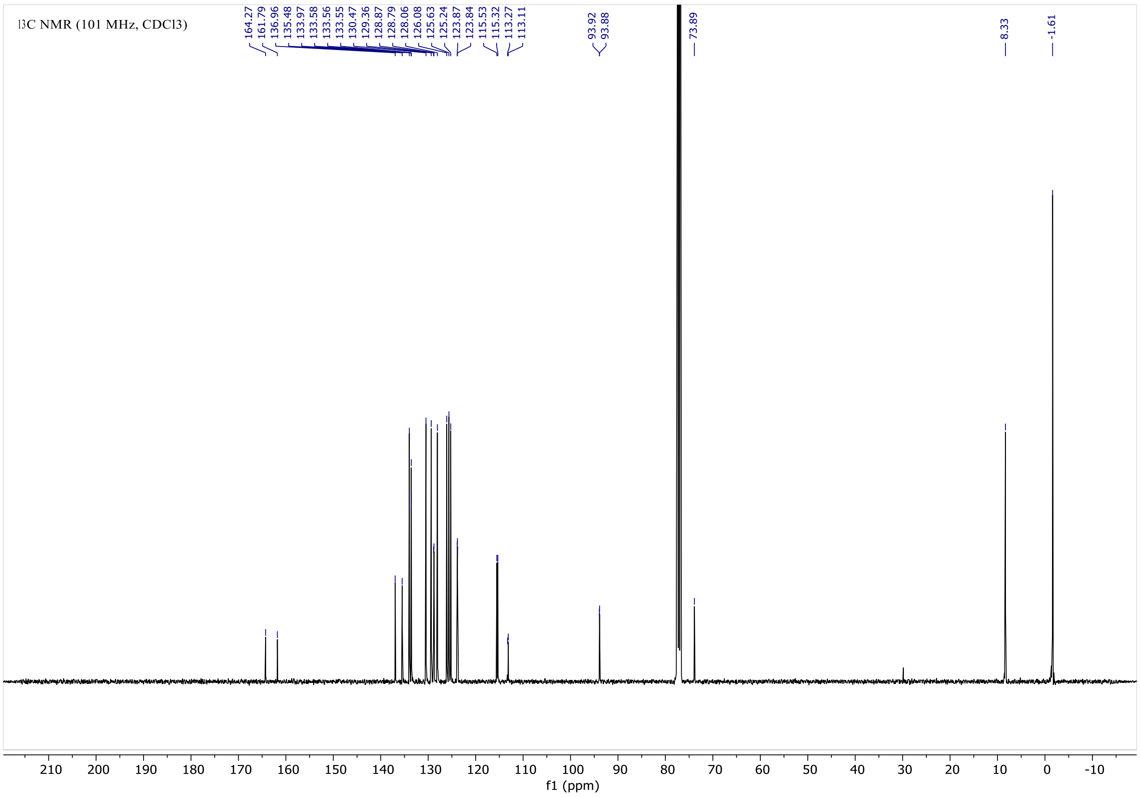
13C NMR (101 MHz, CDCl3)
164.27
161.79
136.96
135.48
133.97
133.58
133.56
133.55
130.47
129.36
128.87
128.79
128.06
126.08
125.63
125.24
123.87
123.84
115.53
115.32
113.27
113.11
93.92
93.88
73.89
8.33
-1.61
f1 (ppm)

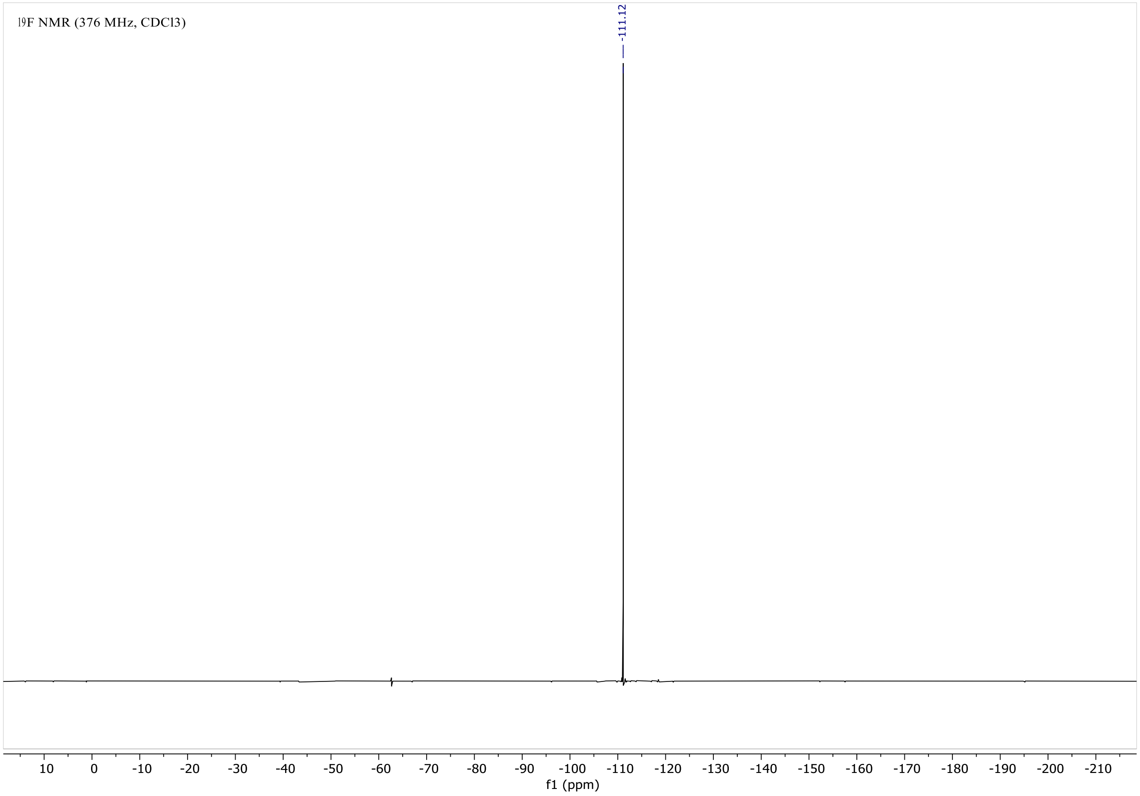
19F NMR (376 MHz, CDCl3)
-111.12
f1 (ppm)

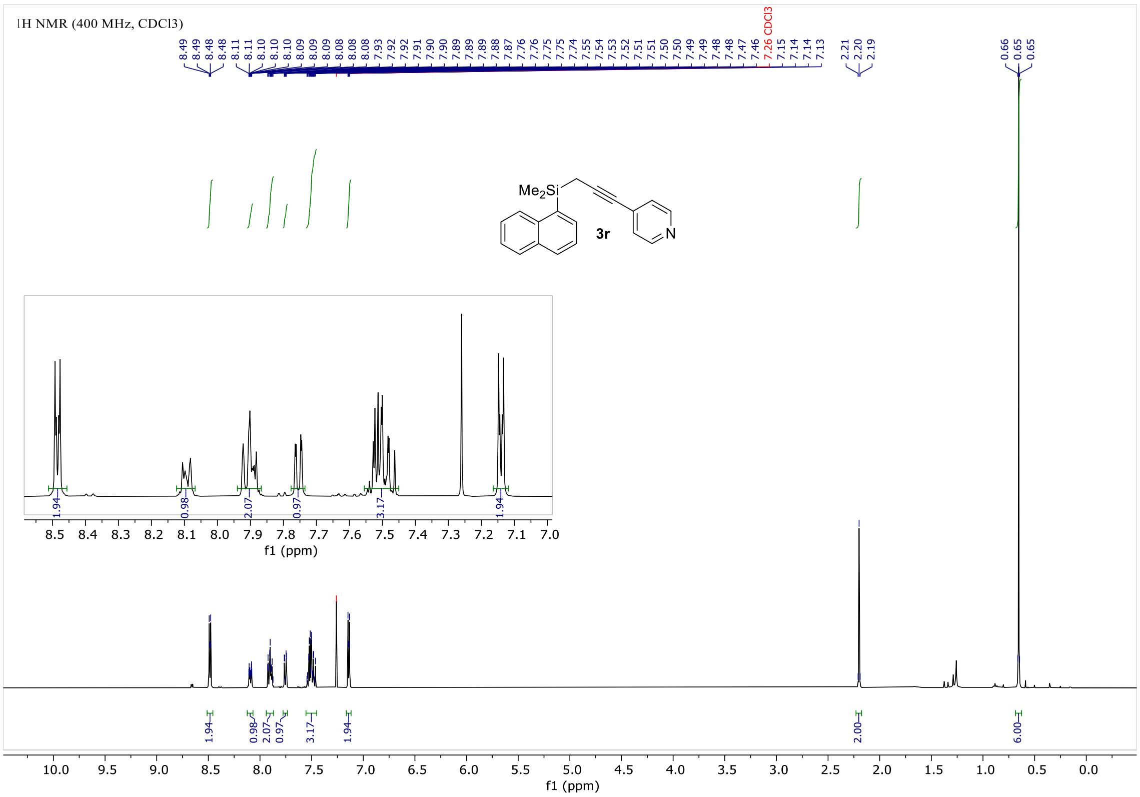
1H NMR (400 MHz, CDCl3)
Me2Si
N
3r
f1 (ppm)

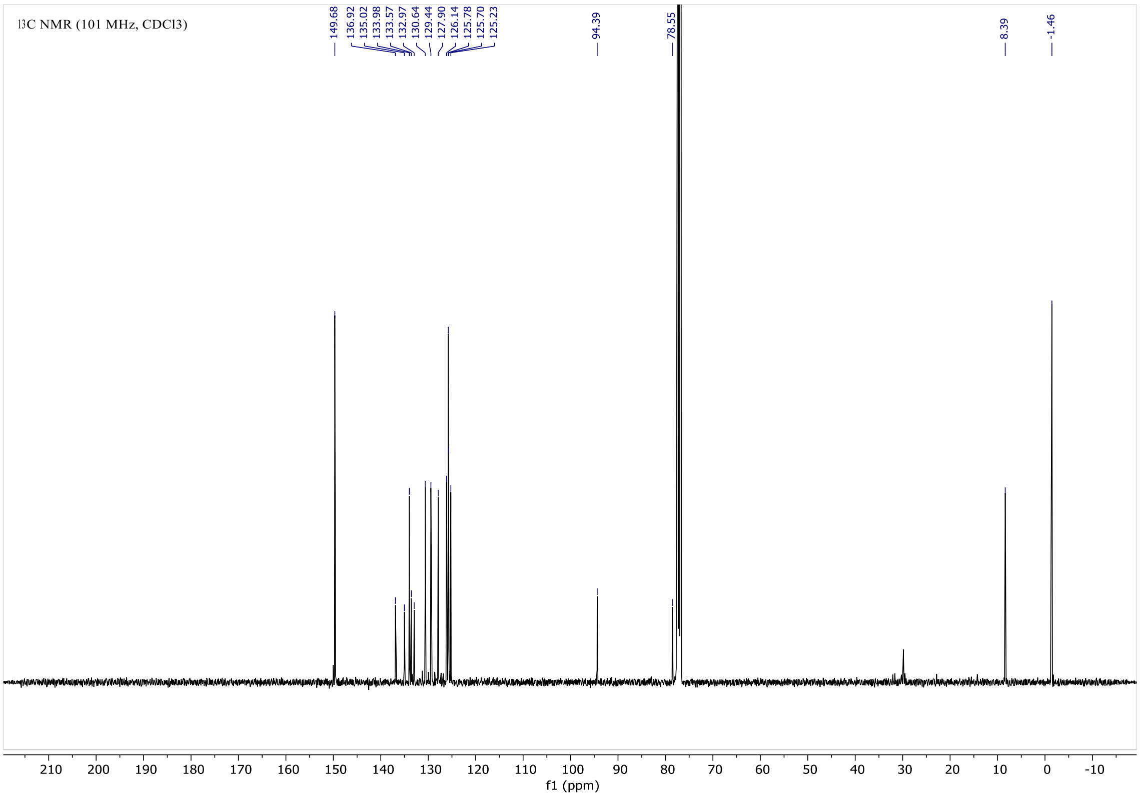
13C NMR (101 MHz, CDCl3)
149.68
136.92
135.02
133.98
133.57
132.97
130.64
129.44
127.90
126.14
125.78
125.70
125.23
94.39
78.55
8.39
-1.46
f1 (ppm)

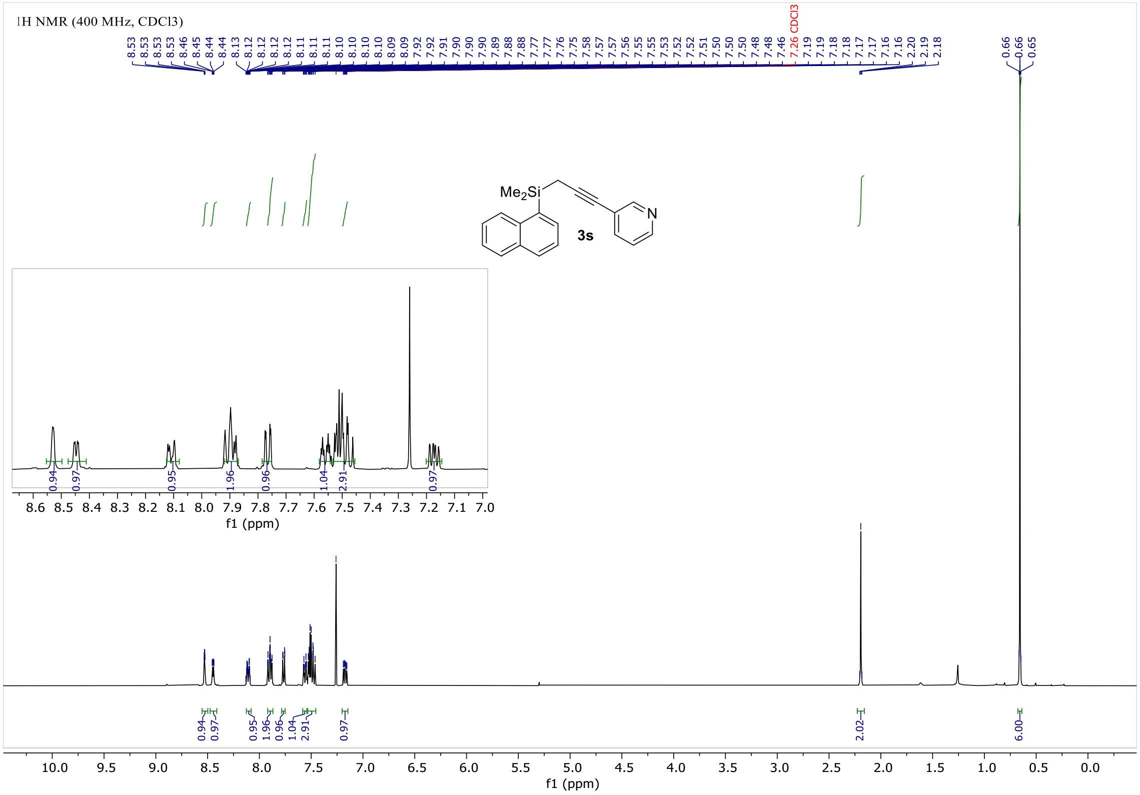
1H NMR (400 MHz, CDCl3)
7.26 CDCl3
Me_2Si
N
3s
f1 (ppm)

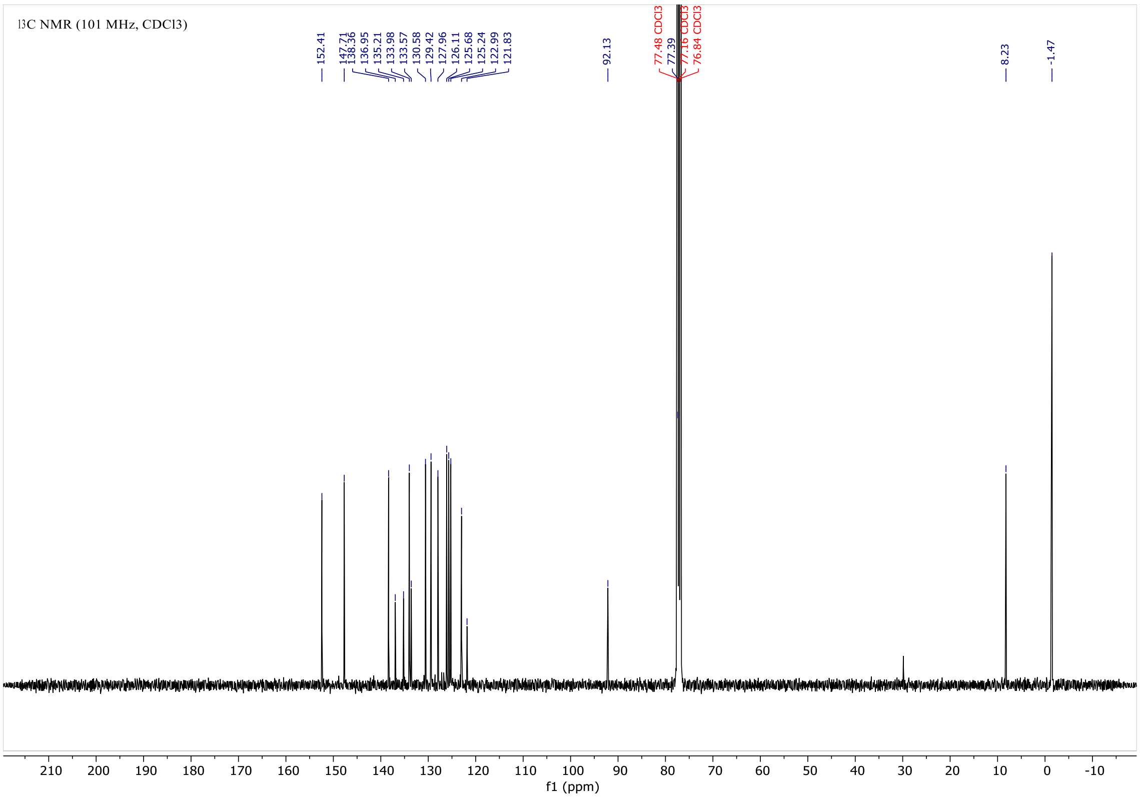
13C NMR (101 MHz, CDCl3)
152.41
147.71
138.36
136.95
135.21
133.98
133.57
130.58
129.42
127.96
126.11
125.68
125.24
122.99
121.83
92.13
77.48 CDCl3
77.39
77.16 CDCl3
76.84 CDCl3
8.23
-1.47
f1 (ppm)

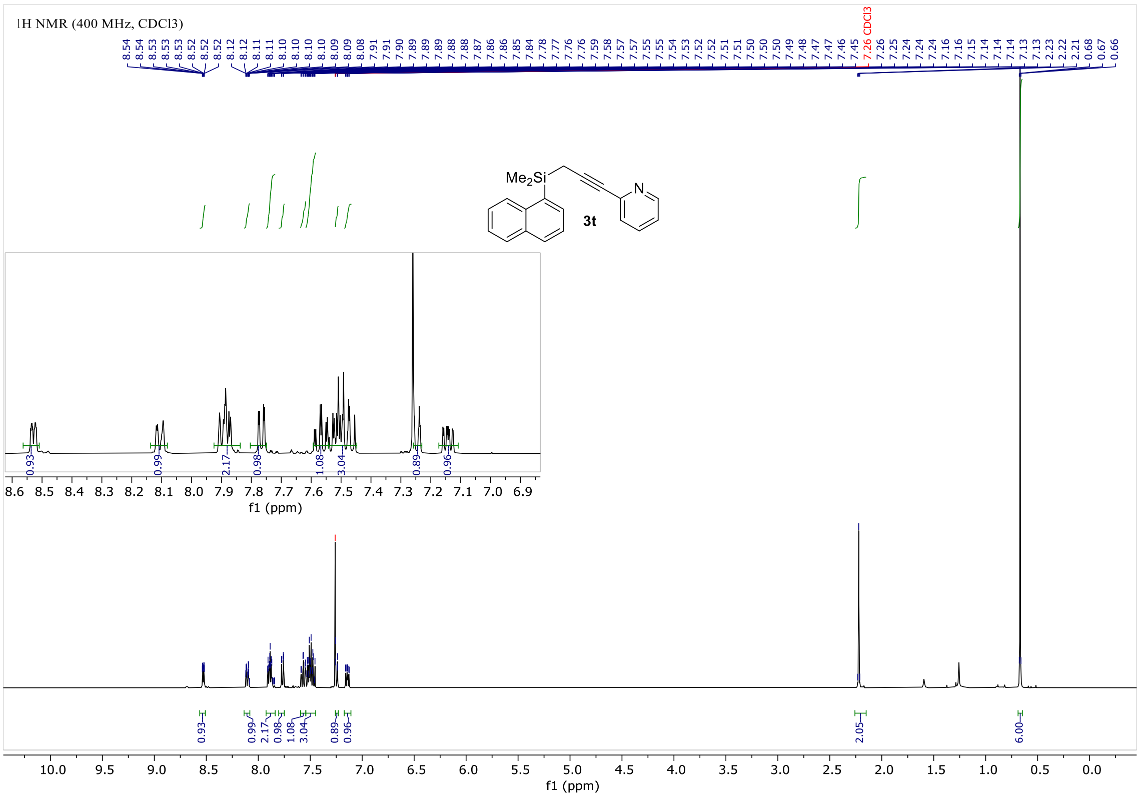
1H NMR (400 MHz, CDCl3)
8.54 8.54 8.53 8.53 8.52 8.52 8.52 8.12 8.11 8.11 8.10 8.10 8.10 8.09 8.09 8.08 7.91 7.91 7.90 7.89 7.89 7.88 7.88 7.87 7.86 7.86 7.85 7.84 7.78 7.77 7.76 7.76 7.59 7.58 7.57 7.57 7.55 7.54 7.53 7.52 7.52 7.51 7.51 7.50 7.50 7.49 7.48 7.47 7.46 7.45
7.26 CDCl3
7.25 7.24 7.24 7.24 7.16 7.16 7.15 7.14 7.14 7.14 7.13 7.13 2.23 2.22 2.21 0.68 0.67 0.66
Me2Si
N
3t
0.93
0.99
2.17
0.98
1.08
3.04
0.89
0.96
2.05
6.00
f1 (ppm)

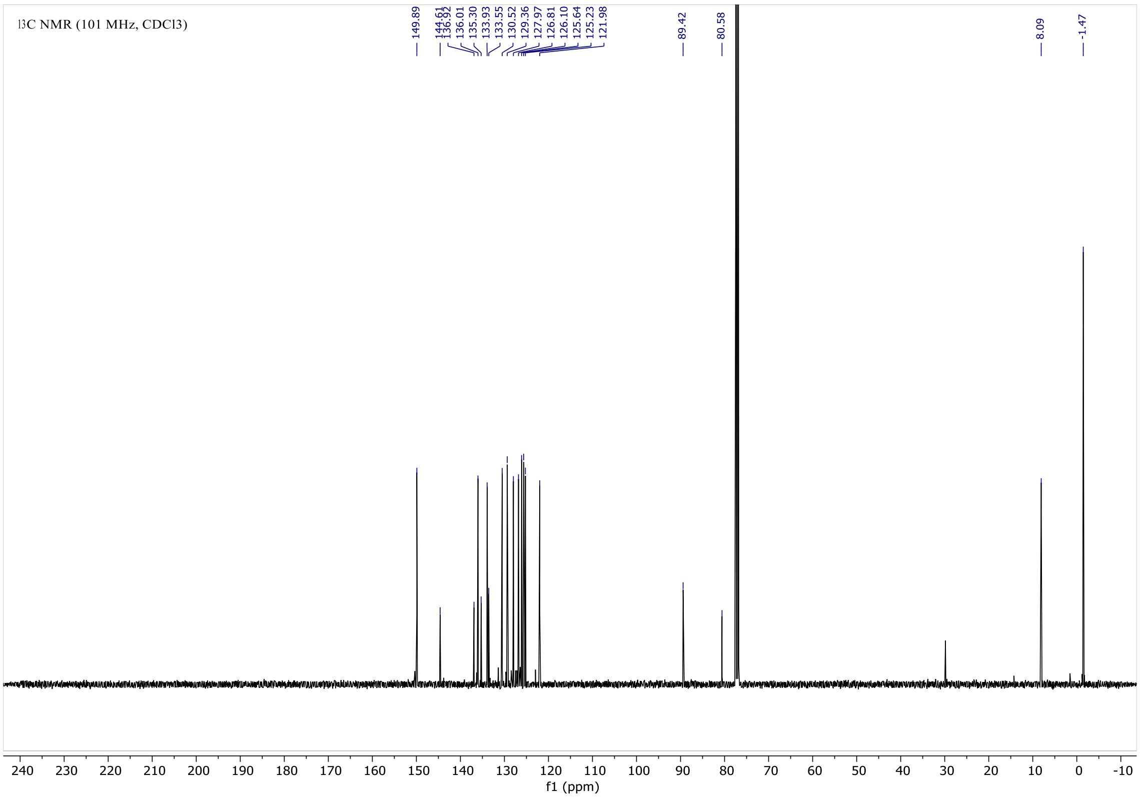
13C NMR (101 MHz, CDCl3)
149.89
144.61
136.82
136.01
135.30
133.93
133.55
130.52
129.36
127.97
126.81
126.10
125.64
125.23
121.98
89.42
80.58
8.09
-1.47
f1 (ppm)

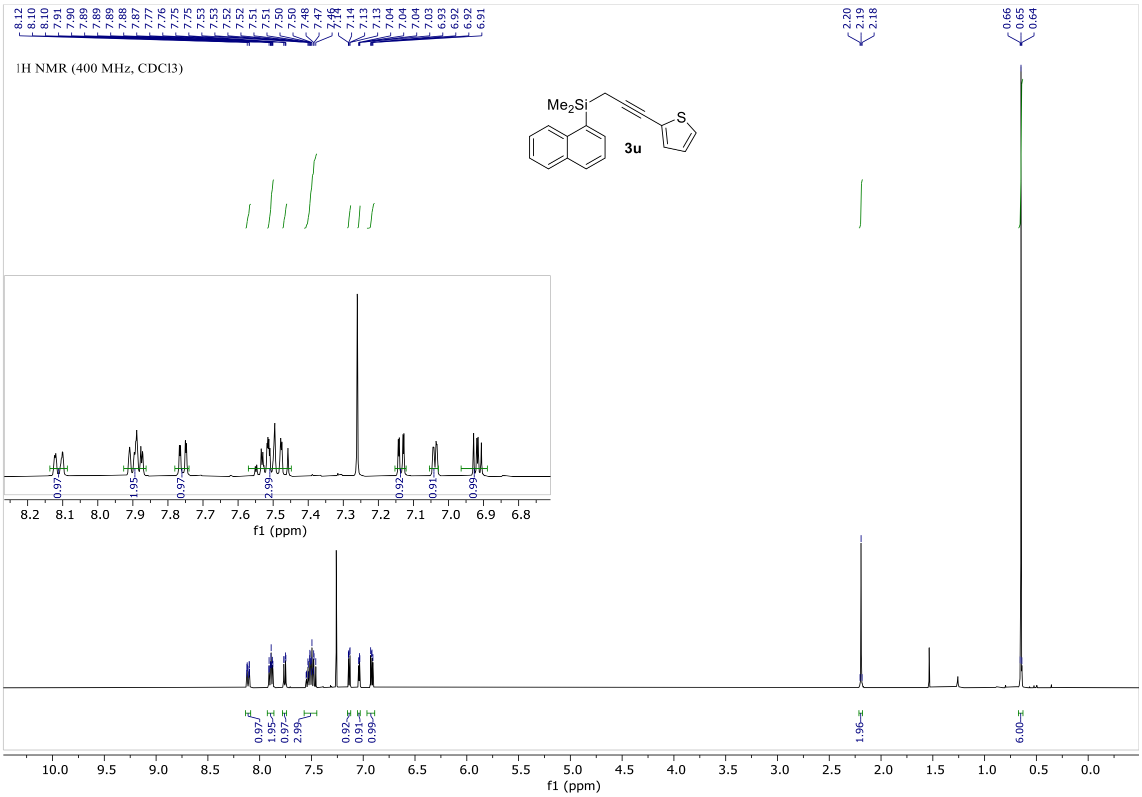
1H NMR (400 MHz, CDCl3)
Me2Si
S
3u
2.20
2.19
2.18
0.66
0.65
0.64
0.97
1.95
0.97
2.99
0.92
0.91
0.99
1.96
6.00
f1 (ppm)

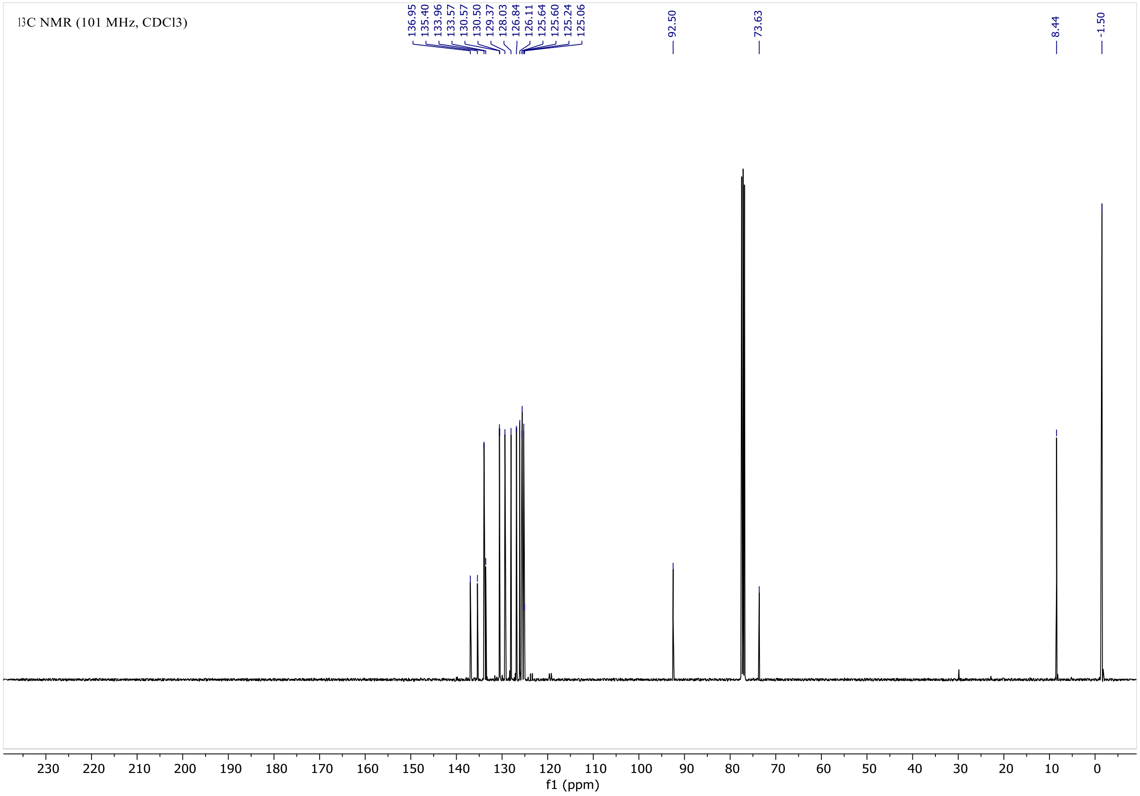
13C NMR (101 MHz, CDCl3)
136.95
135.40
133.96
133.57
130.57
130.50
129.37
128.03
126.84
126.11
125.64
125.60
125.24
125.06
92.50
73.63
8.44
-1.50
f1 (ppm)

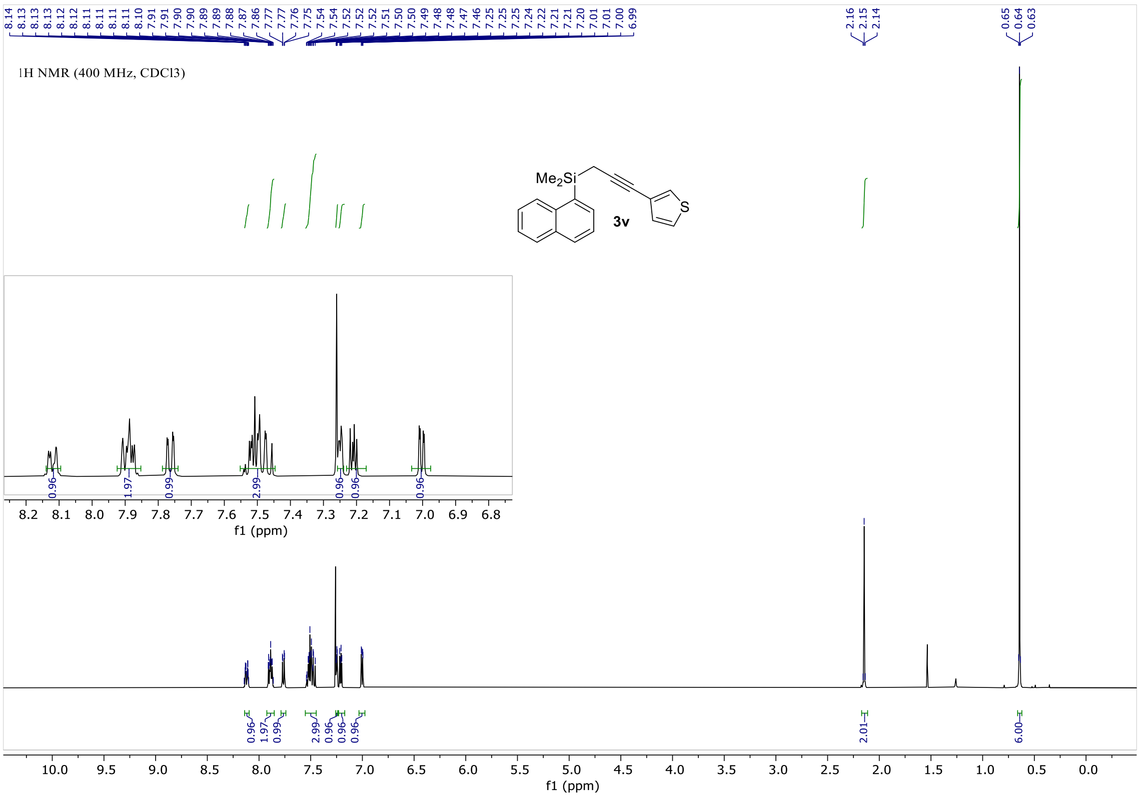
1H NMR (400 MHz, CDCl3)
Me2Si
S
3v
f1 (ppm)

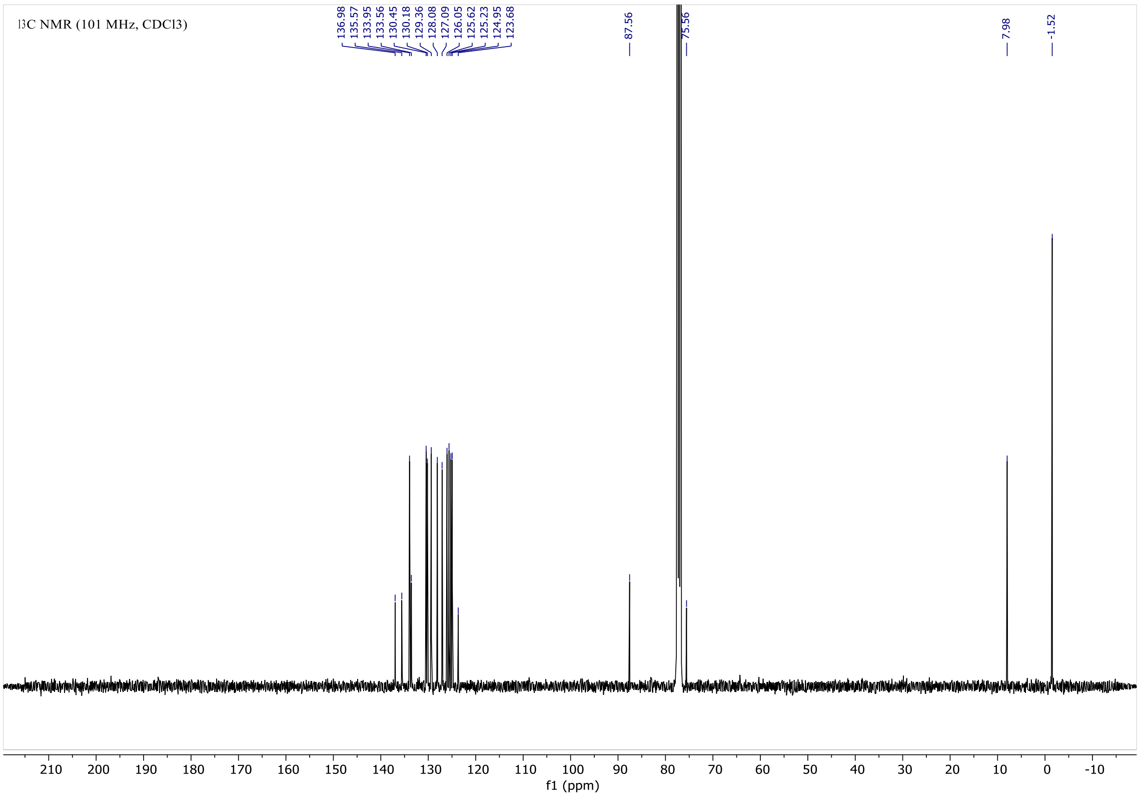
13C NMR (101 MHz, CDCl3)
136.98
135.57
133.95
133.56
130.45
130.18
129.36
128.08
127.09
126.05
125.62
125.23
124.95
123.68
87.56
75.56
7.98
-1.52
f1 (ppm)

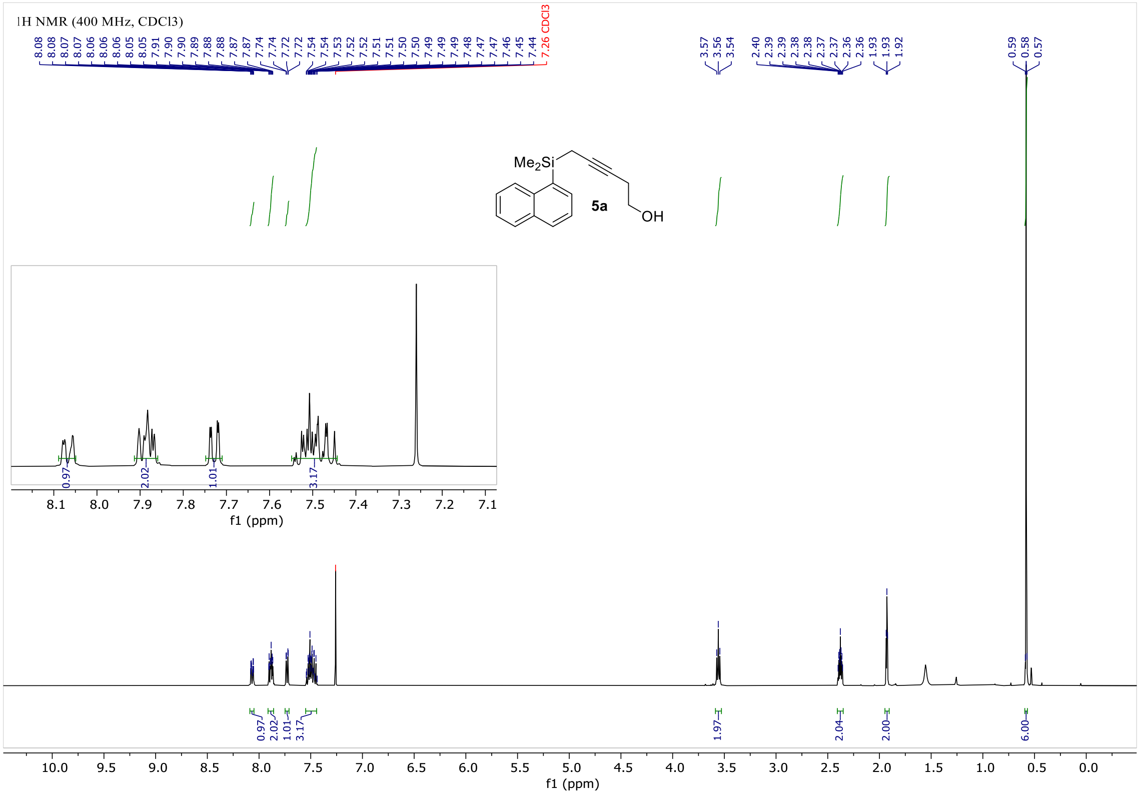
1H NMR (400 MHz, CDCl3)
7.26 CDCl3
3.57
3.56
3.54
0.59
0.58
0.57
Me2Si
5a
OH
0.97
2.02
1.01
3.17
1.97
2.04
2.00
6.00
f1 (ppm)

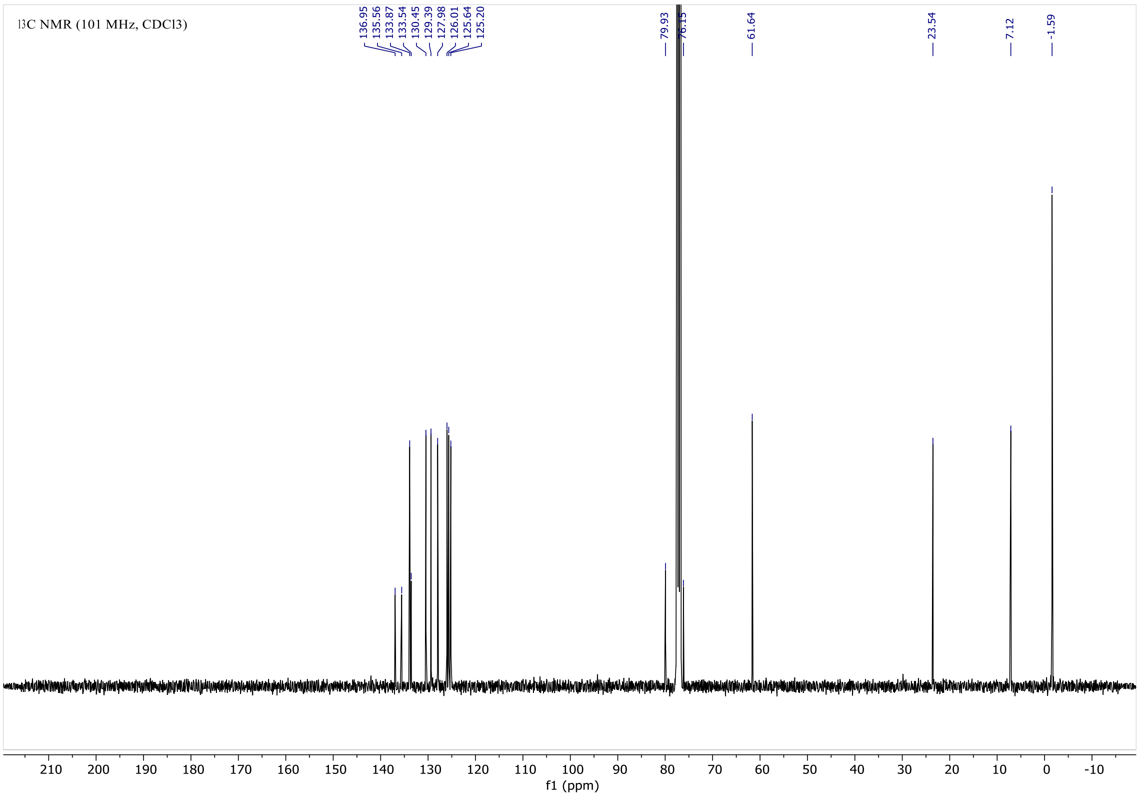
13C NMR (101 MHz, CDCl3)
136.95
135.56
133.87
133.54
130.45
129.39
127.98
126.01
125.64
125.20
79.93
76.15
61.64
23.54
7.12
-1.59
f1 (ppm)

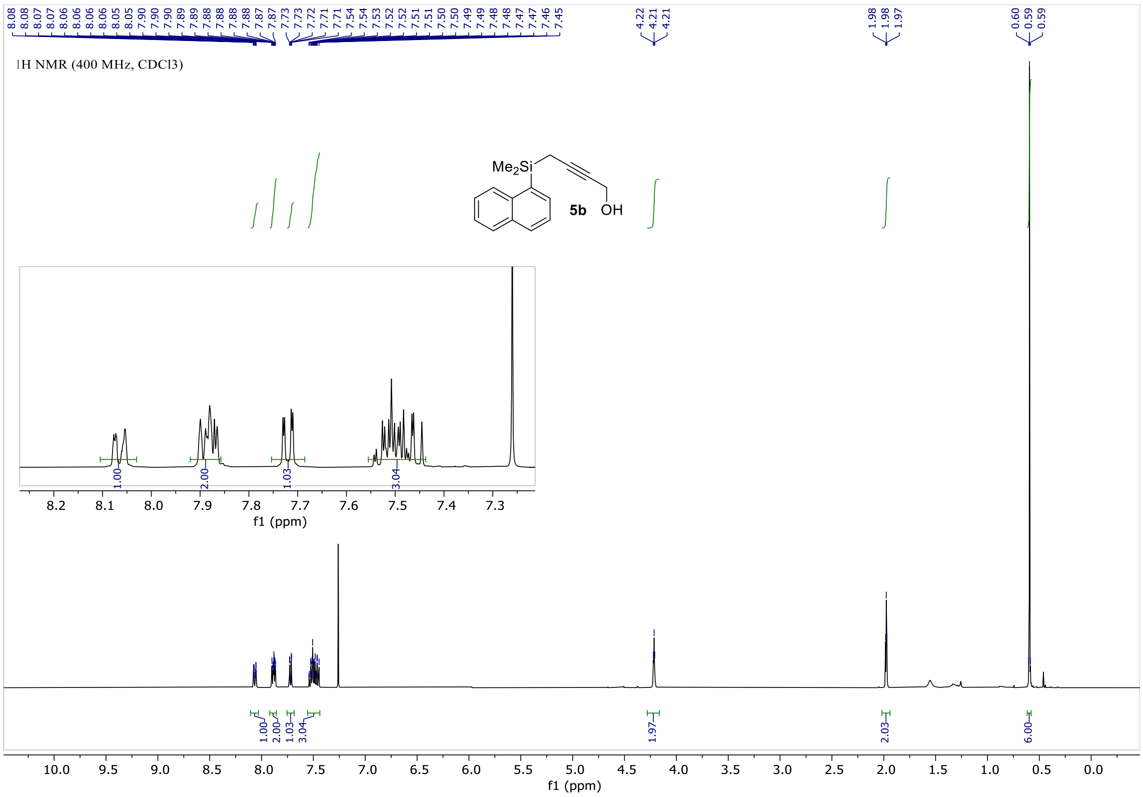
1H NMR (400 MHz, CDCl3)
Me2Si
5b
OH
4.22
4.21
4.21
1.98
1.98
1.97
0.60
0.59
0.59
1.00
2.00
1.03
3.04
1.97
2.03
6.00
f1 (ppm)

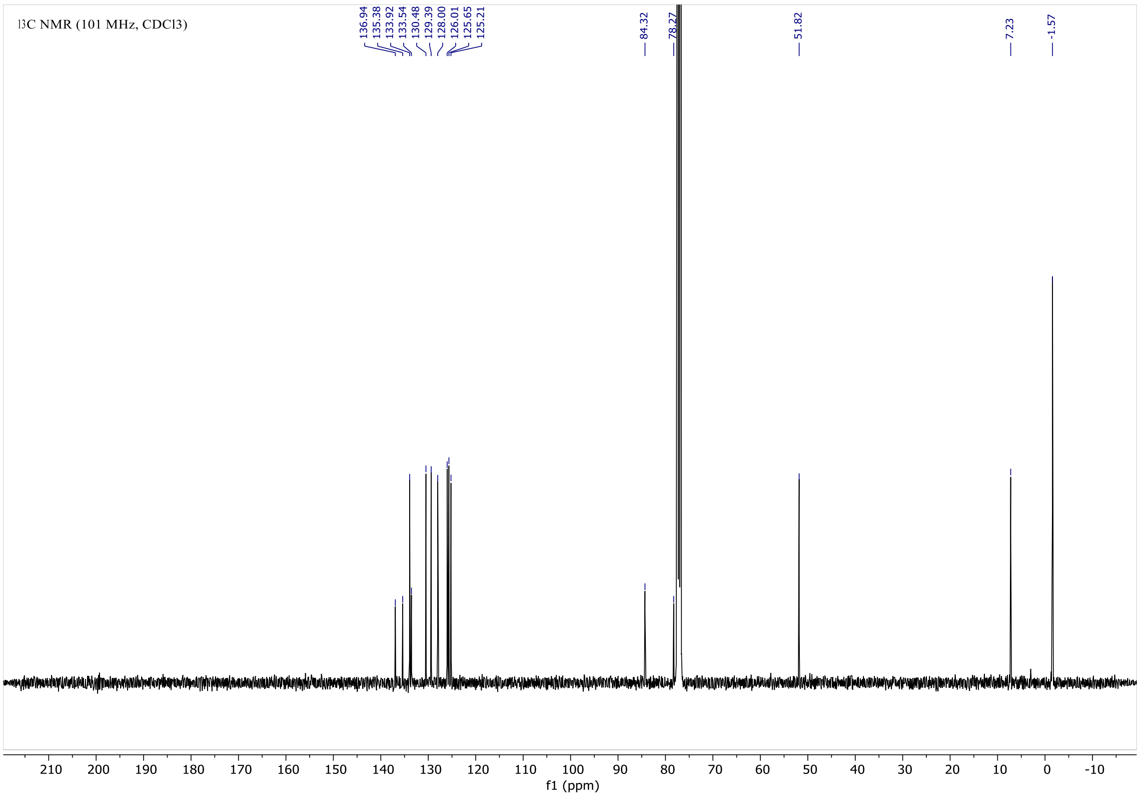
13C NMR (101 MHz, CDCl3)
136.94
135.38
133.92
133.54
130.48
129.39
128.00
126.01
125.65
125.21
84.32
78.27
51.82
7.23
-1.57
f1 (ppm)

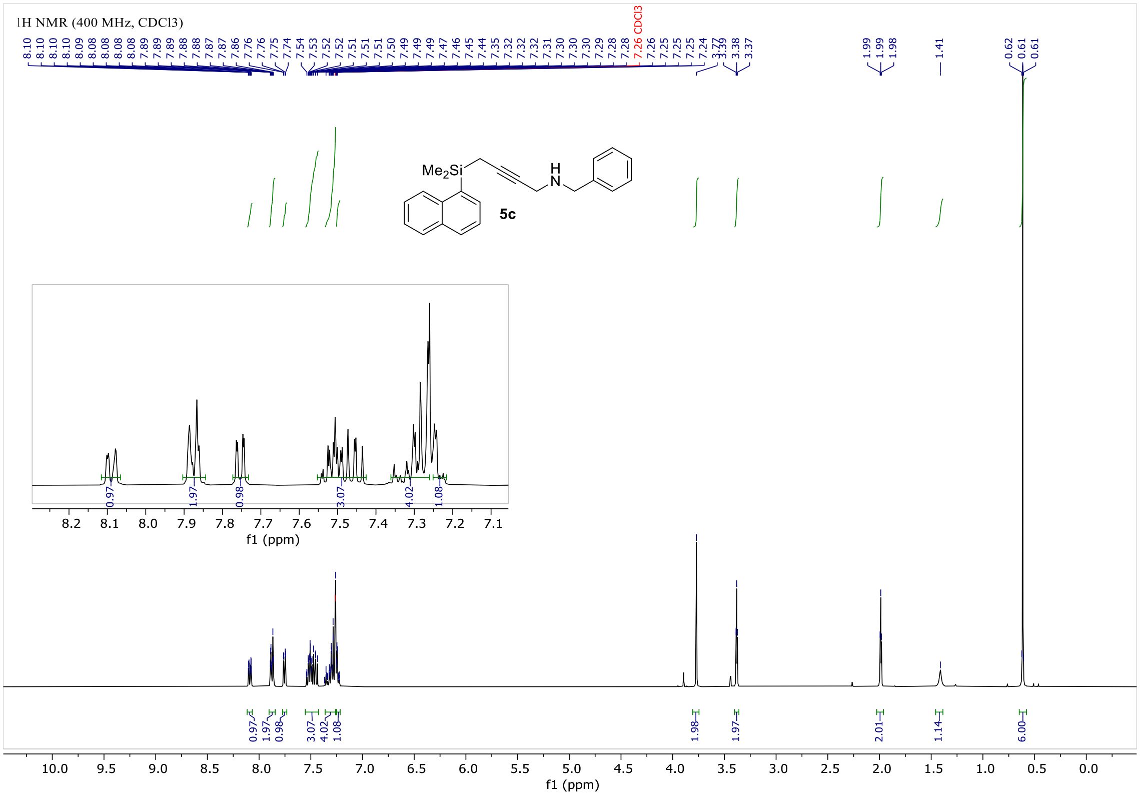
1H NMR (400 MHz, CDCl3)
Me2Si
H
N
5c
f1 (ppm)

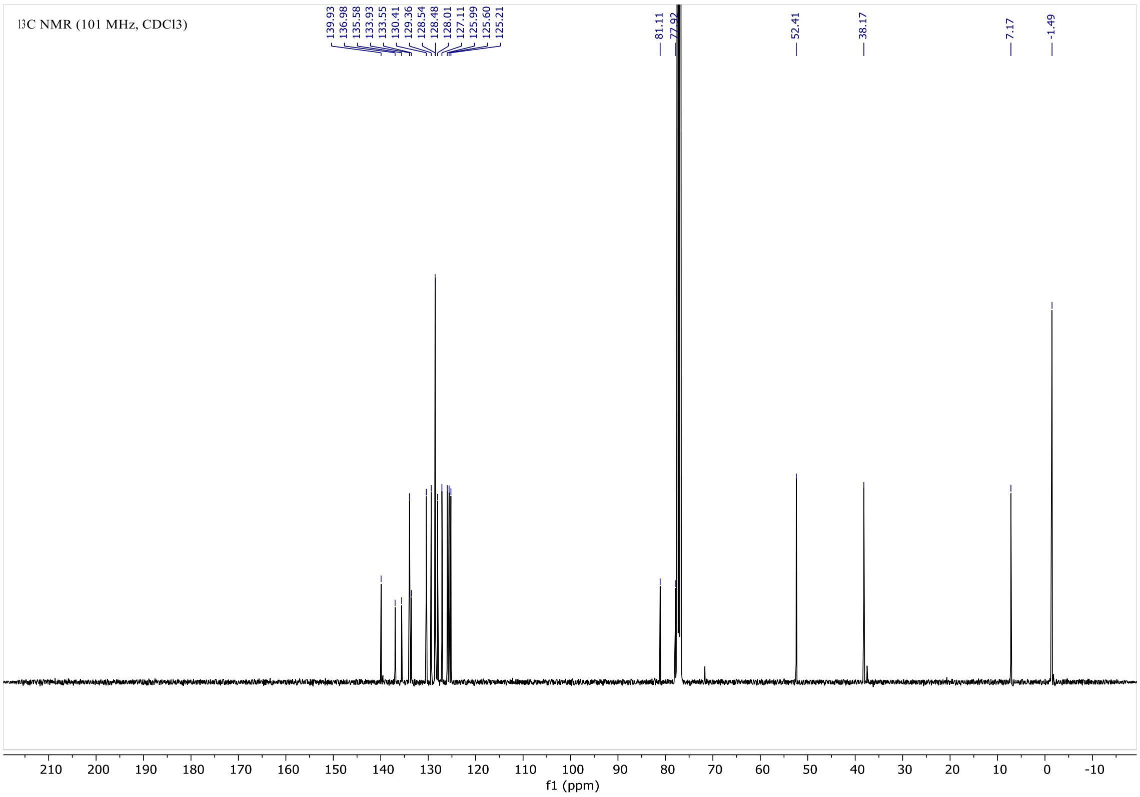
13C NMR (101 MHz, CDCl3)
139.93
136.98
135.58
133.93
133.55
130.41
129.36
128.54
128.48
128.01
127.11
125.99
125.60
125.21
81.11
77.92
52.41
38.17
7.17
-1.49
f1 (ppm)

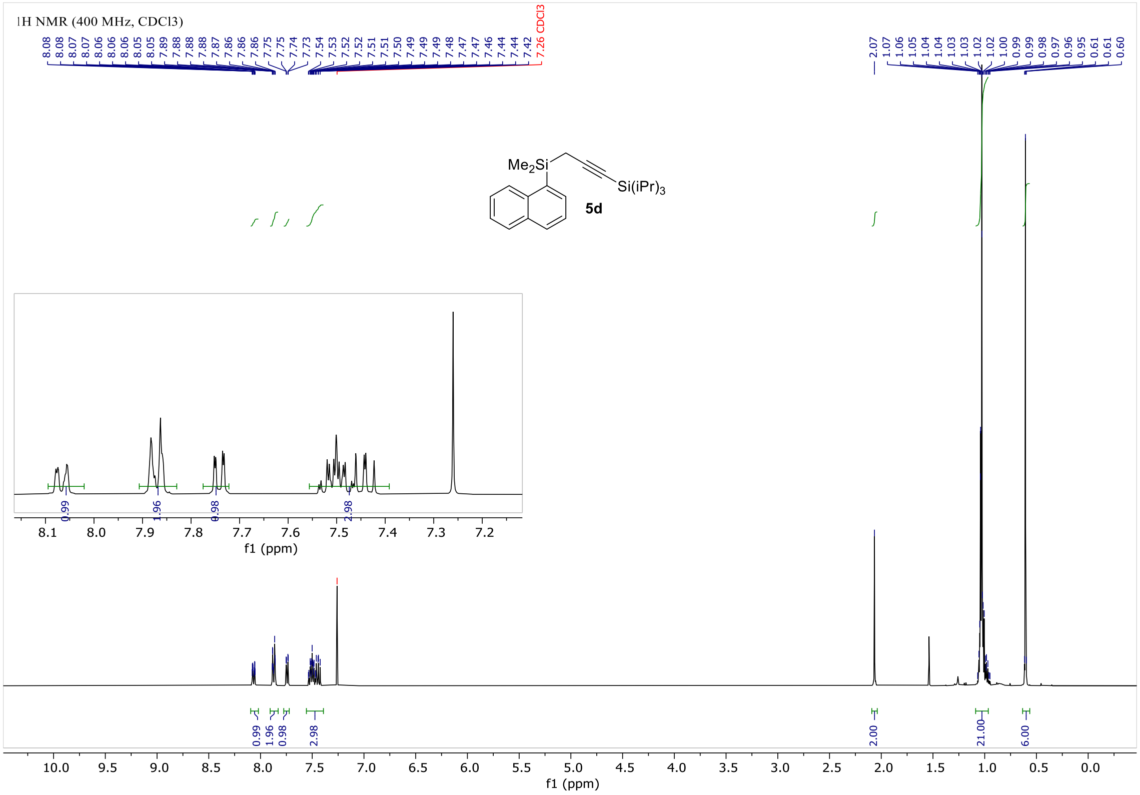
1H NMR (400 MHz, CDCl3)
7.26 CDCl3
Me2Si
Si(iPr)3
5d
f1 (ppm)
0.99
1.96
0.98
2.98
2.00
21.00
6.00

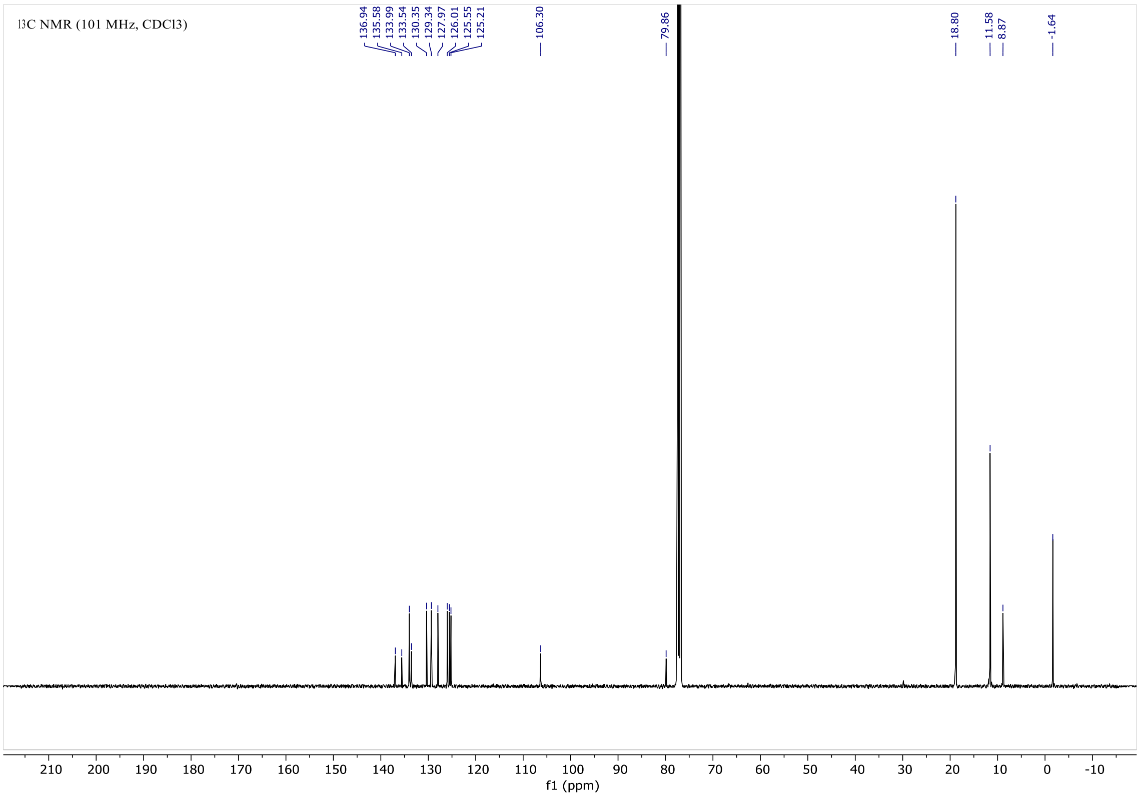
13C NMR (101 MHz, CDCl3)
136.94
135.58
133.99
133.54
130.35
129.34
127.97
126.01
125.55
125.21
106.30
79.86
18.80
11.58
8.87
-1.64
210
200
190
180
170
160
150
140
130
120
110
100
90
80
70
60
50
40
30
20
10
0
-10
f1 (ppm)

G.2. Product modification.

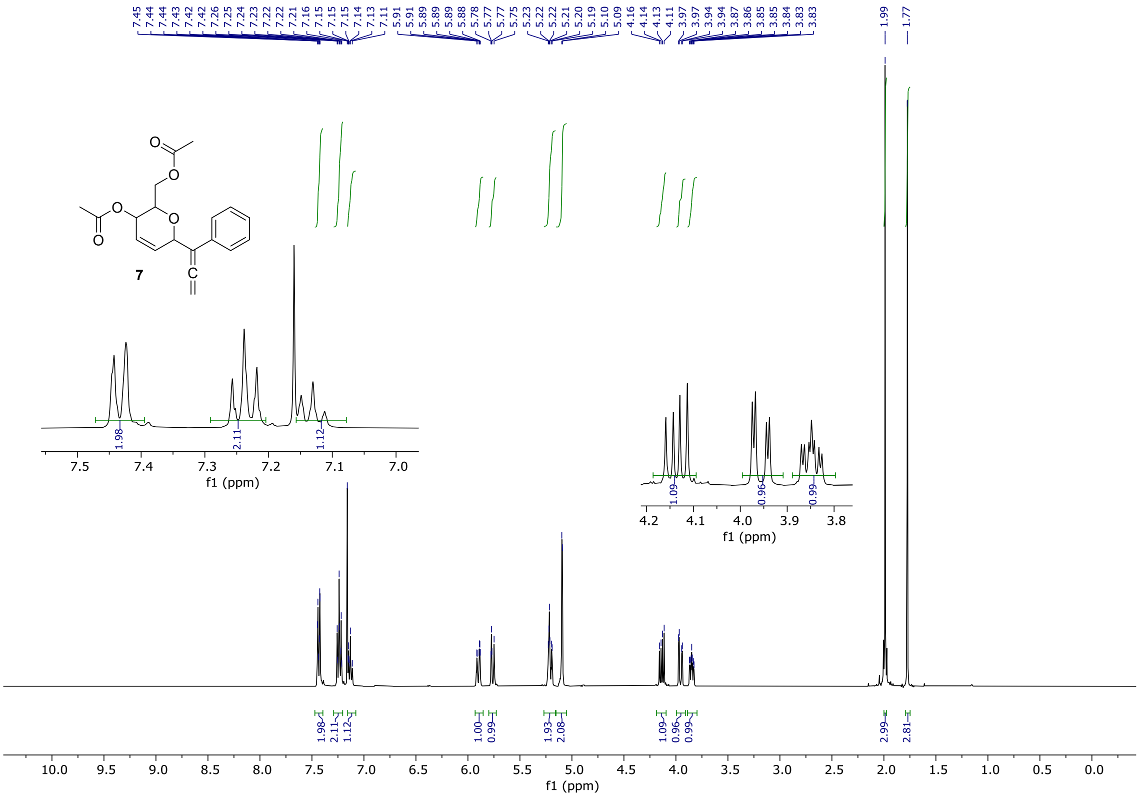

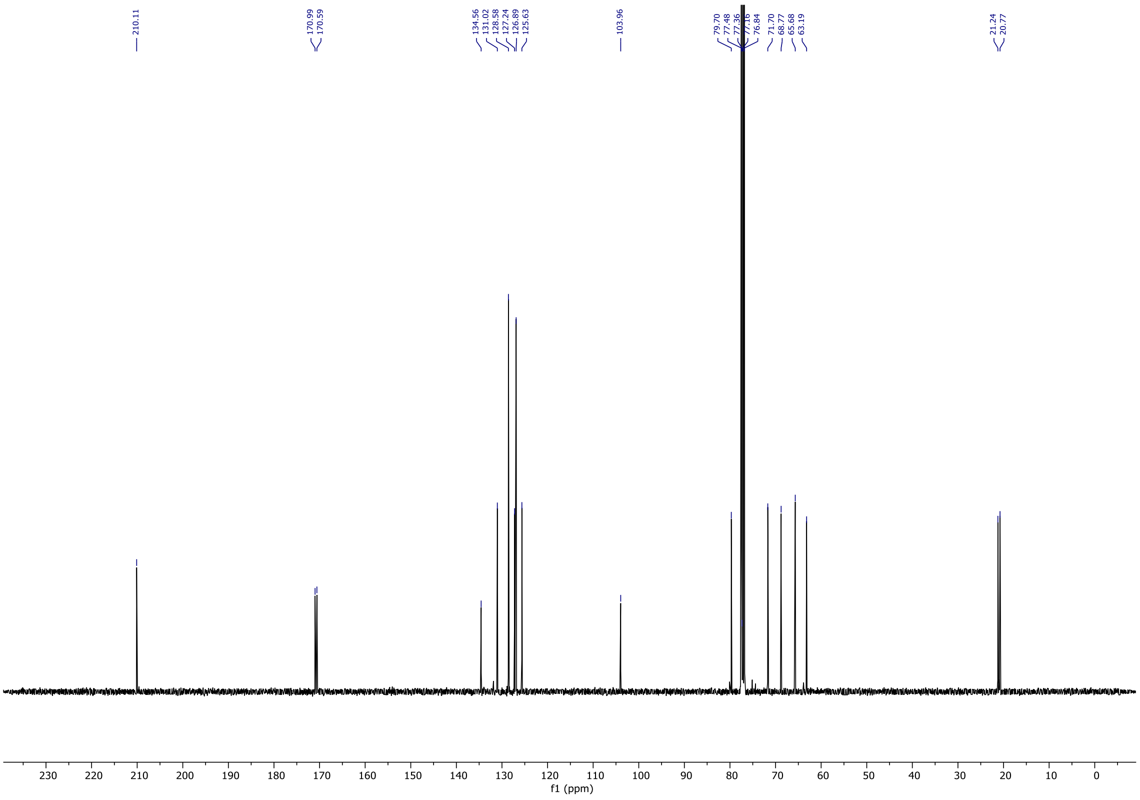

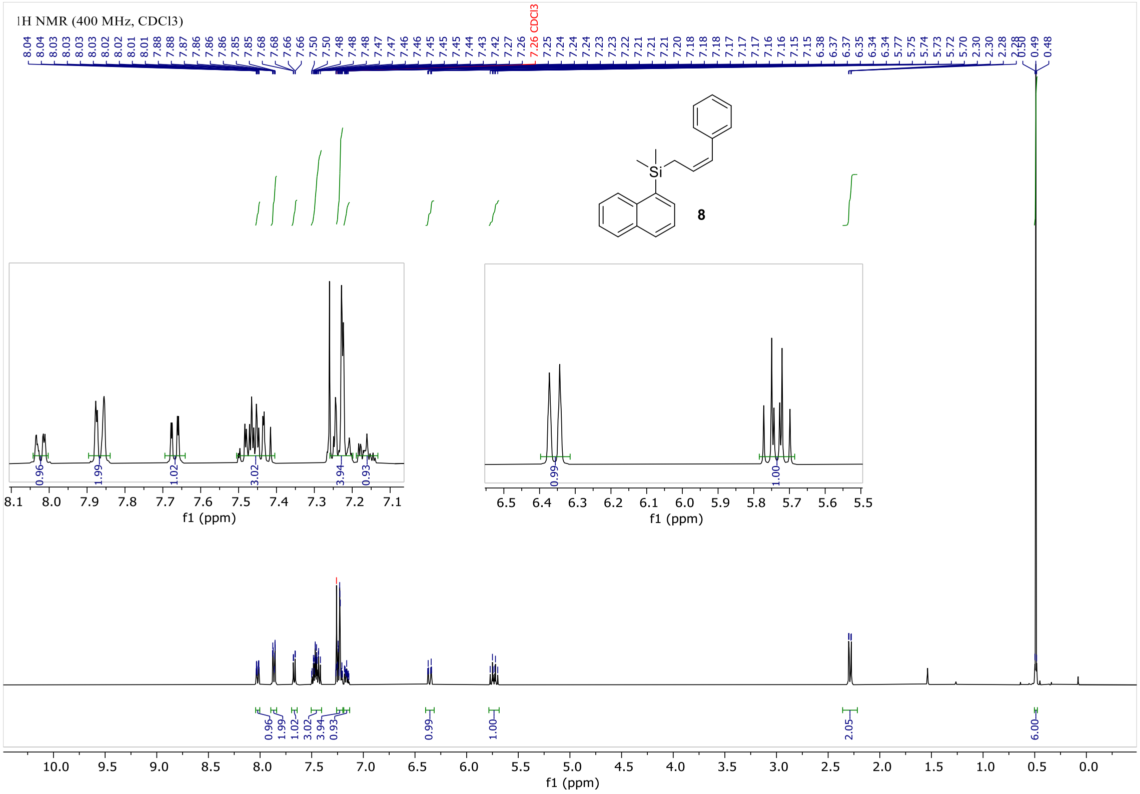
1H NMR (400 MHz, CDCl3)
8
f1 (ppm)

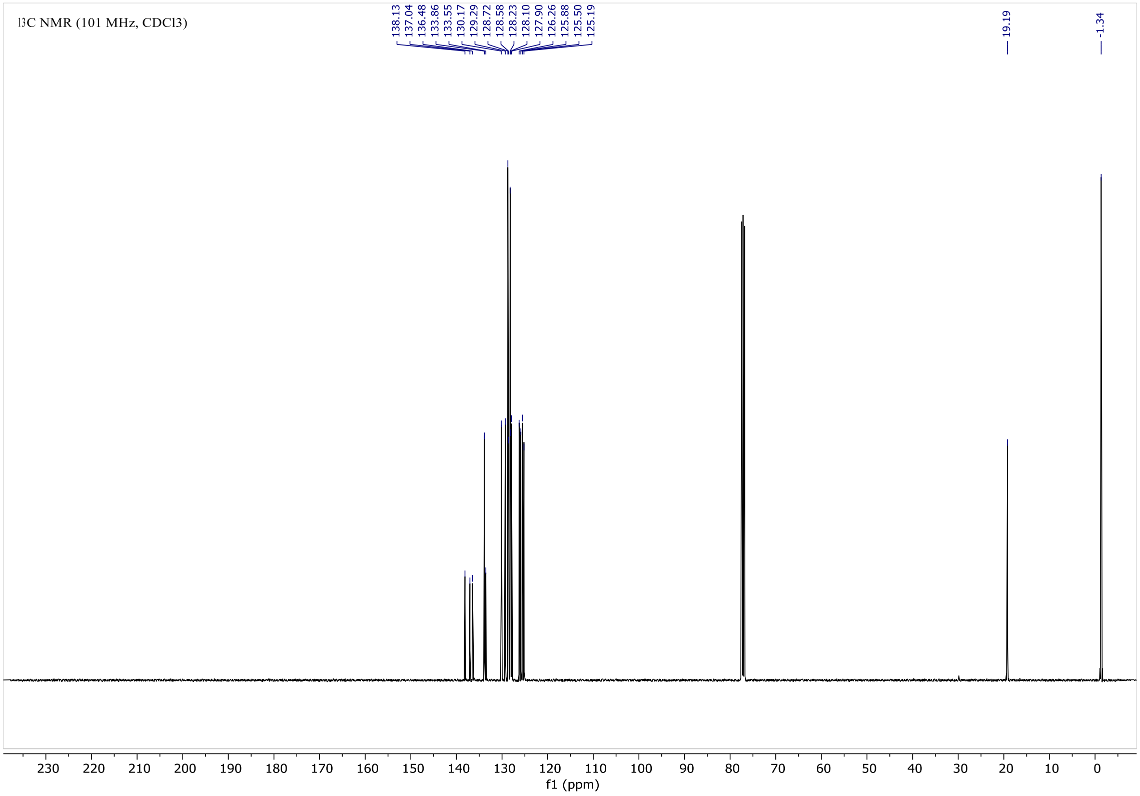
13C NMR (101 MHz, CDCl3)
138.13
137.04
136.48
133.86
133.55
130.17
129.29
128.72
128.58
128.23
128.10
127.90
126.26
125.88
125.50
125.19
19.19
-1.34
f1 (ppm)

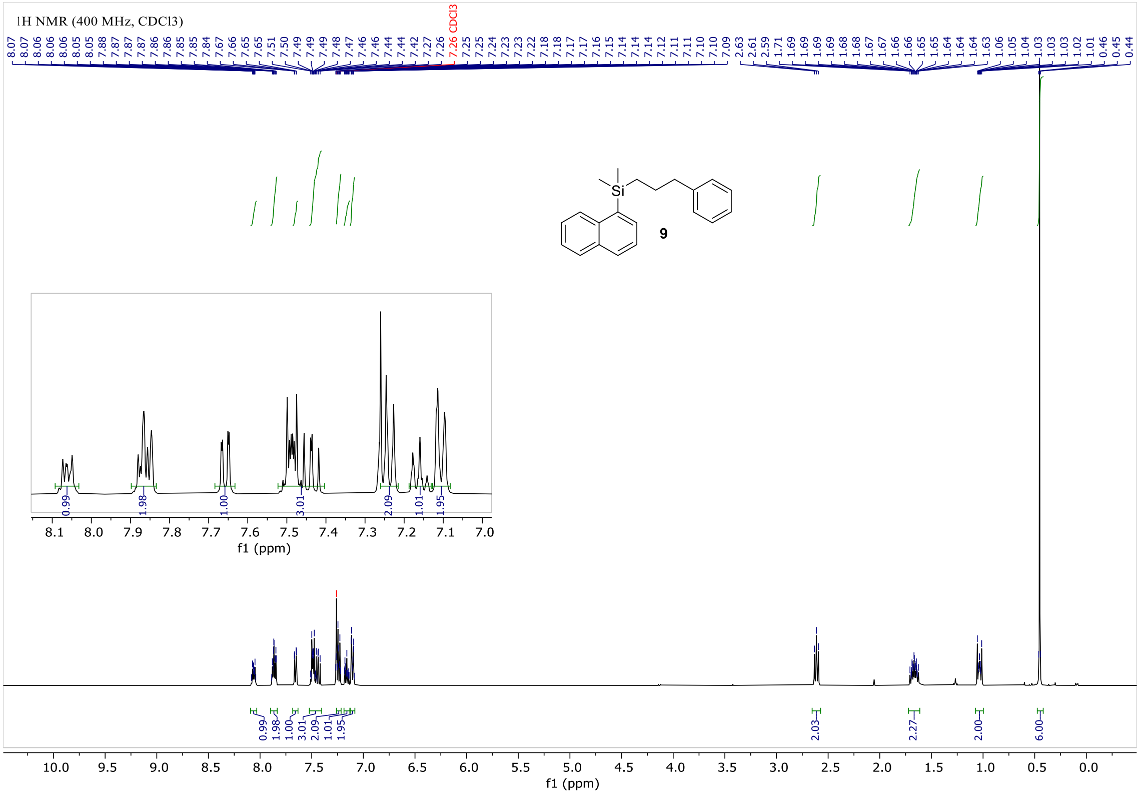
1H NMR (400 MHz, CDCl3)
Si
9
f1 (ppm)

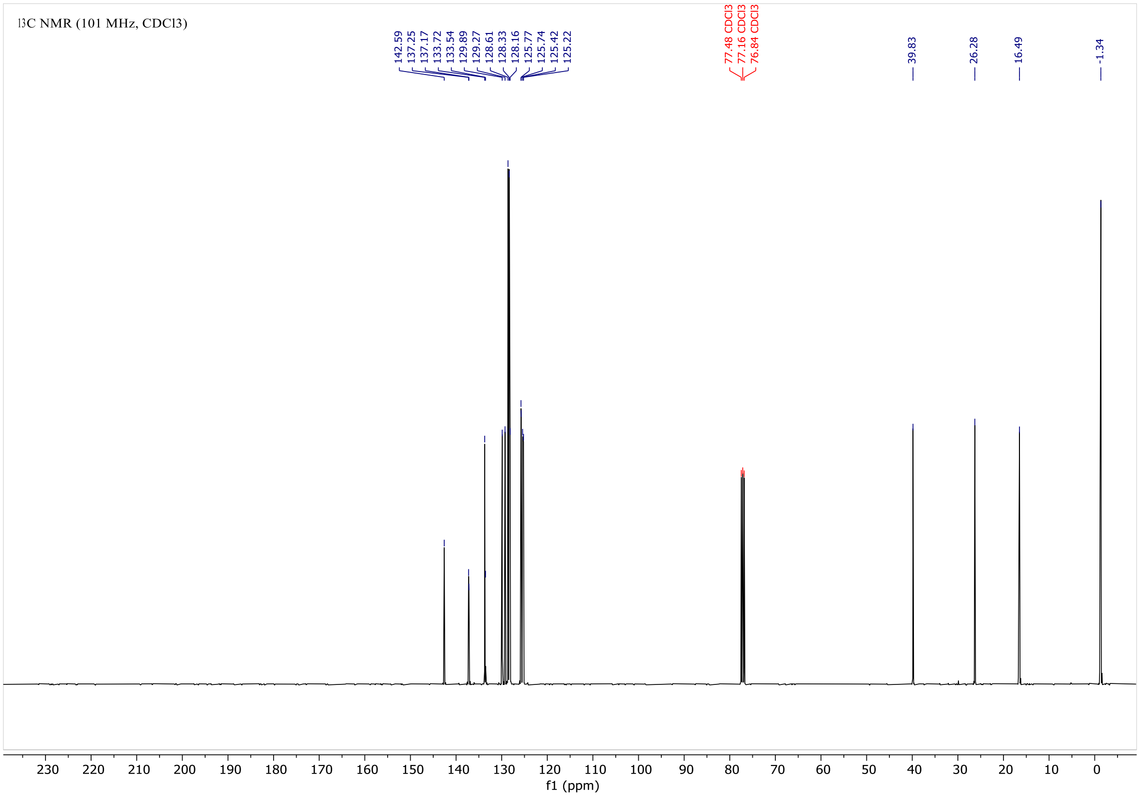
13C NMR (101 MHz, CDCl3)
142.59
137.25
137.17
133.72
133.54
129.89
129.27
128.61
128.33
128.16
125.77
125.74
125.42
125.22
77.48 CDCl3
77.16 CDCl3
76.84 CDCl3
39.83
26.28
16.49
-1.34
f1 (ppm)

NMR yield determination for **10a**:

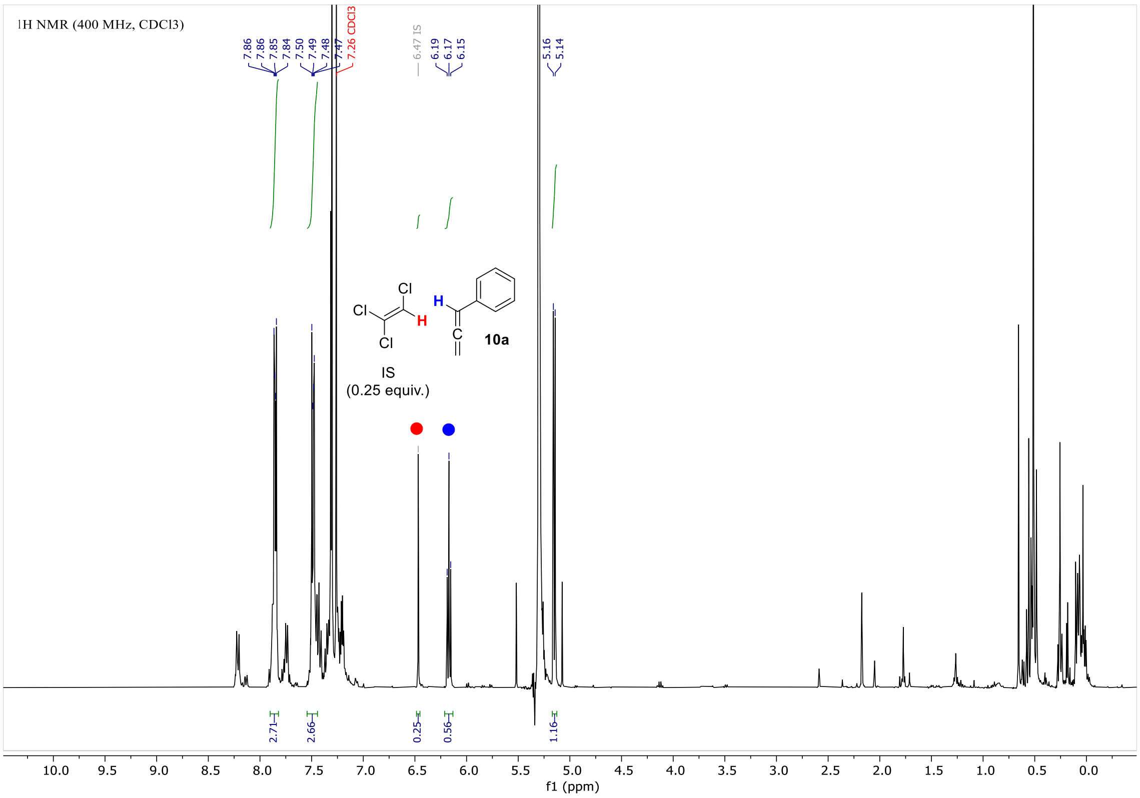

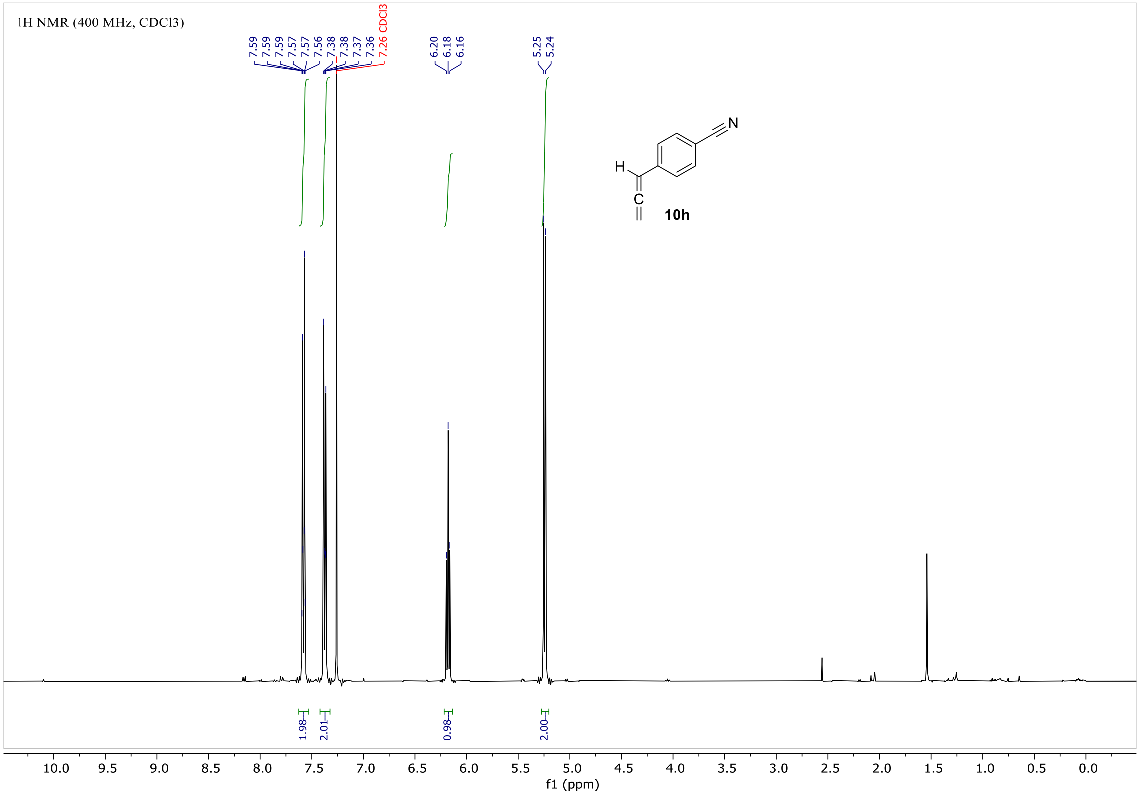

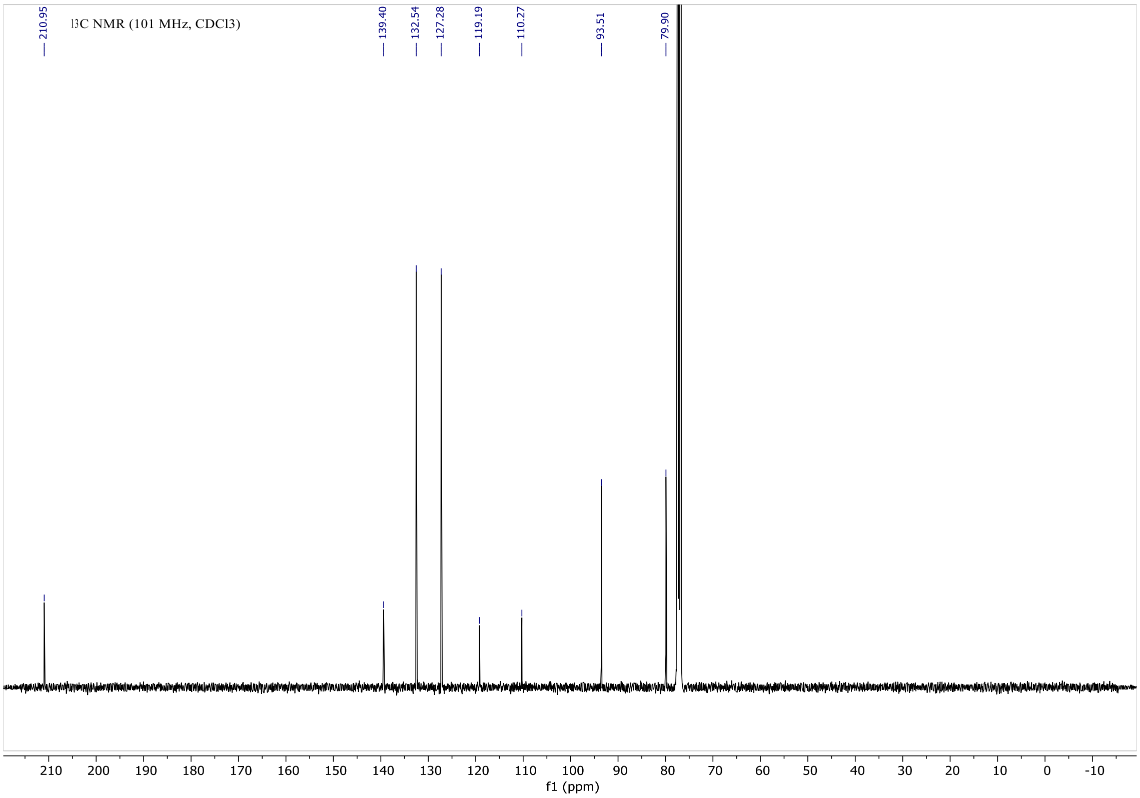
13C NMR (101 MHz, CDCl3)
210.95
139.40
132.54
127.28
119.19
110.27
93.51
79.90
f1 (ppm)

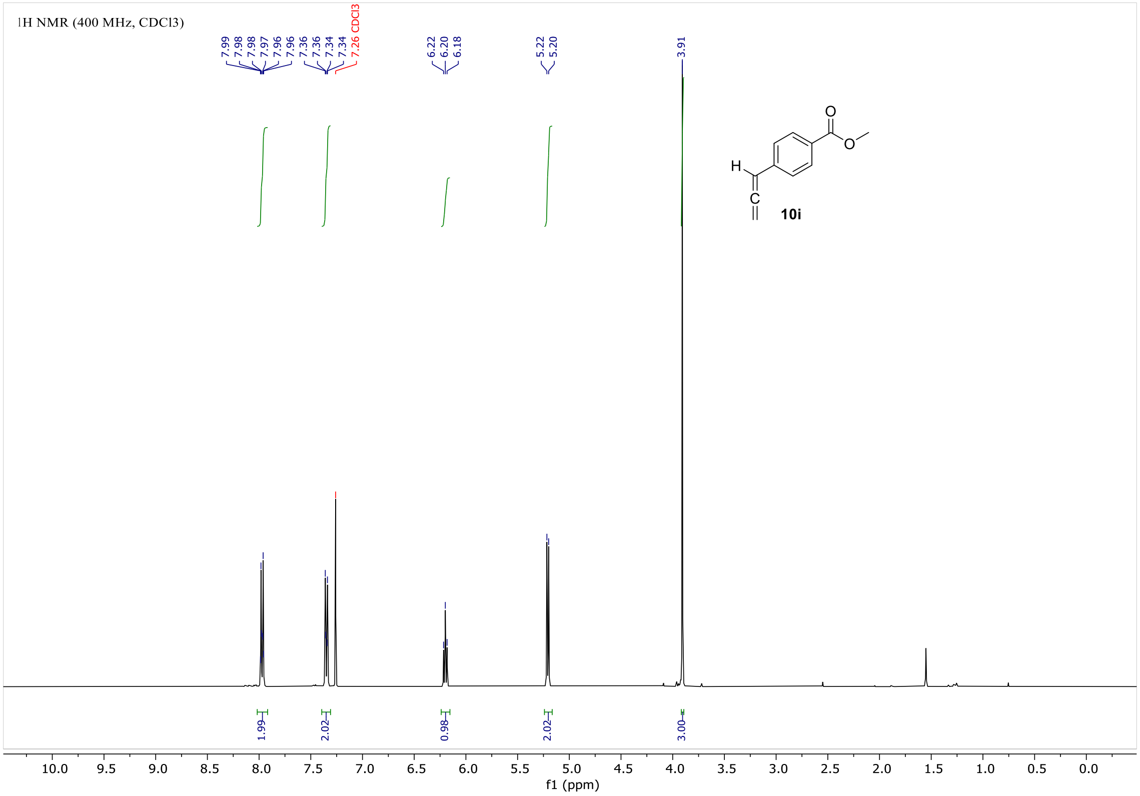
1H NMR (400 MHz, CDCl3)
7.99
7.98
7.98
7.97
7.96
7.96
7.36
7.36
7.34
7.34
7.26 CDCl3
6.22
6.20
6.18
5.22
5.20
3.91
1.99
2.02
0.98
2.02
3.00
f1 (ppm)
H
C
O
O
10i

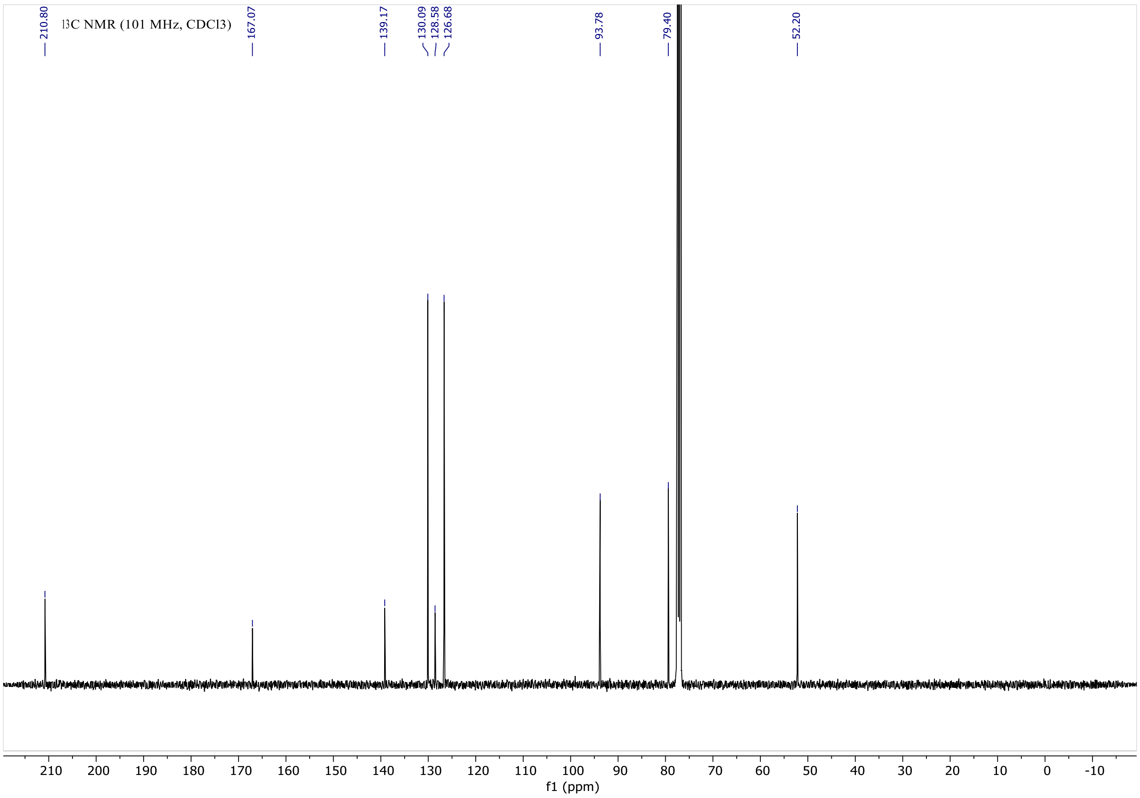
13C NMR (101 MHz, CDCl3)
210.80
167.07
139.17
130.09
128.58
126.68
93.78
79.40
52.20
f1 (ppm)

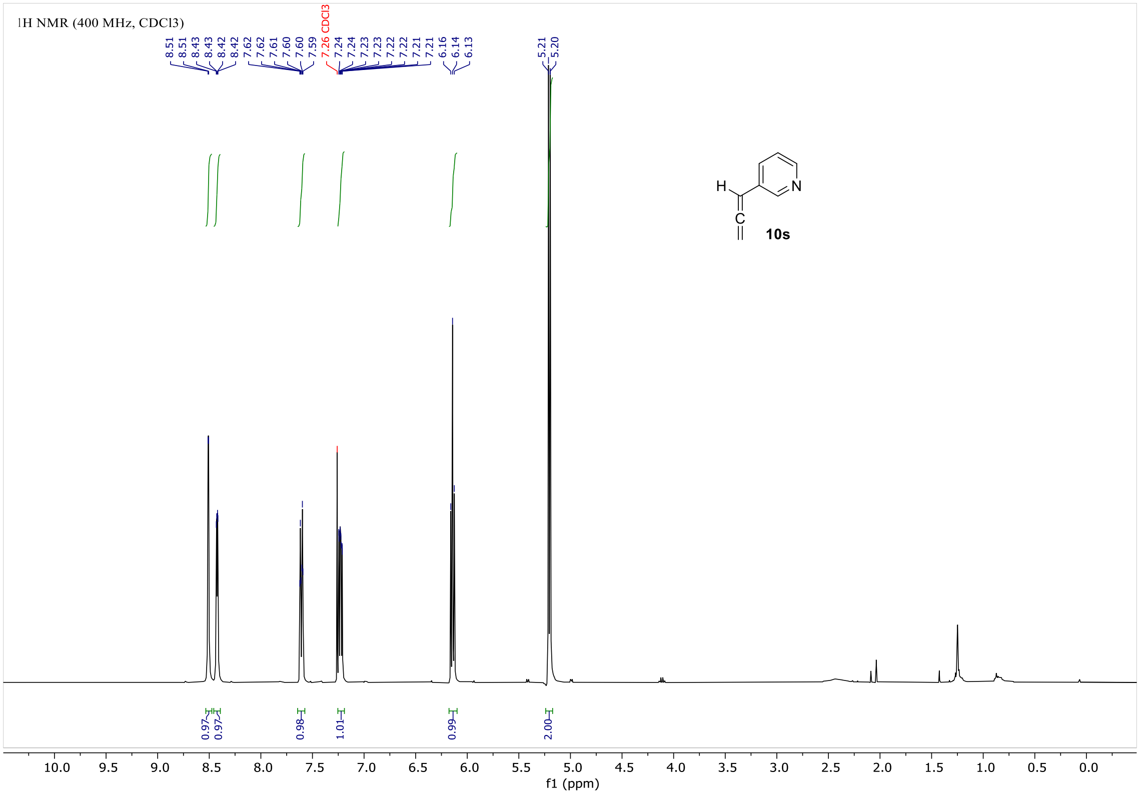
1H NMR (400 MHz, CDCl3)
8.51
8.51
8.43
8.43
8.42
8.42
7.62
7.62
7.61
7.60
7.60
7.59
7.26 CDCl3
7.24
7.24
7.23
7.23
7.22
7.22
7.21
7.21
6.16
6.14
6.13
5.21
5.20
H
N
C
10s
0.97
0.97
0.98
1.01
0.99
2.00
f1 (ppm)

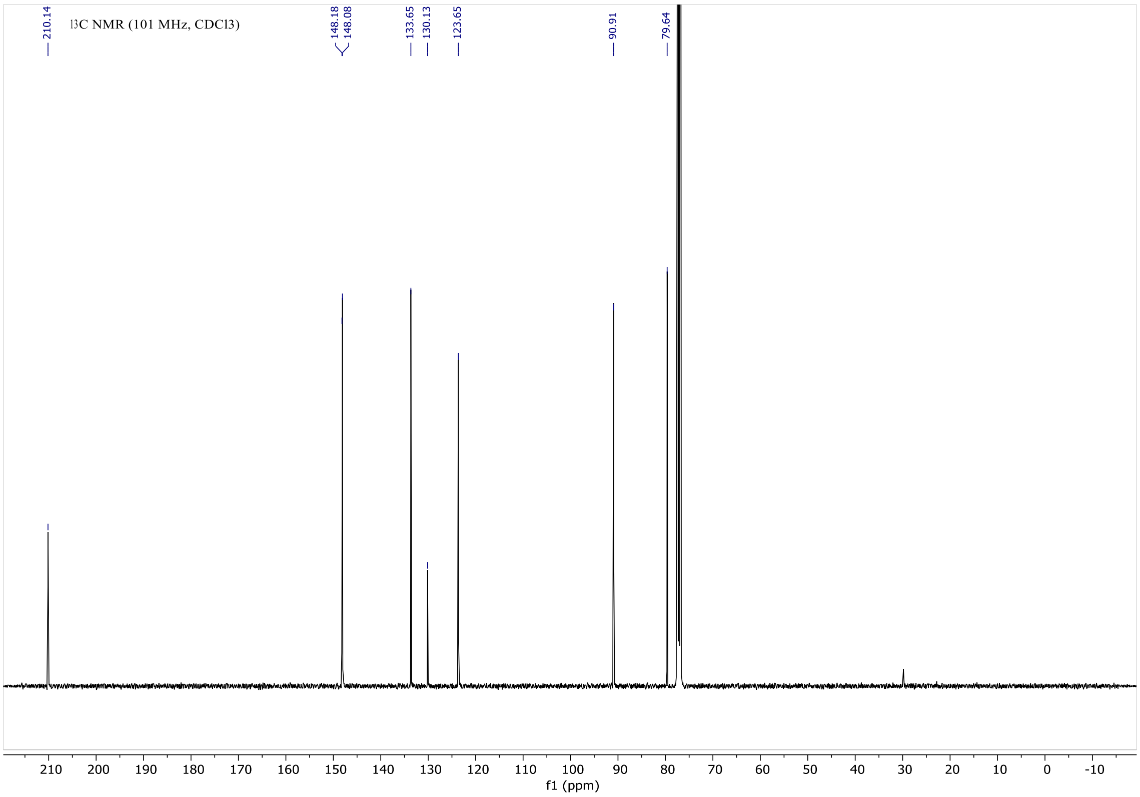

NMR yield determination for **10a and 11a**:

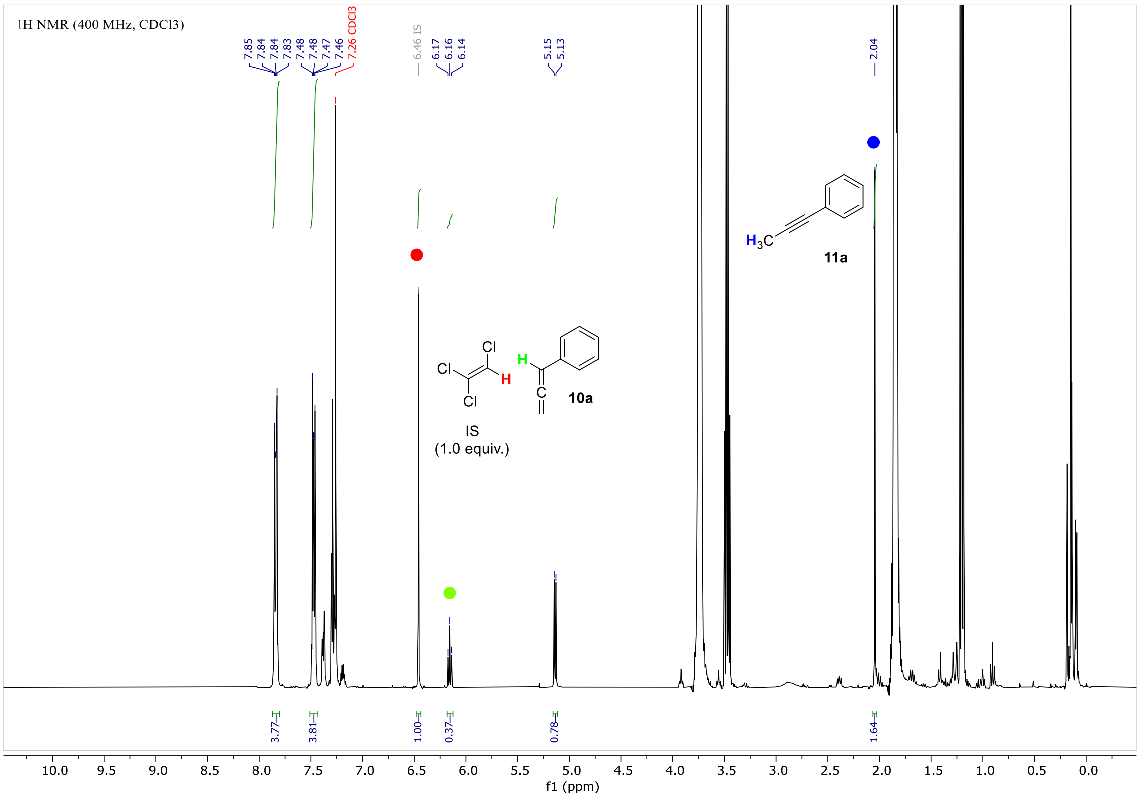

NMR yield determination for **10c and 11c**:

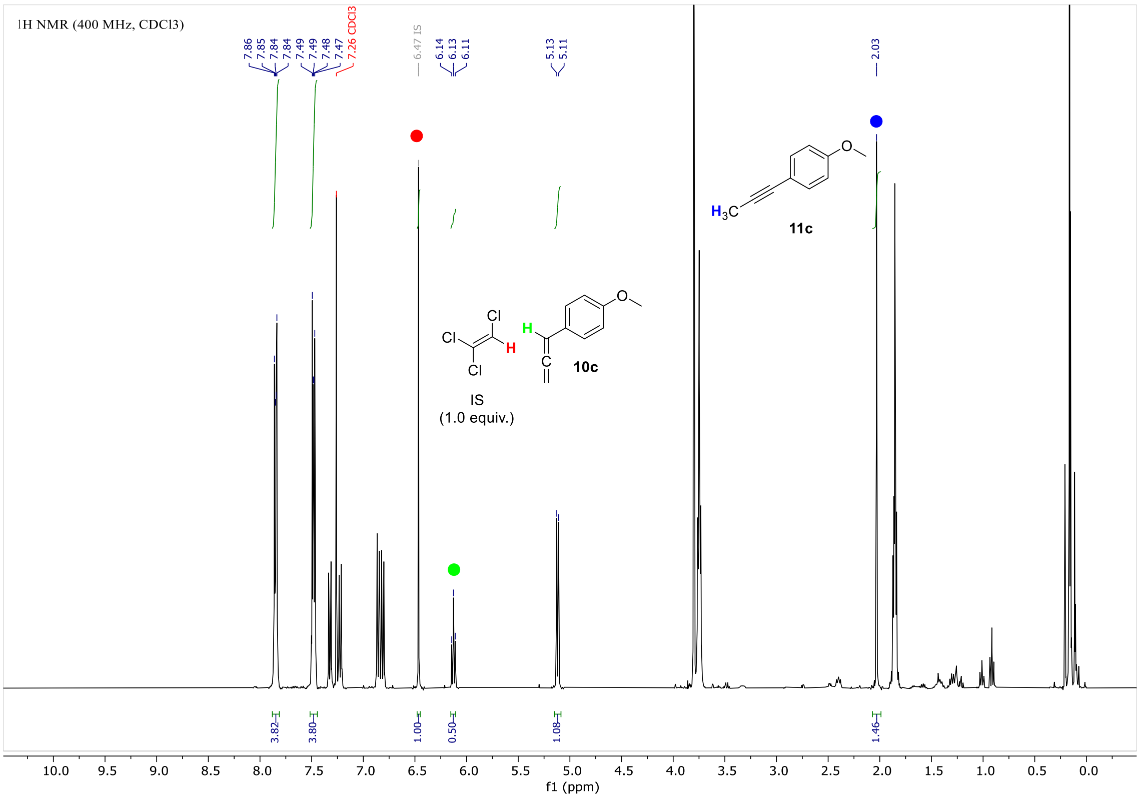

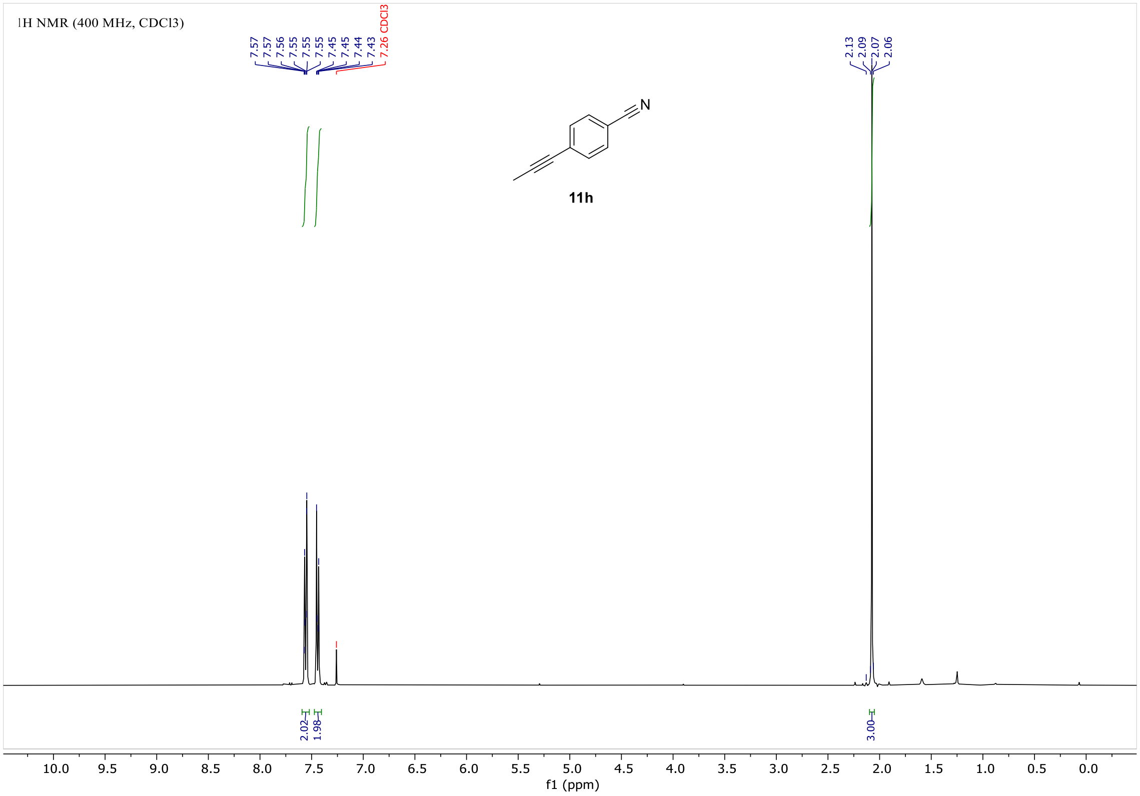

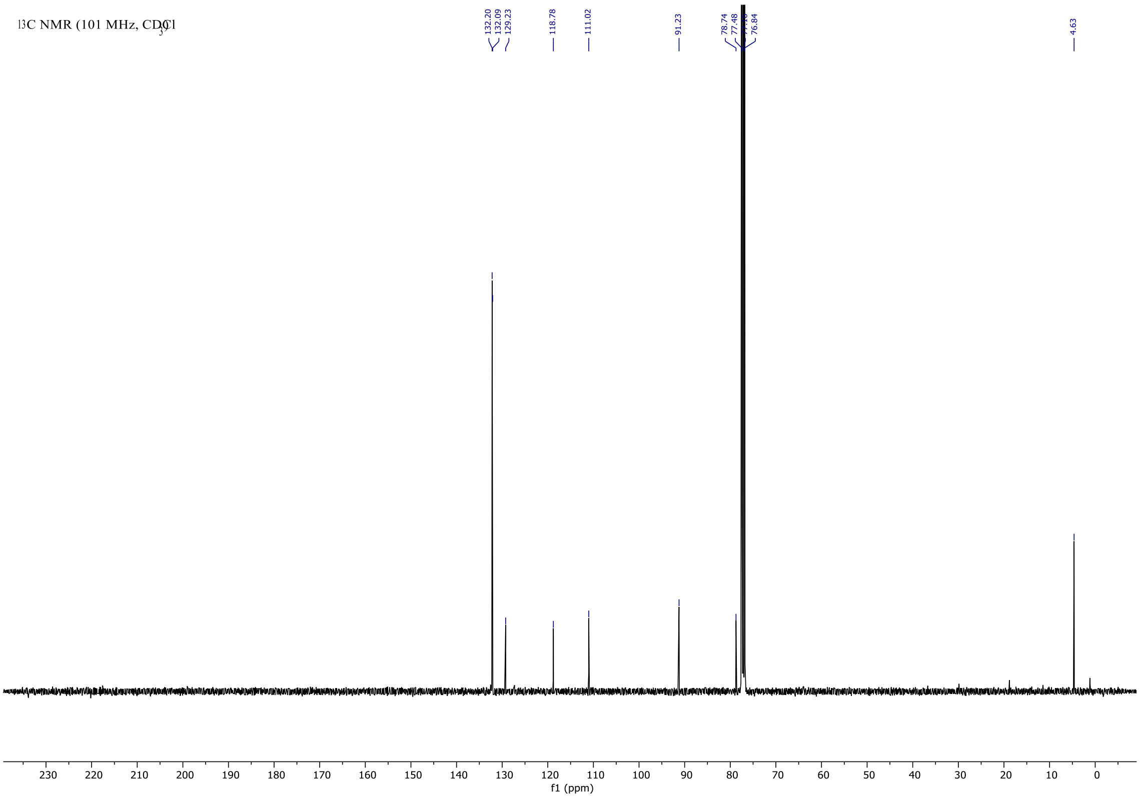
13C NMR (101 MHz, CDCl3)
132.20
132.09
129.23
118.78
111.02
91.23
78.74
77.48
76.84
4.63
f1 (ppm)

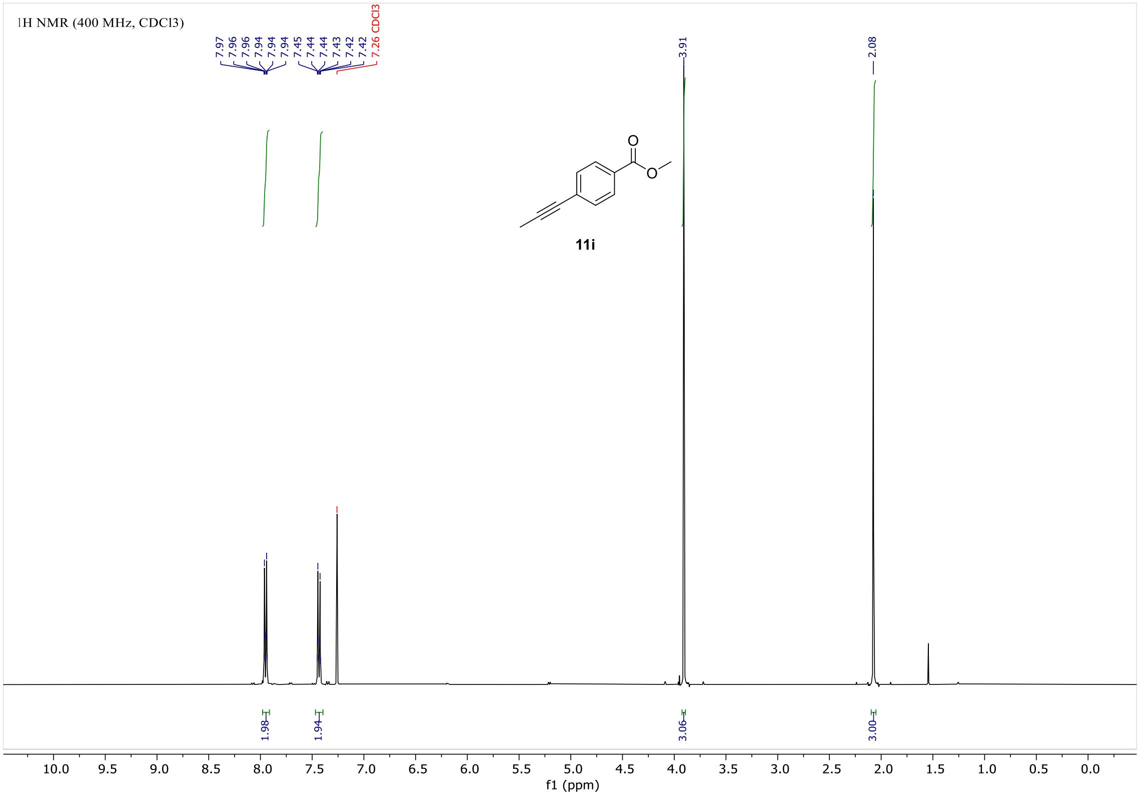
1H NMR (400 MHz, CDCl3)
7.97
7.96
7.96
7.94
7.94
7.94
7.45
7.44
7.44
7.43
7.42
7.42
7.26 CDCl3
3.91
2.08
11i
1.98
1.94
3.06
3.00
f1 (ppm)

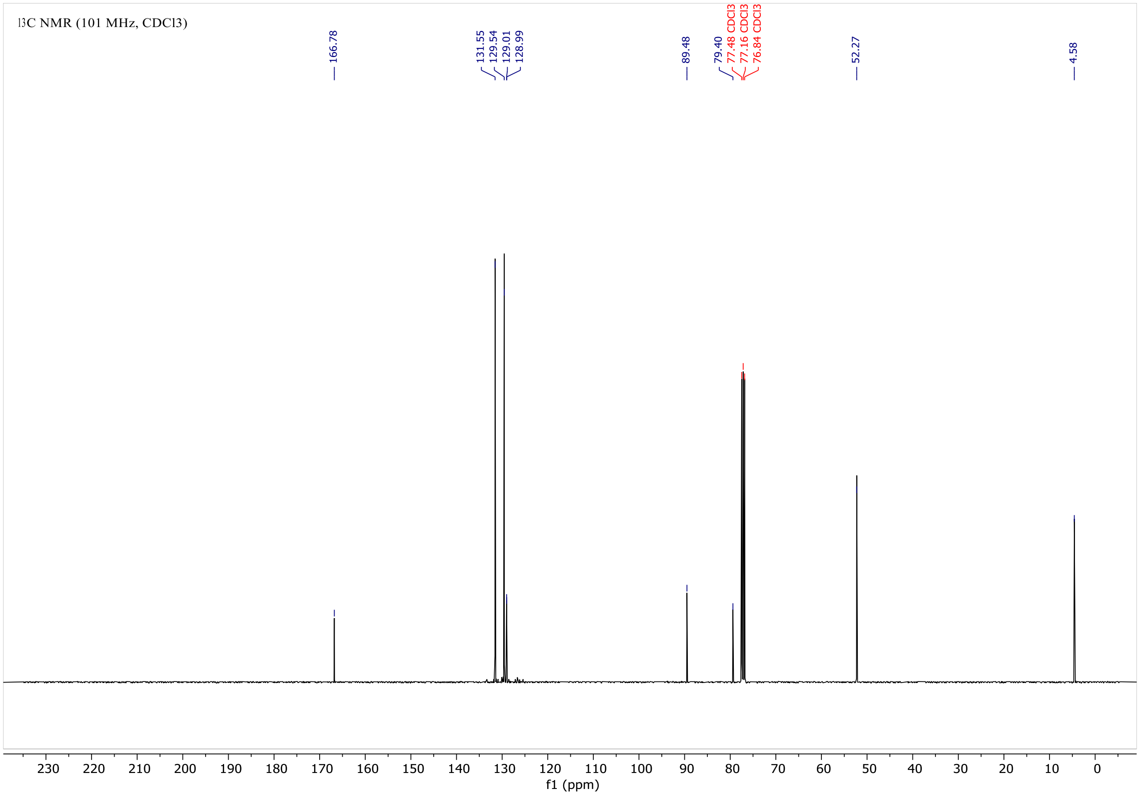
13C NMR (101 MHz, CDCl3)
166.78
131.55
129.54
129.01
128.99
89.48
79.40
77.48 CDCl3
77.16 CDCl3
76.84 CDCl3
52.27
4.58
230 220 210 200 190 180 170 160 150 140 130 120 110 100 90 80 70 60 50 40 30 20 10 0
f1 (ppm)

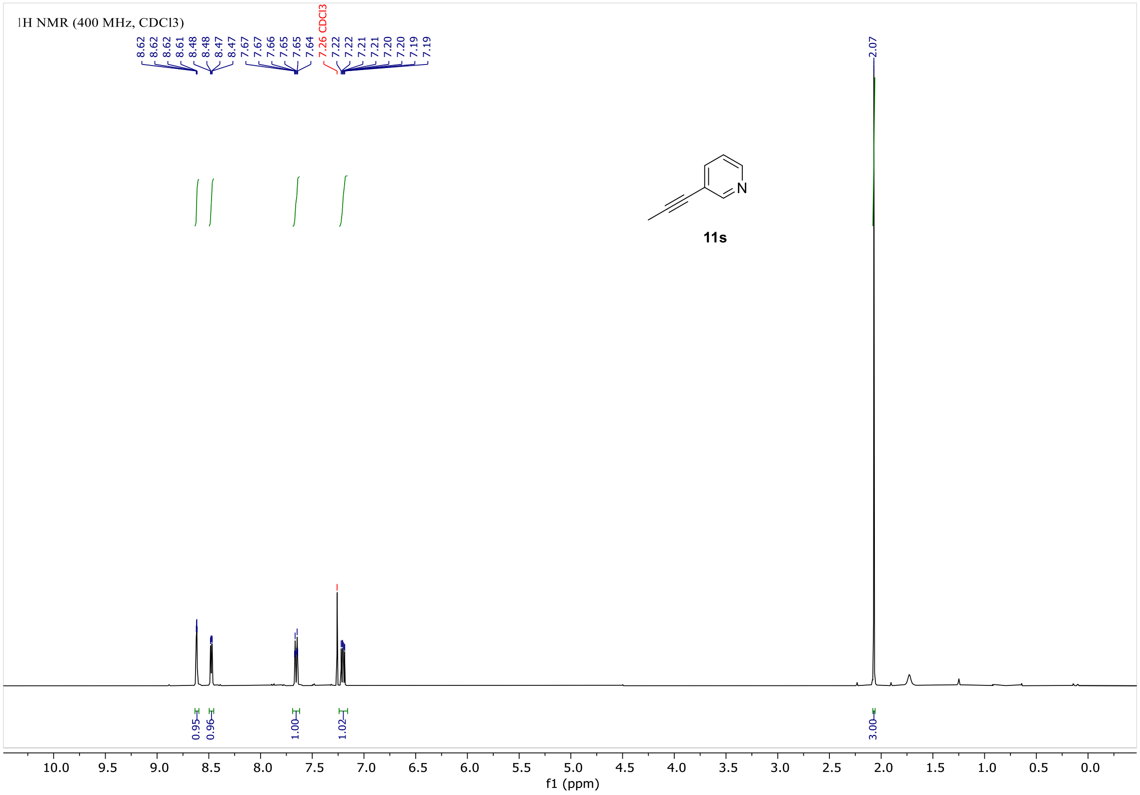
1H NMR (400 MHz, CDCl3)
8.62
8.62
8.62
8.61
8.48
8.48
8.47
8.47
7.67
7.67
7.66
7.65
7.65
7.64
7.26 CDCl3
7.22
7.22
7.21
7.21
7.20
7.20
7.19
7.19
2.07
N
11s
0.95
0.96
1.00
1.02
3.00
10.0 9.5 9.0 8.5 8.0 7.5 7.0 6.5 6.0 5.5 5.0 4.5 4.0 3.5 3.0 2.5 2.0 1.5 1.0 0.5 0.0
f1 (ppm)

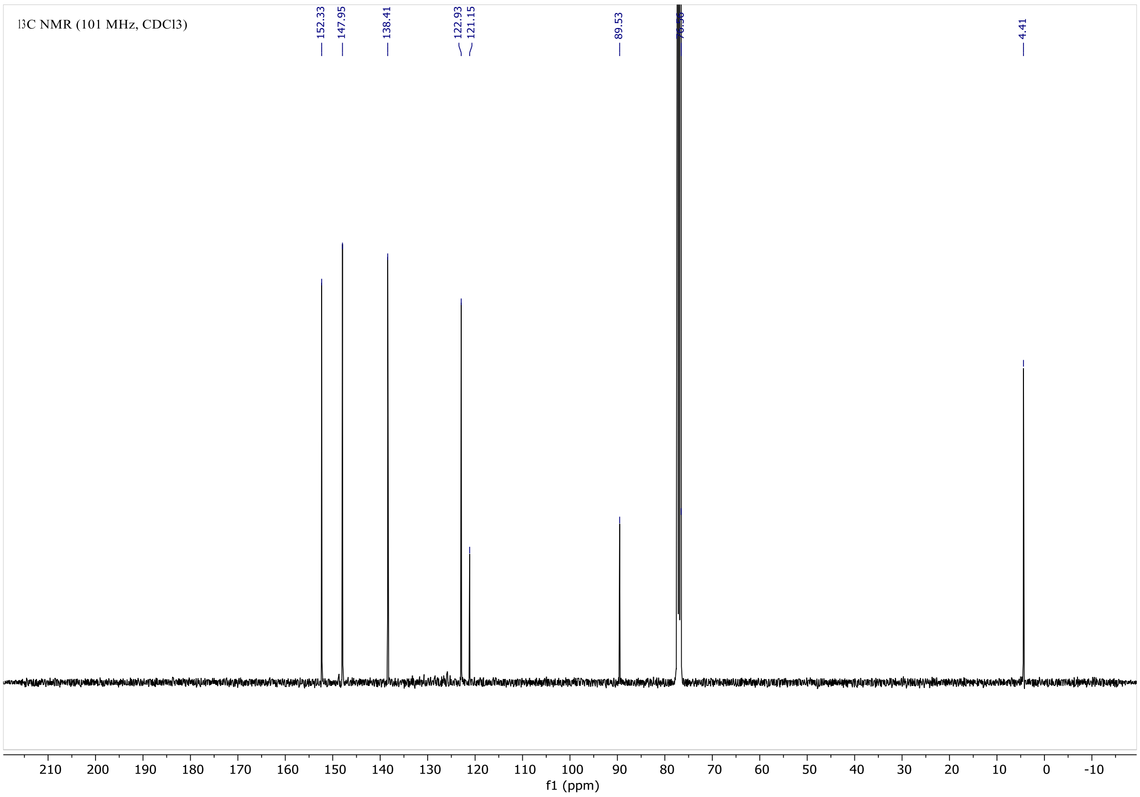
13C NMR (101 MHz, CDCl3)
152.33
147.95
138.41
122.93
121.15
89.53
4.41
210 200 190 180 170 160 150 140 130 120 110 100 90 80 70 60 50 40 30 20 10 0 -10
f1 (ppm)

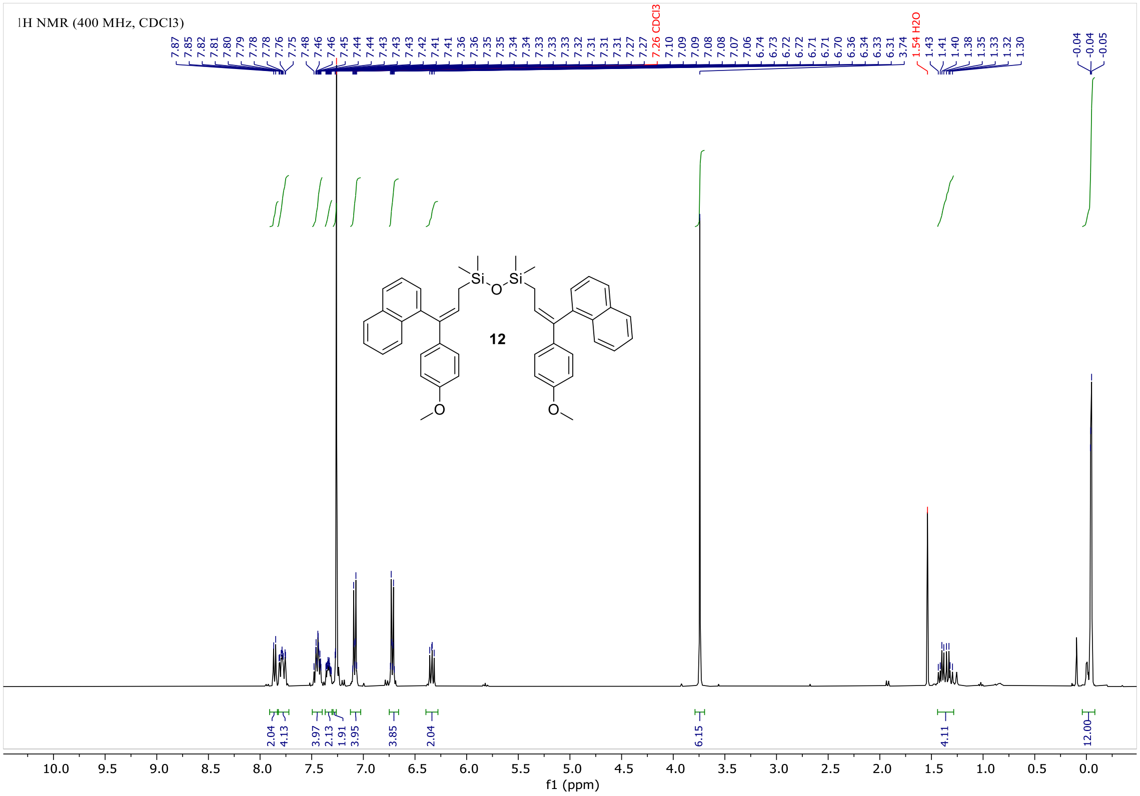
1H NMR (400 MHz, CDCl3)
7.87 7.85 7.82 7.81 7.80 7.79 7.78 7.78 7.76 7.75 7.48 7.46 7.46 7.45 7.44 7.44 7.43 7.43 7.43 7.42 7.41 7.41 7.36 7.36 7.35 7.35 7.34 7.34 7.33 7.33 7.32 7.31 7.31 7.27 7.27
7.26 CDCl3
7.10 7.09 7.09 7.08 7.08 7.07 7.06 6.74 6.73 6.72 6.72 6.71 6.70 6.36 6.34 6.33 6.31 3.74
1.54 H2O
1.43 1.41 1.40 1.38 1.35 1.33 1.32 1.30
-0.04 -0.04 -0.05
Si
O
Si
12
O
O
2.04 4.13 3.97 2.13 1.91 3.95 3.85 2.04 6.15 4.11 12.00
10.0 9.5 9.0 8.5 8.0 7.5 7.0 6.5 6.0 5.5 5.0 4.5 4.0 3.5 3.0 2.5 2.0 1.5 1.0 0.5 0.0
f1 (ppm)

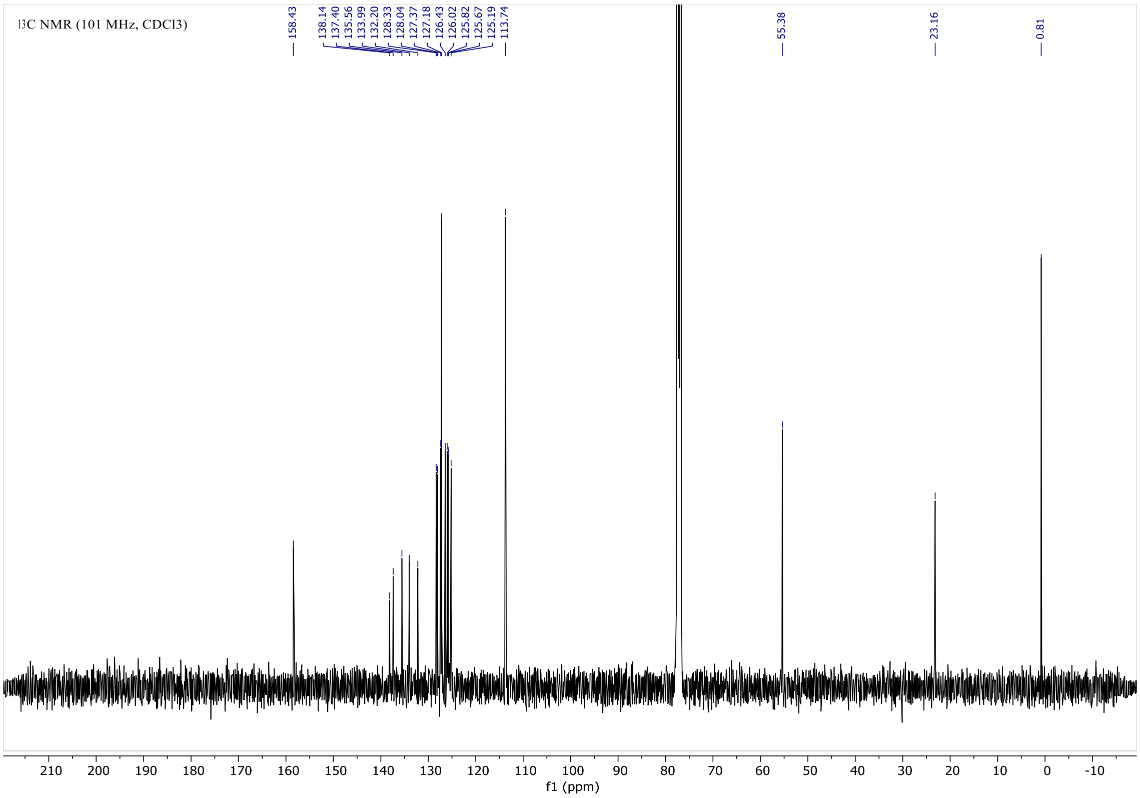
13C NMR (101 MHz, CDCl3)
158.43
138.14
137.40
135.56
133.99
132.20
128.33
128.04
127.37
127.18
126.43
126.02
125.82
125.67
125.19
113.74
55.38
23.16
0.81
f1 (ppm)

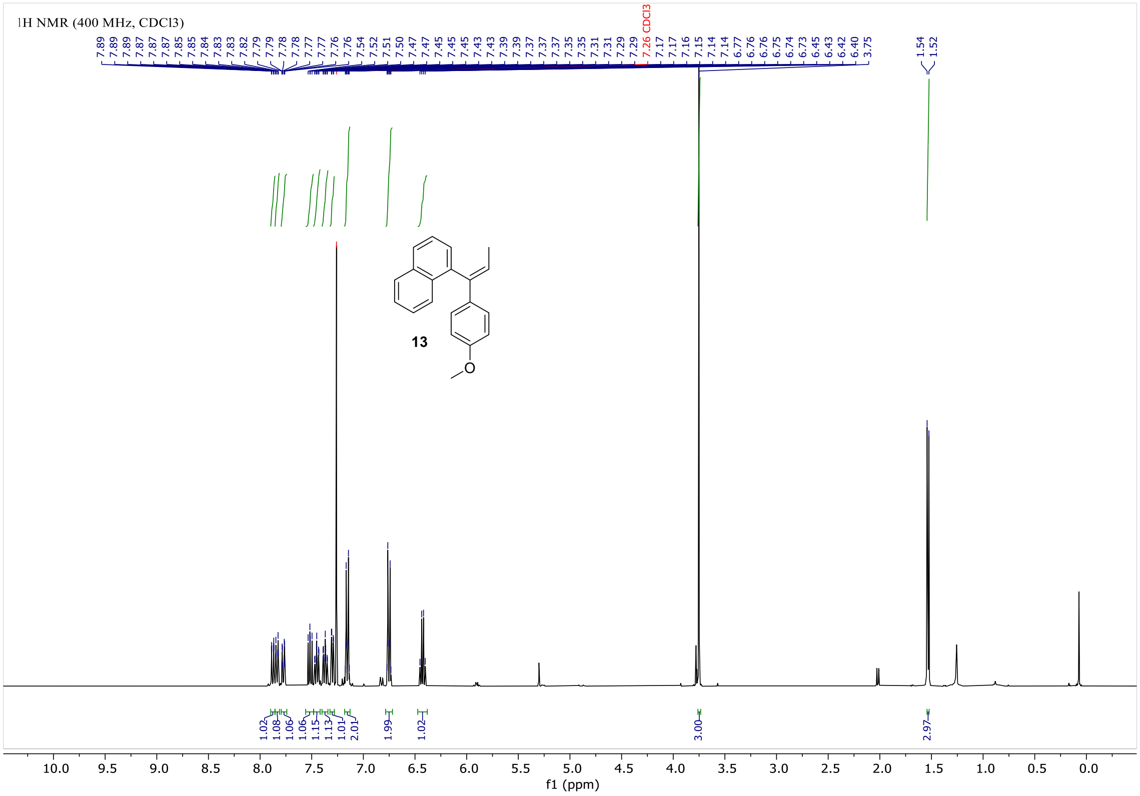
1H NMR (400 MHz, CDCl3)
13
f1 (ppm)

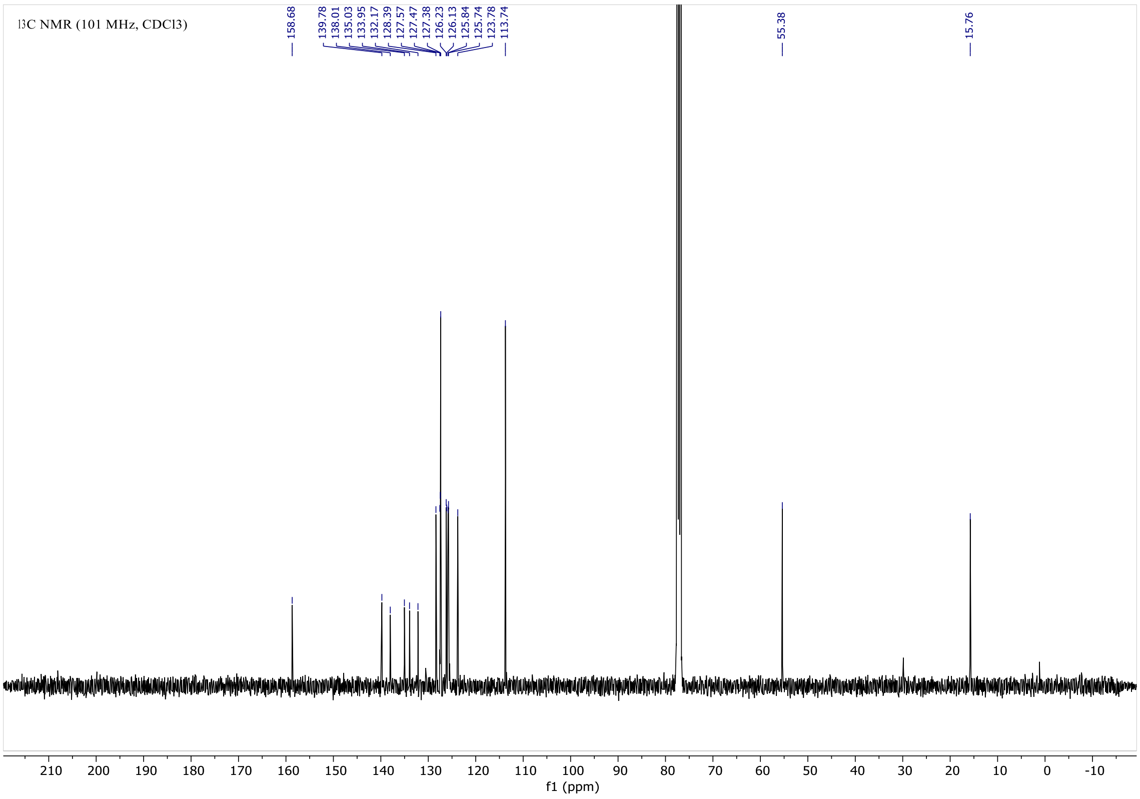

13C NMR (101 MHz, CDCl3)
158.68
139.78
138.01
135.03
133.95
132.17
128.39
127.57
127.47
127.38
126.23
126.13
125.84
125.74
123.78
113.74
55.38
15.76
210 200 190 180 170 160 150 140 130 120 110 100 90 80 70 60 50 40 30 20 10 0 -10
f1 (ppm)
